# Genetic contribution of *SCARB1* variants to lipid traits in African Blacks: a candidate gene association study

**DOI:** 10.1186/s12881-015-0250-6

**Published:** 2015-11-12

**Authors:** Vipavee Niemsiri, Xingbin Wang, Dilek Pirim, Zaheda H. Radwan, Clareann H. Bunker, M. Michael Barmada, M. Ilyas Kamboh, F. Yesim Demirci

**Affiliations:** 1 Department of Human Genetics, Graduate School of Public Health, University of Pittsburgh, 130 DeSoto Street, Pittsburgh, PA 15261 USA; 2 Department of Epidemiology, Graduate School of Public Health, University of Pittsburgh, 130 DeSoto Street, Pittsburgh, PA 15261 USA

**Keywords:** African continental ancestry group, Candidate gene association study, Genetic variation, Haplotypes, Lipids, SCARB1 protein, human, Sequence analysis, DNA

## Abstract

**Background:**

High-density lipoprotein cholesterol (HDL-C) exerts many anti-atherogenic properties including its role in reverse cholesterol transport (RCT). Scavenger receptor class B member 1 (SCARB1) plays a key role in RCT by selective uptake of HDL cholesteryl esters. We aimed to explore the genetic contribution of *SCARB1* to affecting lipid levels in African Blacks from Nigeria.

**Methods:**

We resequenced 13 exons and exon-intron boundaries of *SCARB1* in 95 individuals with extreme HDL-C levels using Sanger method. Then, we genotyped 147 selected variants (78 sequence variants, 69 HapMap tagSNPs, and 2 previously reported relevant variants) in the entire sample of 788 African Blacks using either the iPLEX Gold or TaqMan methods. A total of 137 successfully genotyped variants were further evaluated for association with major lipid traits.

**Results:**

The initial gene-based analysis demonstrated evidence of association with HDL-C and apolipoprotein A-I (ApoA-I). The follow-up single-site analysis revealed nominal evidence of novel associations of nine common variants with HDL-C and/or ApoA-I (*P* < 0.05). The strongest association was between rs11057851 and HDL-C (*P* = 0.0043), which remained significant after controlling for multiple testing using false discovery rate. Rare variant association testing revealed a group of 23 rare variants (frequencies ≤1 %) associated with HDL-C (*P* = 0.0478). Haplotype analysis identified four *SCARB1* regions associated with HDL-C (global *P* < 0.05).

**Conclusions:**

To our knowledge, this is the first report of a comprehensive association study of *SCARB1* variations with lipid traits in an African Black population. Our results showed the consistent association of *SCARB1* variants with HDL-C across various association analyses, supporting the role of *SCARB1* in lipoprotein-lipid regulatory mechanism.

**Electronic supplementary material:**

The online version of this article (doi:10.1186/s12881-015-0250-6) contains supplementary material, which is available to authorized users.

## Background

Abnormal lipid and lipoprotein levels are a major risk factor for coronary heart disease (CHD) [1], the leading cause of death worldwide [2]. Elevated low-density lipoprotein cholesterol (LDL-C) levels and decreased high-density lipoprotein cholesterol (HDL-C) levels are correlated with the development of CHD. There is a strong genetic basis for lipoprotein-lipid levels with heritability estimates of 40–80 % [3]. A large number of genes and genetic variants associated with lipid traits have been discovered in genome-wide association studies (GWAS) [4–6]. Most of the common variants (minor allele frequency [MAF] ≥5 %) identified by GWAS have modest effects on lipid levels, and have overall a small contribution to total genetic variance of lipid traits (~25–30 % of the heritability) [4–8]. A portion of the missing heritability of lipid traits could be explained by low frequency (LoF)/rare variants (MAF <5 %) as suggested by recent studies [9–11].

HDL, the smallest and densest (*d* = 1.063–1.21 g/mL) class of lipoprotein particles, has a variety of antiatherogenic properties [12]. One of the HDL properties to protect against CHD is mediated by reverse cholesterol transport (RCT) from peripheral tissues back to the liver [13]. Scavenger receptor class B member 1 (SCARB1, protein; *SCARB1*, gene) serves as a HDL-C receptor in RCT that mediates selective uptake of HDL-C cholesteryl esters (CE) by the liver and free cholesterol efflux from cells to HDL-C [14]. SCARB1 is also implicated in the metabolism of apolipoprotein B (ApoB)-containing particles [15–21].

The *SCARB1* gene (Entrez Gene ID: 949) is located on human chromosome 12, and is abundantly expressed in liver and steroidogenic tissues [22, 23]. The role of *SCARB1* in HDL-C and ApoB-containing lipoproteins metabolism has been established in animal studies. The disruption of *SCARB1* is associated with increased HDL-C levels and decreased CE uptake [24–26]. Whereas the overexpression of *SCARB1* reduces levels of HDL-C, ApoA-I, very low-density lipoprotein cholesterol (VLDL-C), LDL-C, and ApoB [15–17, 19] and promotes the hepatic uptake of CE as well as the biliary secretion of HDL-C [15, 27]. The *SCARB1* expression is also significantly associated with hepatic VLDL-triglycerides (TG) and VLDL-ApoB production. Hepatic VLDL cholesterol production together with VLDL clearance is enhanced in response to *SCARB1* overexpression [21]. In contrast, reduced hepatic VLDL-TG and VLDL-ApoB production is associated with *SCARB1* knockout status [18, 20, 21].

In humans, three *SCARB1* mutations (rs397514572 [p.Ser112Phe], rs187831231 [p.Thr175Ala], and rs387906791 [p.Pro297Ser]; MIM: 601040) have been reported to be associated with significantly increased HDL-C levels [28, 29]. Moreover, several genetic studies have demonstrated the association of common *SCARB1* variation with lipoprotein-lipid levels [5, 28–39] and subclinical atherosclerosis [40].

To our knowledge, no genetic study has exclusively investigated the association between *SCARB1* and lipid traits in native African populations to date. The objective of this study was to resequence all 13 exons and exon-intron boundaries of *SCARB1* in 95 African Blacks from Nigeria with extreme HDL-C levels for variant discovery and then to genotype selected variants in the entire sample of 788 African Blacks, followed by genotype-phenotype association analyses with five major lipid and apolipoprotein (Apo) traits (HDL-C, LDL-C, TG, ApoA-I and ApoB). Because our initial gene-based analysis demonstrated evidence of association with HDL-C and ApoA-I, our subsequent analyses focused on these two traits.

## Methods

### Study population

The present study was carried out on 788 African Black subjects from Benin City, Nigeria, who were recruited as part of a population-based epidemiological study on CHD risk factors. Detailed information on the study design and population description is provided elsewhere [41]. In brief, 788 recruited subjects were healthy civil servants (37.18 % females) from three government ministries of the Edo state in Benin City, Nigeria, aged between 19 and 70 years, including 464 junior staff (non-professional staff with salary grades 1–6), and 324 senior staff (professional and administrative staff with salary grades 7–16). The summary features, including biometric and quantitative data of the entire sample of 788 subjects are given in Table 1 and Additional file 1: Table S1.

**Table 1:** Characteristics and lipid profile of 95 individuals with extreme^a^ HDL-C levels and of the entire sample of 788 African Blacks

	95 Individuals with Extreme^a^ HDL-C Levels			The Entire Sample^b^
Variables	High HDL-C Group	Low HDL-C Group	*P* ^d^	
	(HDL-C range^c^: 68.30–99.00 mg/dL)	(HDL-C range^c^: 10.30–35.00 mg/dL)		
N (Females, n)	48 (24)	47 (24)	1.00	788 (293)
Age, years	41.29 ± 8.72	40.87 ± 7.12	0.80	40.95 ± 8.39
BMI, kg/m^2^	22.06 ± 4.70	23.91 ± 5.51	0.08	22.87 ± 4.04
Total Cholesterol, mg/dL	201.00 ± 39.68	141.68 ± 31.03	2.40E-12	172.01 ± 38.47
LDL-Cholesterol, mg/dL	112.55 ± 39.75	95.04 ± 28.28	0.02	109.25 ± 34.40
HDL-Cholesterol, mg/dL	76.05 ± 7.53	25.51 ± 5.66	2.20E-16	47.88 ± 12.87
Triglycerides, mg/dL	61.98 ± 19.85	95.79 ± 73.21	0.004	72.96 ± 39.32
Apolipoprotein A-I, mg/dL	166.04 ± 28.19	103.84 ± 27.23	2.20E-16	137.03 ± 28.46
Apolipoprotein B, mg/dL	66.00 ± 20.22	69.64 ± 21.46	0.40	66.98 ± 22.19

*BMI* body mass index, *HDL-C/HDL-Cholesterol* high-density lipoprotein cholesterol, *LDL-Cholesterol* low-density lipoprotein cholesterol

Values are presented as unadjusted means ± standard deviation (SD), unless otherwise mentioned

^a^Distribution of HDL-C was adjusted for sex and age: HDL-C levels ≥90th % tile defined as the “High HDL-C group”, and HDL-C levels ≤10th % tile defined as the “Low HDL-C group”

^b^All data were unadjusted and included individuals with missing values or outliers (values beyond mean ± 3.5 SD)

^c^Unadjusted range values

^d^Unadjusted *P*-values were calculated with t-test or *χ*^2^ test depending on types of variables

For resequencing, 95 individuals with extreme HDL-C levels (within the upper and lower 10th percentiles of HDL-C distribution) were chosen from the entire sample of 788 African Blacks. Resequencing sample comprised of 48 individuals with high HDL-C levels (≥90th percentile, range 68.30–99.00 mg/dL; Table 1) and 47 individuals with low HDL-C levels (≤10th percentile, range 10.30–35.00 mg/dL; Table 1). The University of Pittsburgh Institutional Review Board approved the study protocol. All participants gave their informed consent.

### Lipid and apolipoprotein measurements

At least 8-hour fasting blood samples were collected from all participants. Serum specimens were separated by centrifugation of blood samples and then stored at −70 °C for 6–12 months until ready for testing. Lipid and apolipoprotein measurements included total cholesterol, HDL-C, TG, ApoA-I, and ApoB and were done with standard assays at the Heinz Nutrition Laboratory, University of Pittsburgh under the Centers for Disease Control Lipid Standardization Program [41]. LDL-C was calculated with the Friedewald equation [42] when TG levels were less than 400 mg/dL.

### PCR and sequencing

Genomic DNA was isolated from clotted blood using the standard DNA extraction procedure. All 13 *SCARB1* exons (isoform 1, NM_005505), exon-intron boundaries, and 1 kb of each of 5′ and 3′ flanking regions on chromosome 12 (hg19, chr12: 125,262,175-125,348,519) were polymerase chain reaction (PCR) amplified and sequenced. Specific primers were designed using the Primer3 software (Whitehead Institute for Biomedical Research, http://bioinfo.ut.ee/primer3-0.4.0/) to cover 13 target regions, resulting in 14 amplicons, including two overlapping amplicons for the largest last exon 13. PCR reaction and cycling conditions are available upon request. The primer sequences and amplicon sizes are given in Additional file 2: Table S2.

Automated DNA sequencing of PCR products was performed in a commercial lab (Beckman Coulter Genomics, Danvers, MA, USA) using Sanger method and ABI 3730XL DNA Analyzers (Applied Biosystems, Waltham, MA, USA). Variant analysis was performed using Variant Reporter (version 1.0, Applied Biosystems, Waltham, MA, USA) and Sequencher (version 4.8, Gene Codes Corporation, Ann Arbor, MI, USA) software in our laboratory.

### Variant selection for genotyping

Of 83 variants identified in the discovery step (see Additional file 3: Table S3, Additional file 4: Table S4, Additional file 5: Figure S1, and Additional file 6: Figure S2), 78 (28 with MAF ≥5 % and 50 with MAF <5 %) were selected based on the pairwise linkage disequilibrium (LD) and Tagger analysis using an *r*^*2*^ threshold of 0.90 (5 were excluded due to high LD) in Haploview (Broad Institute of MIT and Harvard, https://www.broadinstitute.org/scientific-community/science/programs/medical-and-population-genetics/haploview/haploview) [43] for follow-up genotyping in the entire sample (*n* = 788). Since our sequencing was focused primarily on coding regions, in addition we selected 69 HapMap tag single nucleotide polymorphisms [SNPs] (out of total 108 HapMap tagSNPs; see Additional file 7: Table S5 and Additional file 8: Figure S3) based on Tagger analysis (MAF ≥5 % and *r*^*2*^ ≥ 0.80) of HapMap data (Release #27) from the Yoruba people of Ibadan, Nigeria (YRI), in order to cover the entire gene for common genetic variation information. Moreover, we selected two *SCARB1* variants previously reported to be significantly associated with lipid traits in the literature (Additional file 9: Table S6). Conclusively, a total of 149 variants, comprising of 78 sequence variants, 69 common HapMap-YRI tagSNPs, and two relevant associated variants, were selected for follow-up genotyping.

### Genotyping

Genotyping of selected variants in the total sample of 788 individuals was performed by using either iPLEX Gold (Sequenom, Inc., San Diego, CA, USA) or TaqMan (Applied Biosystems, Waltham, MA, USA) methods and following the manufacturers’ protocols.

Out of 149 selected variants, two failed assay designs and nine failed genotyping runs (see details in Additional file 3: Table S3, Additional file 7: Table S5, and Additional file 9: Table S6). Quality control (QC) measures for successfully genotyped variants were as follow: a genotype call rate of ≥90 %, a discrepancy rate of <1 in 10 % replicates, and no deviation from Hardy-Weinberg equilibrium [HWE] (*P* >3.62 × 10^−4^ after Bonferroni correction). Ultimately, a total of 137 QC-passed genotyped variants were included in genetic association analyses (see Additional file 9: Table S6, Additional file 10: Table S7, Additional file 11: Figure S4, and Additional file 12: Figure S5).

### Statistical analysis

We used the Haploview program to determine allele frequencies, to test HWE for genotype distribution, and to evaluate the LD and pairwise correlations (*r*^2^) between variants [43].

**Fig. 1:**
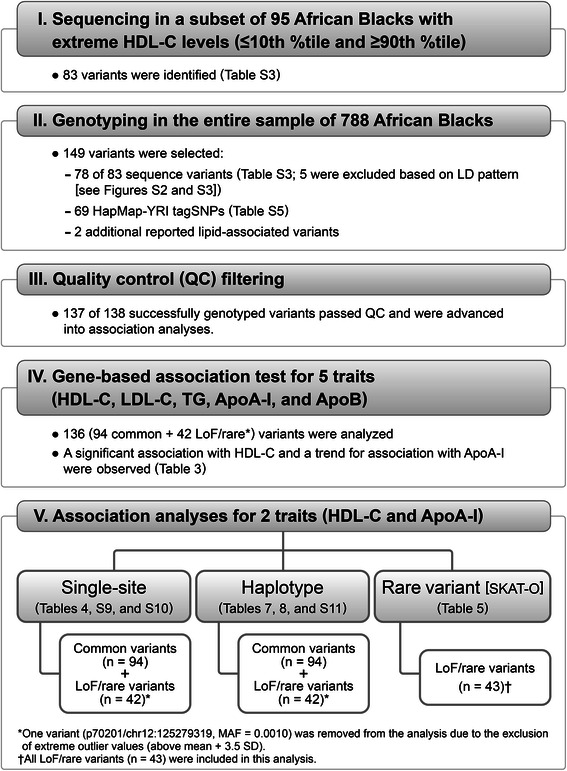
Summary of the study design and flow. Chart presents an overview of the study design and flow, including sequencing and genotyping stages and analysis approaches. ApoA-I, apolipoprotein A-I; ApoB, apolipoprotein B; HDL-C, high-density lipoprotein cholesterol; LD, linkage disequilibrium; LDL-C, low-density lipoprotein cholesterol; LoF, low-frequency; MAF, minor allele frequency; SD, standard deviation; SKAT-O, an optimal sequence kernel association test; SNP, single nucleotide polymorphism; TG, triglycerides; YRI, Yoruba people of Ibadan from Nigeria

The values of each lipid phenotype outside the mean ± 3.5 standard deviation (SD) were excluded from downstream gene-based, single-site, and haplotype analyses. However, the extreme phenotypic values associated with rare variants (MAF ≤1 %) were maintained during rare variant analysis, as was the case for the p70201/chr12:125279319 variant (see study workflow in Fig. 1). Values of the five lipid and apolipoprotein traits—HDL-C, LDL-C, TG, ApoA-I, and ApoB—were transformed using the Box-Cox transformation. For each trait, we used stepwise regression method to select the most parsimonious set of covariates from the following list: sex, age, body mass index, waist, current smoking (yes/no), minutes of walking or biking to work each day (jobmin), and occupational status (staff: junior [non-professional staff]/senior [professional and administrative staff]). Genetic association analyses, including gene-based, single-site, LoF/rare variant, and haplotype association tests, were performed using linear regression models that included significant covariates for each variable (Additional file 13: Table S8).

The gene-based association analysis was conducted under linear additive model for the combined evaluation of common and LoF/rare variants (*n* = 136, excluding p70201/chr12:125279319; see details above in paragraph two of this section) for five major lipid traits using the versatile gene-based association study [VEGAS] (http://gump.qimr.edu.au/VEGAS/) software [44]. The significance threshold for the gene-based test was set at *P*-value of 0.05.

Following gene-based analysis, which primarily implicated *SCARB1* in regulation of HDL-C and ApoA-I levels, we further elucidated the association of *SCARB1* variants with these two traits using additional tests. In single-site association analysis, *P*-values for each trait were adjusted for multiple testing using Benjamini-Hochberg procedure [45] to determine the false discovery rate [FDR] (*q*-value). For common variants (MAF ≥5 %), a nominal *P*-value of <0.05 was considered to be suggestive evidence of association, and an FDR cut-off of 0.20 was used to define statistical significance. For LoF/rare variants (MAF <5 %), the single-site association results were interpreted separately because of inadequate power of our study to detect individual statistical significance for these variants.

We conducted an optimal sequence kernel association test (SKAT-O) [46] to evaluate the association between a total of 43 LoF/rare variants (MAF <5 %) and the two lipid traits (HDL-C and ApoA-I) by using three different MAF thresholds: <5 % (*n* = 43), ≤2 % (*n* = 26), and ≤1 % (*n* = 23). A significant SKAT-O test was set at a *P*-value of <0.05.

Haplotype association analysis was performed using the generalized linear model. We applied a fixed sliding window approach that included four variants per window and sliding for one variant at a time. For each window, a global *P*-value was used to assess the association between the haplotypes with frequency >1 % and a given trait. A global *P*-value threshold of 0.05 was used to define significant haplotype association.

All analyses, except for VEGAS, were performed using the R statistical software (http://www.r-project.org/) and relevant R packages (i.e., Haplo.Stats for haplotype analysis and SKAT for SKAT-O analysis).

## Results

### Identification and distribution of *SCARB1* sequence variants in 95 individuals with extreme HDL-C levels

Resequencing of *SCARB1* exons and exon-intron boundaries plus flanking regions in 95 African Blacks with extreme HDL-C levels identified 83 variants, of which 51 had MAF <5 % (Additional file 3: Table S3 and Additional file 5: Figure S1). The majority of 83 variants (*n* = 73) were previously identified (dbSNP build 139: GRCh37.p10). Most variants (*n* = 80) were singlenucleotide variations [SNVs] (67 transitions and 13 transversions); the rest (*n* = 3) were short insertion and deletion variations (indels).

Tagger analysis using an *r*^*2*^ cutoff of 0.9 identified 28 bins for 32 common variants (MAF ≥5 %), of which three included more than one variant (*r*^2^ ranging from 0.95 to 1.0) (Additional file 6: Figure S2). One of these three bins contained two variants (rs204901986 and rs34339961) in complete LD (*r*^2^ = 1.0). Of 51 LoF/rare variants (MAF between 1 and 5 %, *n* = 31; MAF ≤1 %, *n* = 20), 17 were present only in the high HDL-C group (MAF ranging between 0.010 and 0.042) and eight were observed only in the low HDL-C group (MAF ranging between 0.011 and 0.033). In the high HDL-C group, 29 of 48 (~60 %) individuals cumulatively carried at least one LoF/rare variant, ranging from 1 to 7 variants. Similarly, in the low HDL-C group, 27 of 47 (~57 %) individuals carried at least one LoF/rare variant, ranging from 1 to 9 variants.

Most variants (*n* = 60) from our sequencing were located in intronic regions, of which two (rs113910315, MAF = 0.005 and rs10396210, MAF = 0.138) were within splice sites (defined as ± 20 bp from the start or end of an exon). The former splice site variant was observed only in the low HDL-C group.

Of the total eight coding variants observed, four were common variants (rs2070242 [p.Ser4Ser], rs10396208 [p.Cys21Cys], and rs5888 [p.Ala350Ala], and rs701103 [p.Gly499Arg]—3′ untranslated region [UTR] in isoform 1 and exon 13 in isoform 2), and the remaining four were LoF/rare variants (rs4238001 [p.Gly2Ser], rs5891 [p.Val135Ile], rs5892 [p.Phe301Phe], and rs141545424 [p.Gly501Gly]). Of note, two LoF/rare coding variants, (rs5891 [p.Val135Ile] and rs141545424 [p.Gly501Gly]), were found only in the high HDL-C group.

Fifteen variants were located in either UTRs (*n* = 5) or flanking regions (*n* = 10). One 3′ UTR variant (rs150512235, MAF = 0.006) was very close to a predicted microRNA-145 (miR-145) target site (TargetScanHuman version 6.2, http://www.targetscan.org/). One 5′ flanking variant (rs181338950, MAF = 0.048) was located in the putative promoter region [47].

All 10 novel variants (9 SNVs and 1 insertion) identified in this study have been submitted to dbSNP database ([batch ID: SCARB1_AB]:

http://www.ncbi.nlm.nih.gov/SNP/snp_viewTable.cgi?handle=KAMBOH) and were non-coding with MAF <5 % (ranging between 0.005 and 0.011; Additional file 4: Table S4). Of these novel variants, six and four were present only in the high and low HDL-C groups, respectively.

### Genotyping of *SCARB1* variants in the entire sample of 788 individuals

Since our sequencing was focused primarily on coding regions, we selected additional HapMap tagSNPs from the HapMap-YRI data in order to cover the entire *SCARB1* gene for common genetic variation in *SCARB1*. Altogether we selected 149 variants for genotyping in our entire African Black sample as follows: 78 variants (28 common variants and 50 LoF/rare variants) discovered in the sequencing step (Additional file 3: Table S3, Additional file 5: Figure S1, and Additional file 6: Figure S2), 69 common HapMap-YRI tagSNPs (Additional file 7: Table S5), and two additional variants with reported association in the literature (Additional file 9: Table S6).

Of these 149 variants, 11 (10 from sequencing, including one promoter [rs181338950], one coding (rs4238001 [p.Gly2Ser]), and one novel [p87459/chr12:125262061], and 1 from HapMap tagSNPs [rs4765180]) failed genotyping, and one (rs866793 from HapMap tagSNPs) failed QC measures. Thus, a total of 137 variants (Additional file 9: Table S6 and Additional file 11: Figure S4) that passed QC were advanced into association analyses with five lipoprotein-lipid traits.

The majority of 137 genotyped variants (*n* = 120) were located in introns, 11 were in exons, and six were in 3′ flanking region (Table 2 and Additional file 12: Figure S5). Ninety-four of 137 variants had MAF ≥5 %, including four coding variants, one UTR variant, two deletions, and one splice site variant. The remaining 43 variants had MAF <5 % (MAF between 1 and 5 %, *n* = 20; MAF ≤1 %, *n* = 23), including three coding variants, three UTR variants, one insertion, and one splice variant.

**Table 2:** Distribution of 137 *SCARB1* genotyped variants

	Total	MAF ≥5 %	MAF between 1-5 %	MAF ≤1 %
	N (%)	n (%)	n (%)	n (%)
Total variants	137 (100.00)	94 (68.61)	20 (14.60)	23 (16.79)
By known/novel^a^				
Known	128 (93.43)	94 (68.61)	20 (14.60)	14 (10.22)
Single-nucleotide variation	126	92	20	14
Short indels	2	2		
Novel	9 (6.57)			9 (6.57)
Single-nucleotide variation	8			8
Short indels	1			1
By location				
Exons-coding^c^	7	4^c^	1	2
Exons-UTRs	4	1	1	2
Introns	118	85	16	17
Introns-splice sites^b^	2	1		1
3′ flanking	6	3	2	1
By amino acid change				
Non-synonymous^c^	2	1^c^		1
Synonymous	5	3	1	1

*Indels* insertion and deletion variations, *MAF* minor allele frequency, *UTR* untranslated region

The list of 137 genotyped variants is shown in Additional file 9: Table S6

The list of 10 novel variants is shown in Additional file 4: Table S4

^a^dbSNP build 139: GRCh37.p10. All 10 novel variants identified in this study have been submitted to dbSNP (batch ID: SCARB1_AB): http://www.ncbi.nlm.nih.gov/SNP/snp_viewTable.cgi?handle=KAMBOH

^b^Splice site, defined as ± 20 bp from the start or end of an exon

^c^Including rs701103 (p.Gly499Arg; MAF = 0.2451) that is located in exon 13-3′ UTR and translated only in isoform 2

Of the 10 novel variants discovered in the sequencing step, nine (8 SNVs and 1 insertion) with MAF <1 % were successfully genotyped (Additional file 4: Table S4). There was one individual with plasma HDL-C levels above the mean + 3.5 SD carrying one novel variant—p70201/chr12:125279319 (MAF = 0.0010). Although this extreme HDL-C value was excluded as outlier from the gene-based, single-site, and haplotype analyses, it was included in the SKAT-O rare variant analysis considering a possible large effect size of this variant (Fig. 1).

### Gene-based association analyses

Gene-based tests revealed a nominally significant association (*P* = 0.0421; Table 3) of *SCARB1* variants with HDL-C levels (best SNP: rs141545424 [p.Gly501Gly], exon 12, MAF = 0.0007, *P* = 0.0016). Additionally, a trend for association (*P* = 0.1016) was also observed for ApoA-I levels (best SNP: rs7134858, intron 6, MAF = 0.1560, *P* = 0.0052).

**Table 3:** Gene-based association analysis results

Trait	Variants	Test Statistics	*P*	Best SNP		
	(n)			SNP Name^a^-SNP ID^b^	MAF	*P*
HDL-C	136	207.5483	**0.0421**	p82264-rs141545424	0.0007	0.0016
LDL-C	136	134.1860	0.4640	p32777-rs11057841	0.2805	0.0047
TG	136	118.1598	0.6700	p86316-rs701104	0.0487	0.0357
ApoA-I	136	183.5565	0.1016	p55963-rs7134858	0.1560	0.0052
ApoB	136	143.7284	0.3760	p22116-rs12370382	0.0645	0.0153

*ApoA-I* apolipoprotein A-I, *ApoB* apolipoprotein B, *HDL-C* high-density lipoprotein cholesterol, *LDL-C* low-density lipoprotein cholesterol, *MAF* minor allele frequency, *SNP* single nucleotide polymorphism, *TG* triglycerides

All results were adjusted for covariates: sex, age, body mass index, waist, current smoking (yes/no), minutes of walking or biking to work each day (jobmin), and occupational status [staff: junior (non-professional staff)/senior (professional and administrative staff)]

Nominally significant gene-based *P*-values (*P* < 0.05) are shown in **bold**

^a^RefSeq of *SCARB1*: hg19, NM_005505 (CHIP Bioinformatics)

^b^dbSNP build 139: GRCh37.p10

Since the gene-based tests showed evidence of associations with HDL-C and ApoA-I, we primarily focused on these two traits to further examine the *SCARB1* variants in the entire sample of 788 African Blacks.

### Single-site association analyses of common *SCARB1* variants

Of 94 common *SCARB1* variants with MAF ≥5 %, 10 showed nominal associations (*P* < 0.05) with HDL-C and/or ApoA-I (Table 4; see results for each trait in Additional file 14: Table S9 and Additional file 15: Table S10), of which three (rs11057851, rs4765615, and rs838895) exhibited associations with both HDL-C and ApoA-I.

**Table 4:** Nominally significant single-site associations (*P* < 0.05) of common *SCARB1* variants

SNP Name^a^	SNP ID^b^	Chr12 Position^c^	Location	Amino Acid Change	RegDB Score^d^	Major/Minor Alleles	MAF	β	SE	R^2^ (%)	*P*	FDR	Secondary Trait (Effect)	Top 3 Variants
HDL-C														
p20207	rs11057853	125329313	Intron 1		5	G/A	0.4484	0.4082	0.1925	1.0650	0.0343	0.4235		
p20741	rs11057851	125328779	Intron 1		5	C/T	0.3237	−0.5924	0.2067	1.3010	**0.0043**	**0.1465**	ApoA-I (↓)	Top 1
p45516	rs1902569	125304004	Intron 1		5	G/A	0.1544	0.5447	0.2629	0.6390	0.0386	0.4375		
p49690	rs4765615	125299830	Intron 2		5	G/A	0.4426	−0.4646	0.1866	0.9330	0.0130	0.2526	ApoA-I (↓)	
p79828	rs838895	125269692	Intron 11		5	C/G	0.3171	0.4961	0.2059	0.8220	0.0162	0.2756	ApoA-I (↑)	
ApoA-I														
p20741	rs11057851	125328779	Intron 1		5	C/T	0.3237	−1.2331	0.5117	0.8600	0.0162	0.3186	HDL-C (↓)	
p49690	rs4765615	125299830	Intron 2		5	G/A	0.4426	−0.9139	0.4614	0.6770	0.0480	0.5022	HDL-C (↓)	
p55963	rs7134858	125293557	Intron 6		6	C/T	0.1560	1.7537	0.6260	1.0710	**0.0052**	0.2918		Top 2
p63483	rs838912	125286037	Intron 7		7	G/A	0.0867	1.8700	0.8230	0.6880	0.0234	0.3972		
p64772	rs5888	125284748	Exon 8	Ala350Ala	3a	C/T	0.0961	2.0962	0.7888	0.9460	0.0080	0.2918		Top 3
p79721	rs838896	125269799	Intron 11		5	G/C	0.3104	1.1147	0.5056	0.7270	0.0278	0.4197		
p79828	rs838895	125269692	Intron 11		5	C/G	0.3171	1.2206	0.5074	0.7800	0.0164	0.3186	HDL-C (↑)	
p83884	rs701106	125265636	Intron 12		5	C/T	0.2597	1.2967	0.5352	0.7770	0.0156	0.3186		

*ApoA-I* apolipoprotein A-I, *FDR* false discovery rate, *HDL-C* high-density lipoprotein cholesterol, *MAF* minor allele frequency, *RegDB* RegulomeDB, *SE* standard error, *SNP* single nucleotide polymorphism, *UTR* untranslated region, R^*2*^, the proportion of the phenotypic variance explained by the variant; ↓, decreased; ↑, increased

Alleles on reverse strand. HDL-C and ApoA-I variables were in mg/dL and Box-Cox transformed

Results were adjusted for covariates: sex, age, waist, current smoking (yes/no), and minutes of walking or biking to work each day (jobmin) for HDL-C; sex and age for ApoA-I

**Fig. 2:**
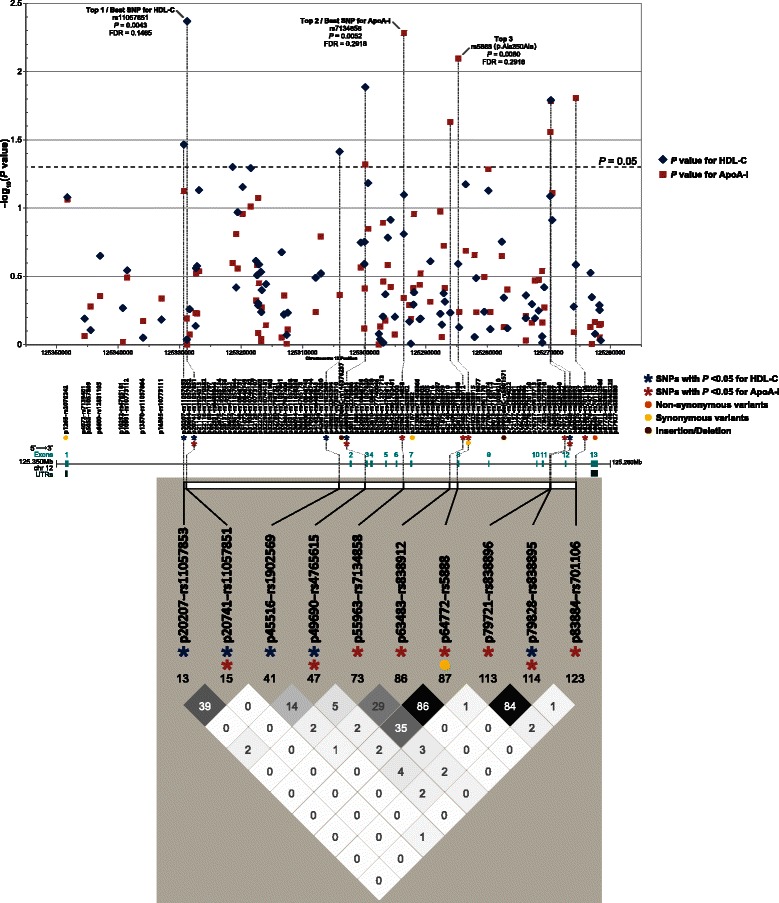
Single-site *P*-values of 94 *SCARB1* common variants for HDL-C and ApoA-I. *Top*: The -log_10_ *P*-values are presented in the Y-axis. A total of 94 genotyped variants with MAF ≥5 % are shown on *SCARB1* gene (5′ → 3′; RefSeq: hg19, NM_005505) in the X-axis. The dash line indicates the nominal significance threshold (*P* = 0.05). *Middle*: Gene structure of *SCARB1. Bottom*: Linkage disequilibrium (LD) plot of 10 *SCARB1* variants with *P*-values <0.05. Shades and values (*r*^*2*^ × 100) in each square of LD plot indicate pairwise correlations: black indicating *r*^2^ = 1, white indicating *r*^2^ = 0, and shade intensity indicating *r*^2^ between 0 and 1. Marker names are shown as “SNP name-SNP ID”. SNP ID is based on dbSNP build 139. ApoA-I, apolipoprotein A-I; FDR, false discovery rate; HDL-C, high-density lipoprotein cholesterol; LD, linkage disequilibrium; MAF, minor allele frequency; SNP, single nucleotide polymorphism; UTR, untranslated region

The most significant *P*-value for each trait is shown in **bold**, see the single-site association (−log_10_ *P*) plot and pairwise correlations (*r*^*2*^) in Fig. 2

FDR that reached a threshold of <0.20 is shown in **bold**

^a, c^RefSeq of *SCARB1*: hg19, NM_005505 (CHIP Bioinformatics)

^b^dbSNP build 139: GRCh37.p10

^d^Detailed RegulomeDB (version 1.0) scoring scheme is described in Additional file 17: Table S12 or at http://regulome.stanford.edu/help, see functional assignments in Additional file 18: Table S13

The most significant association was found between rs11057851 and HDL-C (β = −0.5924, *P* = 0.0043, FDR = 0.1465). The second best association was between rs7134858 and ApoA-I (β = 1.7537, *P* = 0.0052, FDR = 0.2918), followed by the association of rs5888 (p.Ala350Ala) with ApoA-I (β = 2.0962, *P* = 0.0080, FDR = 0.2918).

Of 10 variants that showed nominal associations, high LD (*r*^2^ > 0.80) was observed for two pairs of variants (Fig. 2), between rs8388912 and rs5888 (p.Ala350Ala; *r*^2^ = 0.86), and between rs838896 and rs838895 (*r*^2^ = 0.84).

### Association analyses of low-frequency/rare *SCARB1* variants

The LoF/rare variants (*n* = 43) were categorized into three groups based on their frequencies for association analysis with HDL-C and ApoA-I using SKAT-O: MAF <5 % (*n* = 43), MAF ≤2 % (*n* = 26), and MAF ≤1 % (*n* = 23). Although no association between LoF/rare variants and ApoA-I was detected, the group of 23 variants with MAF ≤1 % yielded nominal association with HDL-C levels (*P* = 0.0478; Table 5).

**Table 5:** Association results for low-frequency and rare *SCARB1* variants (MAF <5 %)

MAF	No of Variants	No of Samples with/without Variants	HDL-C		ApoA-I	
			Stat	*P*	Stat	*P*
**≤0.01**	23^a^	93/694	126653.8207	**0.0478**	60151.0985	0.3707
**≤0.02**	26	134/653	123009.0805	0.1324	48439.6697	0.5166
**<0.05**	43	442/346	135697.1974	0.0737	298813.0544	0.1517

*ApoA-I* apolipoprotein A-I, *HDL-C* high-density lipoprotein cholesterol, *MAF* minor allele frequency, *SD* standard deviation, *SNP* single nucleotide polymorphism

Results were adjusted for covariates: sex, age, waist, current smoking (yes/no), and minutes of walking or biking to work each day (jobmin) for HDL-C; sex and age for ApoA-I

Nominally significant *P*-values (*P* < 0.05) are shown in **bold**

^a^Including p70201/chr12:125279319 that was observed in one individual with an outlier value (above the mean + 3.5 SD). See details in Result Section 3.5

We then individually examined the association of 23 variants with MAF ≤1 % with HDL-C and ApoA-I. Six of these rare variants showed association with either HDL-C levels or both HDL-C and ApoA-I levels (Table 6). While three of them are known variants (rs115604379, rs377124254, and rs141545424 [p.Gly501Gly]), the other three are novel (p52919/chr12:125296601, p54611/chr12:125294909, and p54856/chr12:125294664). Moreover, four of these six rare variants (rs377124254, rs141545424 [p.Gly501Gly], p54611/chr12:125294909, and p54856/chr12:125294664) were present in individuals with extreme phenotypic values (above or below the 3rd percentile). Two of these variants (rs377124254: β = 11.5518, *P* = 0.0016; rs141545424 [p.Gly501Gly]: β = 11.585, *P* = 0.0016) were found in a single subject who had very high HDL-C level. Whereas the other two were observed in one individual each, who had extremely low HDL-C levels (p54611/chr12:125294909: β = −9.5243, *P* = 0.0097; p54856/chr12:125294664: β = −8.4305, *P* = 0.0215) and ApoA-I levels (p54611/chr12:125294909: β = −19.3821, *P* = 0.0344; p54856/chr12:125294664: β = −24.0757, *P* = 0.0082). This rare variant group also included a novel variant (p70201/chr12:125279319) that was observed in one individual with an unusually high plasma HDL-C level (above the mean + 3.5 SD).

**Table 6:** Characteristics and effects of 6 *SCARB1* rare variants of interest

SNP Name^a^	SNP ID^b^	Chr12 Position^c^	Location	Amino Acid Change	RegDB Score^d^	Major/Minor Alleles	MAF	GT	GT Count (Carrier Freq)	Adjusted Mean ± SD (mg/dL)	β	SE	R^2^ (%)	*P*	FDR	Second Assoc Trait (Effect)
HDL-C																
p52919		125296601	Intron 4		5	G/T	0.0013	GG	734	47.87 ± 12.71	−7.4063	2.5863	1.1050	0.0043	0.1465	ApoA-I (↓)
								GT	2 (0.27)	24.67 ± 9.26						
p53372	rs115604379	125296148	Intron 5		5	C/T	0.0066	CC	729	47.68 ± 12.64	3.0372	1.1642	0.9140	0.0093	0.2190	
								CT	10 (1.35)	58.2 ± 13.03						
p54611		125294909	Intron 5		4	T/C	0.0007	TT	742	47.86 ± 12.68	−9.5243	3.6710	0.8920	0.0097	0.2190	ApoA-I (↓)
								TC	1 (0.13)	19.59 ± NA						
p54856		125294664	Intron 6		4	C/T	0.0007	CC	742	47.85 ± 12.70	−8.4305	3.6579	0.7130	0.0215	0.3243	ApoA-I (↓)
								CT	1 (0.13)	21.48 ± NA						
p77620	rs377124254	125271900	Intron 10		5	G/A	0.0007	GG	735	47.77 ± 12.67	11.5518	3.6514	1.3500	0.0016	0.1104	
								GA	1 (0.14)	90.2 ± NA						
p82264	rs141545424	125267256	Exon 12	Gly501Gly	5	C/A	0.0007	CC	739	47.77 ± 12.66	11.5850	3.6469	1.3530	0.0016	0.1104	
								CA	1 (0.14)	90.31 ± NA						
ApoA-I																
p52919		125296601	Intron 4		5	G/T	0.0013	GG	741	136.81 ± 27.74	−13.4137	6.4689	0.5750	0.0385	0.4359	HDL-C (↓)
								GT	2 (0.27)	97.42 ± 18.38						
p54611		125294909	Intron 5		4	T/C	0.0007	TT	748	136.83 ± 27.66	−19.2831	9.0970	0.5980	0.0344	0.4359	HDL-C (↓)
								TC	1 (0.13)	80.62 ± NA						
p54856		125294664	Intron 6		4	C/T	0.0007	CC	748	136.87 ± 27.61	−24.0757	9.0781	0.9330	0.0082	0.2918	HDL-C (↓)
								CT	1 (0.13)	67.98 ± NA						

*ApoA-I* apolipoprotein A-I, *FDR* false discovery rate, *GT* genotype, *HDL-C* high-density lipoprotein cholesterol, *MAF* minor allele frequency, *RegDB* RegulomeDB, *SD* standard deviation, *SE* standard error, *SNP* single nucleotide polymorphism; R^***2***^, the proportion of the phenotypic variance explained by the variant; ↓, decreased

All alleles were on reverse stand. HDL-C and ApoA-I variables were in mg/dL and Box-Cox transformed

Results were adjusted for covariates: sex, age, waist, current smoking (yes/no), and minutes of walking or biking to work each day (jobmin) for HDL-C; sex and age for ApoA-I.

Detailed single-site association results are shown in Additional file 14: Table S9 and Additional file 15: Table S10.

^a, c^RefSeq of *SCARB1*: hg19, NM_005505 (CHIP Bioinformatics)

^b^dbSNP build 139: GRCh37.p10. All 10 novel variants identified in this study have been submitted to dbSNP (batch ID: SCARB1_AB): http://www.ncbi.nlm.nih.gov/SNP/snp_viewTable.cgi?handle=KAMBOH

^d^The RegulomeDB (version 1.0) scoring scheme and functional assignments are described in Additional file 17: Table S12 and Additional file 18: Table S13, respectively

### Haplotype association analyses

The 4-SNP sliding window haplotype analyses revealed associations of 32 haplotype windows with HDL-C and/or ApoA-I (global *P* < 0.05; Table 7; see results for each trait in Additional file 16: Table S11), of which five (windows #47, #72, #111, #112, and #123) were associated with both.

**Table 7:** Significant haplotype association (global *P* < 0.05) of 136 *SCARB1* genotyped variants with HDL-C and ApoA-I

Wind #	SNP 1 - SNP 4	Chr12 Position^c^	Location	Amino Acid Change	Major/ Minor Alleles	MAF	β	Single-site *P*	Haplotype #	Hap Seq	Hap Freq	Coef	SE	t.stat	Hap *P*	Global *P*
	(SNP Name^a^-SNP ID^b^/Chr12 Pos^c^)															
HDL-C																
**39**	p41632-rs6488943	125307888	Intron 1		A/C	0.2954	−0.2195	0.3244	**h39.1**	CCGG	0.0315	0.4305	0.6471	0.6654	0.5060	0.0207
**39**	p42467-rs11057830	125307053	Intron 1		C/T	0.1523	−0.2810	0.3015	**h39.2**	CCGA	0.2508	−0.5918	0.2725	−2.1713	0.0302	
**39**	p45516-rs1902569	125304004	Intron 1		G/A	0.1544	0.5447	**0.0386**	**h39.3**	ATGA	0.1414	−0.6841	0.3192	−2.1433	0.0324	
**39**	p45627-rs12297372	125303893	Intron 1		A/G	0.0487	−0.0483	0.9156	**h39.4**	ACAA	0.1514	0.1991	0.2963	0.6720	0.5018	
									**h39.5**	ACGG	0.0155	−1.7144	0.9080	−1.8880	0.0594	
									**h39.6 (rare)**	****	0.0148	2.5239	1.0902	2.3151	0.0209	
									**hap.base39**	ACGA	0.3946	NA	NA	NA	NA	
**44**	p48969-rs2343394	125300551	Intron 2		C/T	0.1898	0.3165	0.1788	**h44.1**	TCWG	0.1855	0.5292	0.2523	2.0977	0.0363	0.0271
**44**	p49537-rs7305310	125299983	Intron 2		C/T	0.1007	−0.3396	0.2566	**h44.2**	CCDG	0.2244	0.4676	0.2429	1.9249	0.0546	
**44**	p49570delC-rs145376237	125299950	Intron 2		W/D	0.2276	0.3121	0.1773	**h44.3**	CCWG	0.0446	1.0491	0.4882	2.1489	0.0320	
**44**	p49690-rs4765615	125299830	Intron 2		G/A	0.4426	−0.4646	**0.0130**	**h44.4**	CTWG	0.1018	−0.1197	0.3121	−0.3835	0.7015	
									**h44.5 (rare)**	****	0.0089	−0.9887	1.0998	−0.8990	0.3689	
									**hap.base44**	CCWA	0.4348	NA	NA	NA	NA	
**45**	p49537-rs7305310	125299983	Intron 2		C/T	0.1007	−0.3396	0.2566	**h45.1**	CDGC	0.2282	0.4661	0.2393	1.9473	0.0519	0.0155
**45**	p49570delC-rs145376237	125299950	Intron 2		W/D	0.2276	0.3121	0.1773	**h45.2**	CWGC	0.2302	0.6926	0.2376	2.9146	0.0037	
**45**	p49690-rs4765615	125299830	Intron 2		G/A	0.4426	−0.4646	**0.0130**	**h45.3**	TWGC	0.1020	−0.0653	0.3085	−0.2115	0.8325	
**45**	p49759-rs146272788	125299761	Intron 2		C/T	0.0020	2.5988	0.2219	**h45.4 (rare)**	****	0.0030	2.0667	2.0848	0.9913	0.3219	
									**hap.base45**	CWAC	0.4366	NA	NA	NA	NA	
**46**	p49570delC-rs145376237	125299950	Intron 2		W/D	0.2276	0.3121	0.1773	**h46.1**	DGCG	0.2228	0.4373	0.2413	1.8123	0.0703	0.0278
**46**	p49690-rs4765615	125299830	Intron 2		G/A	0.4426	−0.4646	**0.0130**	**h46.2**	WGCG	0.3311	0.4910	0.2105	2.3326	0.0199	
**46**	p49759-rs146272788	125299761	Intron 2		C/T	0.0020	2.5988	0.2219	**h46.3 (rare)**	****	0.0080	1.9089	1.0569	1.8061	0.0713	
**46**	p49978-rs5891	125299542	Exon 3	Val135lle	G/A	0.0058	1.3374	0.2791	**hap.base46**	WACG	0.4381	NA	NA	NA	NA	
**47**	p49690-rs4765615	125299830	Intron 2		G/A	0.4426	−0.4646	**0.0130**	**h47.1**	ACGG	0.4346	−0.4701	0.1824	−2.5777	0.0101	0.0079
**47**	p49759-rs146272788	125299761	Intron 2		C/T	0.0020	2.5988	0.2219	**h47.2 (rare)**	****	0.0101	1.4683	0.9441	1.5552	0.1203	
**47**	p49978-rs5891	125299542	Exon 3	Val135lle	G/A	0.0058	1.3374	0.2791	**hap.base47**	GCGG	0.5553	NA	NA	NA	NA	
**47**	p50024-rs368880622	125299496	Intron 3		G/T	0.0026	1.6506	0.4362								
**63**	p53359-rs112371713	125296161	Intron 5		G/A	0.1243	0.4193	0.1651	**h63.1**	ACGA	0.1237	0.3273	0.3011	1.0871	0.2773	0.0394
**63**	p53372-rs115604379	125296148	Intron 5		C/T	0.0066	3.0372	0.0093	**h63.2**	GCGG	0.0427	−0.1630	0.4738	−0.3441	0.7309	
**63**	p53790-rs4765614	125295730	Intron 5		G/A	0.2653	−0.3281	0.1218	**h63.3**	GCAA	0.2678	−0.2408	0.2194	−1.0975	0.2728	
**63**	p54445-rs60910935	125295075	Intron 5		A/G	0.0418	−0.1247	0.7963	**h63.4 (rare)**	****	0.0068	2.9428	1.2559	2.3432	0.0194	
									**hap.base63**	GCGA	0.5591	NA	NA	NA	NA	
**72**	p55923-rs838900	125293597	Intron 6		G/A	0.3921	0.2787	0.1549	**h72.1**	ACAG	0.2725	0.4039	0.2520	1.6024	0.1095	0.0315
**72**	p55963-rs7134858	125293557	Intron 6		C/T	0.1560	0.4418	0.0799	**h72.2**	ACGG	0.1086	−0.1763	0.3929	−0.4486	0.6538	
**72**	p56845-rs838902	125292675	Intron 6		A/G	0.4249	−0.0786	0.6801	**h72.3**	GTAG	0.1284	0.3877	0.3170	1.2228	0.2218	
**72**	p57004-rs187562853	125292516	Intron 6		G/A	0.0098	1.6474	0.0872	**h72.4**	GTGG	0.0297	0.8722	0.6546	1.3323	0.1832	
									**h72.5**	GCAG	0.1716	−0.4913	0.3344	−1.4690	0.1422	
									**h72.6 (rare)**	****	0.0101	1.7731	0.9506	1.8653	0.0625	
									**hap.base72**	GCGG	0.2791	NA	NA	NA	NA	
**111**	p78747-rs2293440	125270773	Intron 11		T/C	0.4112	−0.1684	0.3806	**h111.1**	CCCG	0.0306	0.7458	0.5599	1.3321	0.1832	0.0040
**111**	p78791-rs75289200	125270729	Intron 11		T/C	0.0321	0.7037	0.2078	**h111.2**	CTGC	0.1534	−0.5556	0.2830	−1.9629	0.0500	
**111**	p79721-rs838896	125269799	Intron 11		G/C	0.3104	0.3565	0.0817	**h111.3**	CTCG	0.2269	0.1234	0.2391	0.5162	0.6058	
**111**	p79828-rs838895	125269692	Intron 11		C/G	0.3171	0.4961	**0.0162**	**h111.4**	TTGG	0.0180	2.3022	0.7617	3.0225	0.0026	
									**h111.5**	TTCG	0.0439	0.5755	0.5317	1.0823	0.2795	
									**h111.6**	TTCC	0.0145	0.9606	0.8068	1.1907	0.2342	
									**h111.7 (rare)**	****	0.0033	0.7755	2.1917	0.3538	0.7236	
									**hap.base111**	TTGC	0.5094	NA	NA	NA	NA	
**112**	p78791-rs75289200	125270729	Intron 11		T/C	0.0321	0.7037	0.2078	**h112.1**	CCGA	0.0311	0.7440	0.5559	1.3384	0.1812	0.0055
**112**	p79721-rs838896	125269799	Intron 11		G/C	0.3104	0.3565	0.0817	**h112.2**	TGGA	0.0171	2.3734	0.7506	3.1621	0.0016	
**112**	p79828-rs838895	125269692	Intron 11		C/G	0.3171	0.4961	**0.0162**	**h112.3**	TGCA	0.0112	−1.2672	0.9074	−1.3964	0.1630	
**112**	p80045-rs838893	125269475	Intron 11		G/A	0.3244	0.3127	0.1224	**h112.4**	TCGA	0.2704	0.2488	0.2164	1.1501	0.2505	
									**h112.5**	TCCG	0.0139	1.1219	0.8186	1.3704	0.1710	
									**h112.6 (rare)**	****	0.0068	1.6244	1.2691	1.2800	0.2009	
									**hap.base112**	TGCG	0.6493	NA	NA	NA	NA	
**113**	p79721-rs838896	125269799	Intron 11		G/C	0.3104	0.3565	0.0817	**h113.1**	GGAG	0.0171	2.3949	0.7509	3.1895	0.0015	0.0048
**113**	p79828-rs838895	125269692	Intron 11		C/G	0.3171	0.4961	**0.0162**	**h113.2**	GCAG	0.0120	−1.1963	0.8784	−1.3619	0.1736	
**113**	p80045-rs838893	125269475	Intron 11		G/A	0.3244	0.3127	0.1224	**h113.3**	CGAG	0.2996	0.3071	0.2067	1.4861	0.1377	
**113**	p81863-rs185445624	125267657	Intron 11		G/A	0.0020	−0.9612	0.6510	**h113.4**	CCGG	0.0139	1.1509	0.8168	1.4090	0.1592	
									**h113.5 (rare)**	****	0.0081	1.1622	1.0896	1.0666	0.2865	
									**hap.base113**	GCGG	0.6493	NA	NA	NA	NA	
**114**	p79828-rs838895	125269692	Intron 11		C/G	0.3171	0.4961	**0.0162**	**h114.1**	GAGC	0.3173	0.3755	0.2023	1.8559	0.0639	0.0447
**114**	p80045-rs838893	125269475	Intron 11		G/A	0.3244	0.3127	0.1224	**h114.2**	CGGT	0.0306	−0.8840	0.5344	−1.6541	0.0985	
**114**	p81863-rs185445624	125267657	Intron 11		G/A	0.0020	−0.9612	0.6510	**h114.3**	CAGC	0.0111	−1.2612	0.9170	−1.3754	0.1694	
**114**	p82019-rs838890	125267501	Intron 11		C/T	0.0320	−1.0051	0.0618	**h114.4 (rare)**	****	0.0086	0.9073	1.0936	0.8296	0.4070	
									**hap.base114**	CGGC	0.6325	NA	NA	NA	NA	
**117**	p82019-rs838890	125267501	Intron 11		C/T	0.0320	−1.0051	0.0618	**h117.1**	CCAG	0.0238	−1.0596	0.6275	−1.6884	0.0917	0.0433
**117**	p82264-rs141545424	125267256	Exon 12	Gly501Gly	C/A	0.0007	11.5850	0.0016	**h117.2**	TCGG	0.0311	−0.9657	0.5302	−1.8215	0.0689	
**117**	p82340-rs77483223	125267180	Intron 12		G/A	0.0231	−1.0458	0.1012	**h117.3 (rare)**	****	0.0067	1.6191	1.2946	1.2507	0.2114	
**117**	p82369-rs75446635	125267151	Intron 12		G/A	0.0059	0.5896	0.6322	**hap.base117**	CCGG	0.9383	NA	NA	NA	NA	
**118**	p82264-rs141545424	125267256	Exon 12	Gly501Gly	C/A	0.0007	11.5850	0.0016	**h118.1**	CAGT	0.0238	−1.0621	0.6274	−1.6929	0.0909	0.0375
**118**	p82340-rs77483223	125267180	Intron 12		G/A	0.0231	−1.0458	0.1012	**h118.2**	CGGC	0.0307	−1.0134	0.5313	−1.9073	0.0569	
**118**	p82369-rs75446635	125267151	Intron 12		G/A	0.0059	0.5896	0.6322	**h118.3 (rare)**	****	0.0067	1.6189	1.2762	1.2685	0.2050	
**118**	p82434-rs838889	125267086	Intron 12		T/C	0.0315	−1.0389	0.0526	**hap.base118**	CGGT	0.9387	NA	NA	NA	NA	
**123**	p83884-rs701106	125265636	Intron 12		C/T	0.2597	0.2471	0.2601	**h123.1**	TCCT	0.0256	−1.2114	0.6218	−1.9483	0.0518	0.0386
**123**	p86245-rs188375019	125263275	Intron 12		C/T	0.0341	0.7447	0.1639	**h123.2**	TCCG	0.2327	0.5306	0.2403	2.2085	0.0275	
**123**	p86276-rs747155	125263244	Intron 12		C/T	0.1495	0.2793	0.2980	**h123.3**	CCTG	0.1476	0.3955	0.2811	1.4071	0.1598	
**123**	p86316-rs701104	125263204	Intron 12		G/T	0.0487	−0.9838	0.0286	**h123.4**	CCCT	0.0233	−0.2329	0.7038	−0.3309	0.7408	
									**h123.5**	CTCG	0.0330	0.8888	0.5458	1.6283	0.1039	
									**h123.6 (rare)**	****	0.0029	1.1191	3.2961	0.3395	0.7343	
									**hap.base123**	CCCG	0.5348	NA	NA	NA	NA	
**124**	p86245-rs188375019	125263275	Intron 12		C/T	0.0341	0.7447	0.1639	**h124.1**	CTGA	0.1476	0.1530	0.2692	0.5683	0.5700	0.0368
**124**	p86276-rs747155	125263244	Intron 12		C/T	0.1495	0.2793	0.2980	**h124.2**	CCTG	0.0465	−1.1879	0.4699	−2.5281	0.0117	
**124**	p86316-rs701104	125263204	Intron 12		G/T	0.0487	−0.9838	0.0286	**h124.3**	CCGA	0.0915	0.1086	0.3376	0.3218	0.7477	
**124**	p86481-rs701103	125263039	Exon 13-3' UTR	Gly499Arg (isoform 2)	G/A	0.2451	0.1642	0.4492	**h124.4**	TCGG	0.0337	0.7348	0.5362	1.3702	0.1710	
									**h124.5 (rare)**	****	0.0045	4.0859	2.1131	1.9336	0.0535	
									**hap.base124**	CCGG	0.6761	NA	NA	NA	NA	
**125**	p86276-rs747155	125263244	Intron 12		C/T	0.1495	0.2793	0.2980	**h125.1**	TGAA	0.1476	0.1543	0.2689	0.5737	0.5664	0.0307
**125**	p86316-rs701104	125263204	Intron 12		G/T	0.0487	−0.9838	0.0286	**h125.2**	CTGA	0.0465	−1.1980	0.4691	−2.5535	0.0109	
**125**	p86481-rs701103	125263039	Exon 13-l3' UTR	Gly499Arg (isoform 2)	G/A	0.2451	0.1642	0.4492	**h125.3**	CGAA	0.0915	0.1139	0.3375	0.3375	0.7359	
**125**	p86967-rs187492239	125262553	Exon 13-3' UTR		A/G	0.0355	0.7743	0.1412	**h125.4**	CGGG	0.0352	0.7974	0.5241	1.5216	0.1285	
									**h125.5 (rare)**	****	0.0045	4.0989	2.1134	1.9394	0.0528	
									**hap.base125**	CGGA	0.6747	NA	NA	NA	NA	
**ApoA-I**																
**47**	p49690-rs4765615	125299830	Intron 2		G/A	0.4426	−0.9139	**0.0480**	**h47.1**	ACGG	0.4351	−0.8907	0.4584	−1.9432	0.0524	0.0343
**47**	p49759-rs146272788	125299761	Intron 2		C/T	0.0020	1.5883	0.7630	**h47.2 (rare)**	****	0.0106	3.5858	2.2998	1.5592	0.1194	
**47**	p49978-rs5891	125299542	Exon 3	Val135lle	G/A	0.0058	5.6762	0.0628	**hap.base47**	GCGG	0.5543	NA	NA	NA	NA	
**47**	p50024-rs368880622	125299496	Intron 3		G/T	0.0026	1.6012	0.7255								
**48**	p49759-rs146272788	125299761	Intron 2		C/T	0.0020	1.5883	0.7630	**h48.1**	CGGT	0.0206	3.3555	1.6564	2.0258	0.0431	0.0293
**48**	p49978-rs5891	125299542	Exon 3	Val135lle	G/A	0.0058	5.6762	0.0628	**h48.2 (rare)**	****	0.0106	4.0750	2.3644	1.7235	0.0852	
**48**	p50024-rs368880622	125299496	Intron 3		G/T	0.0026	1.6012	0.7255	**hap.base48**	CGGC	0.9688	NA	NA	NA	NA	
**48**	p50118-rs58710319	125299402	Intron 3		C/T	0.0208	3.1376	0.0571								
**49**	p49978-rs5891	125299542	Exon 3	Val135lle	G/A	0.0058	5.6762	0.0628	**h49.1**	GGTT	0.0213	3.3792	1.6416	2.0584	0.0399	0.0289
**49**	p50024-rs368880622	125299496	Intron 3		G/T	0.0026	1.6012	0.7255	**h49.2**	GGCC	0.1928	0.8864	0.5841	1.5176	0.1295	
**49**	p50118-rs58710319	125299402	Intron 3		C/T	0.0208	3.1376	0.0571	**h49.3 (rare)**	****	0.0086	4.7388	3.1873	1.4868	0.1375	
**49**	p50151-rs2278986	125299369	Intron 3		T/C	0.1933	0.8568	0.1419	**hap.base49**	GGCT	0.7774	NA	NA	NA	NA	
**70**	p54627-chr12_125294893	125294893	Intron 5		G/C	0.0020	3.6910	0.4850	**h70.1**	GCAC	0.3873	0.8579	0.5090	1.6854	0.0923	0.0140
**70**	p54856-chr12_125294664	125294664	Intron 6		C/T	0.0007	−24.0757	0.0082	**h70.2**	GCGT	0.1568	2.0940	0.6700	3.1254	0.0018	
**70**	p55923-rs838900	125293597	Intron 6		G/A	0.3921	0.3606	0.4549	**h70.3 (rare)**	****	0.0027	−2.5567	5.2200	−0.4898	0.6244	
**70**	p55963-rs7134858	125293557	Intron 6		C/T	0.1560	1.7537	**0.0052**	**hap.base70**	GCGC	0.4532	NA	NA	NA	NA	
**71**	p54856-chr12_125294664	125294664	Intron 6		C/T	0.0007	−24.0757	0.0082	**h71.1**	CACA	0.2736	0.7883	0.6210	1.2694	0.2047	0.0488
**71**	p55923-rs838900	125293597	Intron 6		G/A	0.3921	0.3606	0.4549	**h71.2**	CACG	0.1134	1.1284	0.9724	1.1604	0.2462	
**71**	p55963-rs7134858	125293557	Intron 6		C/T	0.1560	1.7537	**0.0052**	**h71.3**	CGTA	0.1296	2.1103	0.7906	2.6691	0.0078	
**71**	p56845-rs838902	125292675	Intron 6		A/G	0.4249	−0.3052	0.5129	**h71.4**	CGTG	0.0300	2.1358	1.6772	1.2734	0.2032	
									**h71.5**	CGCA	0.1706	−0.1013	0.8355	−0.1212	0.9035	
									**hap.base71**	CGCG	0.2822	NA	NA	NA	NA	
**72**	p55923-rs838900	125293597	Intron 6		G/A	0.3921	0.3606	0.4549	**h72.1**	ACAG	0.2733	0.7471	0.6218	1.2016	0.2299	0.0463
**72**	p55963-rs7134858	125293557	Intron 6		C/T	0.1560	1.7537	**0.0052**	**h72.2**	ACGG	0.1057	0.7094	0.9850	0.7202	0.4716	
**72**	p56845-rs838902	125292675	Intron 6		A/G	0.4249	−0.3052	0.5129	**h72.3**	GTAG	0.1297	2.0304	0.7898	2.5707	0.0103	
**72**	p57004-rs187562853	125292516	Intron 6		G/A	0.0098	3.2853	0.1690	**h72.4**	GTGG	0.0299	2.1741	1.6857	1.2897	0.1975	
									**h72.5**	GCAG	0.1712	−0.3122	0.8263	−0.3778	0.7057	
									**h72.6 (rare)**	****	0.0100	3.9105	2.4373	1.6044	0.1090	
									**hap.base72**	GCGG	0.2801	NA	NA	NA	NA	
**78**	p57592-rs838903	125291928	Intron 7		G/A	0.3763	−0.7661	0.1109	**h78.1**	GCAC	0.0559	1.8913	1.0469	1.8067	0.0712	0.0326
**78**	p58514-rs838905	125291006	Intron 7		T/C	0.4329	−0.4213	0.3646	**h78.2**	GTAC	0.0367	1.0784	1.2814	0.8415	0.4003	
**78**	p58664-rs865716	125290856	Intron 7		A/T	0.2708	0.5369	0.3008	**h78.3**	GTAT	0.2557	0.3365	0.6035	0.5576	0.5773	
**78**	p60255-rs3782287	125289265	Intron 7		C/T	0.2831	0.3715	0.4856	**h78.4**	GTTC	0.2463	0.4962	0.5864	0.8462	0.3977	
									**h78.5**	GTTT	0.0238	5.5715	1.6643	3.3477	0.0009	
									**h78.6 (rare)**	****	0.0075	0.6333	2.9303	0.2161	0.8289	
									**hap.base78**	ACAC	0.3740	NA	NA	NA	NA	
**79**	p58514-rs838905	125291006	Intron 7		T/C	0.4329	−0.4213	0.3646	**h79.1**	CACT	0.1270	0.3290	0.8318	0.3955	0.6926	0.0256
**79**	p58664-rs865716	125290856	Intron 7		A/T	0.2708	0.5369	0.3008	**h79.2**	TACC	0.0379	0.6384	1.2921	0.4941	0.6214	
**79**	p60255-rs3782287	125289265	Intron 7		C/T	0.2831	0.3715	0.4856	**h79.3**	TATC	0.2563	0.1851	0.6336	0.2921	0.7703	
**79**	p61872-rs838909	125287648	Intron 7		C/T	0.2199	0.9232	0.1056	**h79.4**	TTCC	0.1587	−0.6020	0.7769	−0.7749	0.4386	
									**h79.5**	TTCT	0.0880	1.8902	0.8856	2.1342	0.0331	
									**h79.6**	TTTC	0.0238	5.1755	1.6851	3.0714	0.0022	
									**h79.7 (rare)**	****	0.0059	1.2466	3.1079	0.4011	0.6885	
									**hap.base79**	CACC	0.3024	NA	NA	NA	NA	
**80**	p58664-rs865716	125290856	Intron 7		A/T	0.2708	0.5369	0.3008	**h80.1**	ACCG	0.0389	−0.3521	1.2793	−0.2753	0.7832	0.0030
**80**	p60255-rs3782287	125289265	Intron 7		C/T	0.2831	0.3715	0.4856	**h80.2**	ACTG	0.1274	−0.1816	0.7909	−0.2297	0.8184	
**80**	p61872-rs838909	125287648	Intron 7		C/T	0.2199	0.9232	0.1056	**h80.3**	ATCG	0.2611	−0.1400	0.6323	−0.2213	0.8249	
**80**	p62140-rs838910	125287380	Intron 7		G/T	0.3047	−0.0755	0.8821	**h80.4**	TCCG	0.1549	−1.3614	0.7489	−1.8178	0.0695	
									**h80.5**	TCTG	0.0901	2.0511	0.8921	2.2992	0.0218	
									**h80.6**	TTCG	0.0224	4.7307	1.8842	2.5107	0.0123	
									**h80.7 (rare)**	****	0.0083	3.1429	3.4362	0.9147	0.3607	
									**hap.base80**	ACCT	0.2970	NA	NA	NA	NA	
**81**	p60255-rs3782287	125289265	Intron 7		C/T	0.2831	0.3715	0.4856	**h81.1**	CCGC	0.1740	−1.5355	0.7276	−2.1103	0.0352	0.0050
**81**	p61872-rs838909	125287648	Intron 7		C/T	0.2199	0.9232	0.1056	**h81.2**	CCGT	0.0215	−0.5623	1.6155	−0.3481	0.7279	
**81**	p62140-rs838910	125287380	Intron 7		G/T	0.3047	−0.0755	0.8821	**h81.3**	CCTC	0.0352	3.6130	1.4518	2.4886	0.0130	
**81**	p62409-rs838911	125287111	Intron 7		C/T	0.4211	−0.6245	0.1888	**h81.4**	CCTT	0.2683	−0.7498	0.6337	−1.1832	0.2371	
									**h81.5**	CTGC	0.0886	1.4787	0.9259	1.5970	0.1107	
									**h81.6**	CTGT	0.1287	−0.2477	0.7967	−0.3109	0.7560	
									**h81.7 (rare)**	****	0.0017	4.9120	8.4190	0.5834	0.5598	
									**hap.base81**	TCGC	0.2819	NA	NA	NA	NA	
**82**	p61872-rs838909	125287648	Intron 7		C/T	0.2199	0.9232	0.1056	**h82.1**	CGTT	0.0214	0.3707	1.6055	0.2309	0.8175	0.0137
**82**	p62140-rs838910	125287380	Intron 7		G/T	0.3047	−0.0755	0.8821	**h82.2**	CTCT	0.0364	3.8641	1.3703	2.8199	0.0049	
**82**	p62409-rs838911	125287111	Intron 7		C/T	0.4211	−0.6245	0.1888	**h82.3**	CTTT	0.2692	−0.2007	0.5674	−0.3537	0.7237	
**82**	p62615-rs7138386	125286905	Intron 7		T/C	0.1137	−0.6495	0.3851	**h82.4**	TGCT	0.0869	2.1488	0.8777	2.4481	0.0146	
									**h82.5**	TGTT	0.0179	3.0085	1.9599	1.5351	0.1252	
									**h82.6**	TGTC	0.1116	−0.1961	0.7815	−0.2510	0.8019	
									**h82.7 (rare)**	****	0.0020	−4.7635	9.0097	−0.5287	0.5972	
									**hap.base82**	CGCT	0.4546	NA	NA	NA	NA	
**83**	p62140-rs838910	125287380	Intron 7		G/T	0.3047	−0.0755	0.8821	**h83.1**	GCTA	0.0854	2.0624	0.8886	2.3211	0.0205	0.0187
**83**	p62409-rs838911	125287111	Intron 7		C/T	0.4211	−0.6245	0.1888	**h83.2**	GTTG	0.0389	1.3667	1.2527	1.0910	0.2756	
**83**	p62615-rs7138386	125286905	Intron 7		T/C	0.1137	−0.6495	0.3851	**h83.3**	GTCG	0.1129	−0.3143	0.7855	−0.4002	0.6891	
**83**	p63483-rs838912	125286037	Intron 7		G/A	0.0867	1.8700	**0.0234**	**h83.4**	TCTG	0.0368	3.8488	1.3757	2.7977	0.0053	
									**h83.5**	TTTG	0.2675	−0.1681	0.5759	−0.2918	0.7705	
									**h83.6 (rare)**	****	0.0031	−0.5696	5.5038	−0.1035	0.9176	
									**hap.base83**	GCTG	0.4554	NA	NA	NA	NA	
**86**	p63483-rs838912	125286037	Intron 7		G/A	0.0867	1.8700	**0.0234**	**h86.1**	ATCG	0.0871	2.5431	0.8550	2.9743	0.0030	0.0290
**86**	p64772-rs5888	125284748	Exon 8	Ala350Ala	C/T	0.0961	2.0962	**0.0080**	**h86.2**	GCAG	0.1457	0.3613	0.6957	0.5194	0.6037	
**86**	p64923-rs838915	125284597	Intron 8		C/A	0.1435	−0.3684	0.5766	**h86.3**	GCCA	0.2814	1.0972	0.5782	1.8976	0.0581	
**86**	p65999-rs12819677	125283521	Intron 8		G/A	0.2813	0.6769	0.2052	**h86.4**	GTCG	0.0116	1.6563	2.1240	0.7798	0.4357	
									**hap.base86**	GCCG	0.4736	NA	NA	NA	NA	
**95**	p71867-rs7954022	125277653	Intron 9		C/T	0.1323	0.8502	0.2241	**h95.1**	TACT	0.1311	0.8202	0.7688	1.0669	0.2864	0.0131
**95**	p72197-rs838861	125277323	Intron 9		A/G	0.3777	−0.1507	0.7464	**h95.2**	CACC	0.0507	0.3188	1.2809	0.2489	0.8035	
**95**	p72777-rs838862	125276743	Intron 9		C/T	0.0887	0.7012	0.3938	**h95.3**	CGCT	0.1846	−0.7832	0.6960	−1.1253	0.2608	
**95**	p75766-rs838866	125273754	Intron 9		T/C	0.2116	−0.0497	0.9306	**h95.4**	CGCC	0.1022	0.7176	0.8581	0.8362	0.4033	
									**h95.5**	CGTT	0.0324	4.7525	1.5071	3.1534	0.0017	
									**h95.6**	CGTC	0.0582	−1.3987	1.0854	−1.2887	0.1979	
									**h95.7 (rare)**	****	0.0009	18.2723	NA	NA	NA	
									**hap.base95**	CACT	0.4399	NA	NA	NA	NA	
**96**	p72197-rs838861	125277323	Intron 9		A/G	0.3777	−0.1507	0.7464	**h96.1**	ACCT	0.0443	1.0796	1.2832	0.8413	0.4004	0.0484
**96**	p72777-rs838862	125276743	Intron 9		C/T	0.0887	0.7012	0.3938	**h96.2**	GCTC	0.1849	−0.7979	0.6554	−1.2176	0.2238	
**96**	p75766-rs838866	125273754	Intron 9		T/C	0.2116	−0.0497	0.9306	**h96.3**	GCCT	0.0727	−0.3866	0.9478	−0.4079	0.6835	
**96**	p75778-rs7301120	125273742	Intron 9		C/T	0.1135	0.3767	0.6174	**h96.4**	GCCC	0.0282	1.9372	1.6107	1.2027	0.2295	
									**h96.5**	GTTC	0.0319	4.2363	1.4400	2.9419	0.0034	
									**h96.6**	GTCC	0.0595	−1.3421	1.0101	−1.3286	0.1844	
									**h96.7 (rare)**	****	0.0058	−3.2342	3.8265	−0.8452	0.3983	
									**hap.base96**	ACTC	0.5728	NA	NA	NA	NA	
**97**	p72777-rs838862	125276743	Intron 9		C/T	0.0887	0.7012	0.3938	**h97.1**	CTCT	0.1997	−1.0781	0.6237	−1.7287	0.0843	0.0098
**97**	p75766-rs838866	125273754	Intron 9		T/C	0.2116	−0.0497	0.9306	**h97.2**	CCTT	0.1141	0.2005	0.7597	0.2639	0.7919	
**97**	p75778-rs7301120	125273742	Intron 9		C/T	0.1135	0.3767	0.6174	**h97.3**	CCCT	0.0336	0.7963	1.3894	0.5731	0.5667	
**97**	p76757-rs9919713	125272763	Intron 9		A/T	0.4390	−0.1860	0.6921	**h97.4**	TTCT	0.0301	4.3773	1.4494	3.0201	0.0026	
									**h97.5**	TCCT	0.0588	−1.4125	1.0117	−1.3961	0.1631	
									**h97.6 (rare)**	****	0.0050	−6.5869	3.6167	−1.8213	0.0690	
									**hap.base97**	CTCA	0.5587	NA	NA	NA	NA	
**109**	p78402-rs838898	125271118	Intron 10		G/A	0.0714	−0.9806	0.2889	**h109.1**	AGCT	0.0288	−1.4134	1.6436	−0.8600	0.3901	0.0195
**109**	p78430-rs838897	125271090	Intron 10		C/G	0.3830	−0.1887	0.6887	**h109.2**	AGTT	0.0451	−1.5093	1.2496	−1.2078	0.2275	
**109**	p78747-rs2293440	125270773	Intron 11		T/C	0.4112	−0.2984	0.5352	**h109.3**	GGCC	0.0317	3.0784	1.3763	2.2366	0.0256	
**109**	p78791-rs75289200	125270729	Intron 11		T/C	0.0321	3.6568	0.0086	**h109.4**	GGCT	0.1633	−0.4126	0.6911	−0.5971	0.5506	
									**h109.5**	GGTT	0.1088	−1.6537	0.8639	−1.9142	0.0560	
									**h109.6**	GCCT	0.1851	−1.8104	0.7168	−2.5256	0.0118	
									**hap.base109**	GCTT	0.4363	NA	NA	NA	NA	
**110**	p78430-rs838897	125271090	Intron 10		C/G	0.3830	−0.1887	0.6887	**h110.1**	GCCC	0.0305	3.0357	1.4224	2.1342	0.0331	0.0012
**110**	p78747-rs2293440	125270773	Intron 11		T/C	0.4112	−0.2984	0.5352	**h110.2**	GCTG	0.0189	−3.0973	2.2833	−1.3565	0.1753	
**110**	p78791-rs75289200	125270729	Intron 11		T/C	0.0321	3.6568	0.0086	**h110.3**	GCTC	0.1696	−0.0290	0.6830	−0.0424	0.9662	
**110**	p79721-rs838896	125269799	Intron 11		G/C	0.3104	1.1147	**0.0278**	**h110.4**	GTTG	0.1400	−2.3158	0.7741	−2.9914	0.0029	
									**h110.5**	GTTC	0.0189	1.3536	2.3385	0.5788	0.5629	
									**h110.6**	CCTG	0.1379	−2.4014	0.7888	−3.0443	0.0024	
									**h110.7**	CCTC	0.0514	−0.8677	1.2628	−0.6871	0.4922	
									**h110.8**	CTTC	0.0398	−0.1892	1.4963	−0.1264	0.8994	
									**h110.9 (rare)**	****	0.0012	7.8235	8.0313	0.9741	0.3303	
									**hap.base110**	CTTG	0.3918	NA	NA	NA	NA	
**111**	p78747-rs2293440	125270773	Intron 11		T/C	0.4112	−0.2984	0.5352	**h111.1**	CCCG	0.0305	3.5704	1.4077	2.5364	0.0114	0.0038
**111**	p78791-rs75289200	125270729	Intron 11		T/C	0.0321	3.6568	0.0086	**h111.2**	CTGC	0.1514	−2.1697	0.7058	−3.0742	0.0022	
**111**	p79721-rs838896	125269799	Intron 11		G/C	0.3104	1.1147	**0.0278**	**h111.3**	CTCG	0.2233	0.3086	0.5985	0.5157	0.6062	
**111**	p79828-rs838895	125269692	Intron 11		C/G	0.3171	1.2206	**0.0164**	**h111.4**	TTGG	0.0173	1.0502	1.9388	0.5417	0.5882	
									**h111.5**	TTGC	0.0431	0.3464	1.3140	0.2637	0.7921	
									**h111.6**	TTCC	0.0150	0.6429	1.9745	0.3256	0.7448	
									**h111.7 (rare)**	****	0.0047	3.8853	4.0634	0.9562	0.3393	
									**hap.base111**	TTGC	0.5147	NA	NA	NA	NA	
**112**	p78791-rs75289200	125270729	Intron 11		T/C	0.0321	3.6568	0.0086	**h112.1**	CCGA	0.0309	3.7315	1.3947	2.6755	0.0076	0.0412
**112**	p79721-rs838896	125269799	Intron 11		G/C	0.3104	1.1147	**0.0278**	**h112.2**	TGGA	0.0179	1.8646	1.8467	1.0097	0.3130	
**112**	p79828-rs838895	125269692	Intron 11		C/G	0.3171	1.2206	**0.0164**	**h112.3**	TGCA	0.0109	−3.3720	2.3180	−1.4547	0.1462	
**112**	p80045-rs838893	125269475	Intron 11		G/A	0.3244	0.8859	0.0774	**h112.4**	TCGA	0.2661	0.7087	0.5428	1.3056	0.1921	
									**h112.5**	TCCG	0.0144	1.0316	2.0147	0.5120	0.6088	
									**h112.6 (rare)**	****	0.0068	2.8715	3.2105	0.8944	0.3714	
									**hap.base112**	TGCG	0.6530	NA	NA	NA	NA	
**123**	p83884-rs701106	125265636	Intron 12		C/T	0.2597	1.2967	**0.0156**	**h123.1**	TCCT	0.0235	−1.7638	1.7393	−1.0141	0.3109	0.0468
**123**	p86245-rs188375019	125263275	Intron 12		C/T	0.0341	1.8399	0.1674	**h123.2**	TCCG	0.2351	1.8726	0.6006	3.1179	0.0019	
**123**	p86276-rs747155	125263244	Intron 12		C/T	0.1495	−0.2164	0.7433	**h123.3**	CCTG	0.1485	0.3912	0.6981	0.5604	0.5754	
**123**	p86316-rs701104	125263204	Intron 12		G/T	0.0487	−0.6627	0.5579	**h123.4**	CCCT	0.0238	1.6476	1.7546	0.9390	0.3480	
									**h123.5**	CTCG	0.0328	2.3144	1.3655	1.6949	0.0905	
									**h123.6 (rare)**	****	0.0024	1.2704	8.8153	0.1441	0.8855	
									**hap.base123**	CCCG	0.5340	NA	NA	NA	NA	

*ApoA-I* apolipoprotein A-I, *Coef* coefficient, *del/D* deletion, *HDL-C* high-density lipoprotein cholesterol, *MAF* minor allele frequency, *NA* not analyzed, *SE* standard error, *SNP* single nucleotide polymorphism, *UTR* untranslated region, *W* wild type allele for deletion on RefSeq

All alleles on the reverse strand. HDL-C and ApoA-I variables were in mg/dL and Box-Cox transformed

Results were adjusted for covariates: sex, age, waist, current smoking (yes/no), and minutes of daily walking or biking to work (jobmin) for HDL-C; sex and age for ApoA-I

SNP 1-SNP 4 for each window are shown as “SNP name-SNP ID/Chromosome 12 Position (for novel variants)”. All 10 novel variants identified in this study have been submitted to dbSNP database (batch ID: SCARB1_AB): http://www.ncbi.nlm.nih.gov/SNP/snp_viewTable.cgi?handle=KAMBOH.

Nominally significant *P*-values (*P* < 0.05) for SNPs with MAF ≥5 % in single-site analysis are shown in **bold**

Haplotype sequences corresponding to SNP 1-SNP 4 in the 5′ to 3′ direction, respectively

**Fig. 3:**
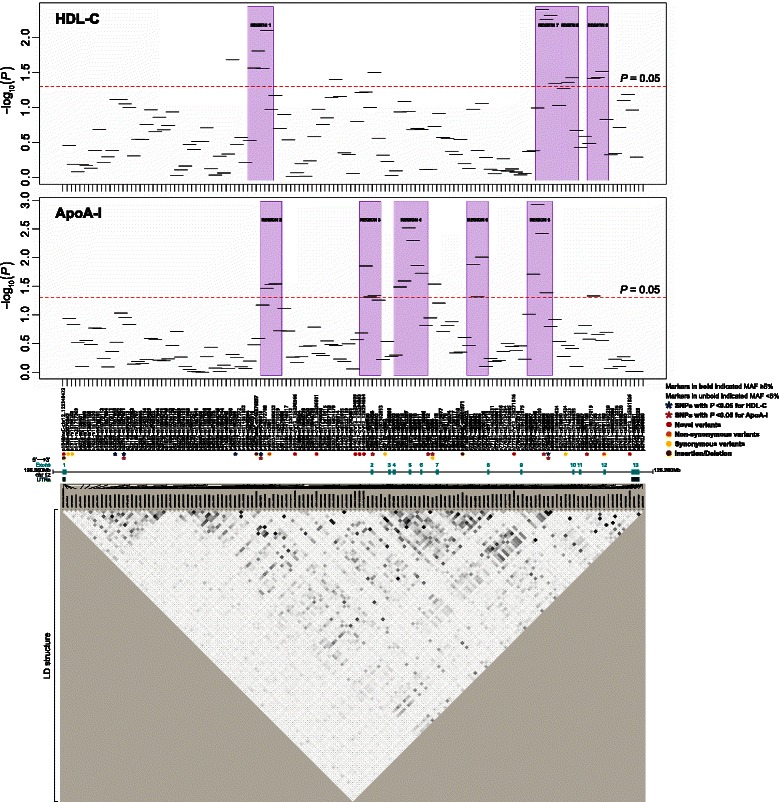
Haplotype association plots for HDL-C and ApoA-I. *Top*: The -log_10_ *P*-values are presented in the Y-axis. A total of 136 genotyped variants are shown in order on *SCARB1* gene (5′ → 3′; RefSeq: hg19, NM_005505) in the X-axis. *Middle*: gene structure of *SCARB1*. Marker names are shown as “SNP name-SNP ID/chromosome 12 position (for novel variants)”. *Bottom*: linkage disequilibrium (LD) plot of 136 variants. SNPs with MAF ≥5 % are shown in *bold*. SNP ID is based on dbSNP build 139. All 10 novel variants identified in this study have been submitted to dbSNP (batch ID: SCARB1_AB): http://www.ncbi.nlm.nih.gov/SNP/snp_viewTable.cgi?handle=KAMBOH. The dash line indicates the significance threshold (global *P* = 0.05). Significantly associated haplotype regions are highlighted. The degree of shades and values (*r*^2^ × 100) in each square of LD plot represent the pairwise correlations between 136 genotyped variants: black indicating *r*^2^ = 1, white indicating *r*^2^ = 0, and shade intensity indicating *r*^2^ between 0 and 1. ApoA-I, apolipoprotein A-I; HDL-C, high-density lipoprotein cholesterol; LD, linkage disequilibrium; MAF, minor allele frequency; SNP, single nucleotide polymorphism; UTR, untranslated region

Haplotype association results for all haplotype windows are shown in Additional file 16: Table S11, see haplotype association plots in Fig. 3

^a, c^RefSeq of *SCARB1*: hg19, NM_005505 (CHIP Bioinformatics)

^b^dbSNP build 139: GRCh37.p10

Overall, a total of 21 haplotype windows showed significant associations with ApoA-I, of which 10 contained seven variants associated with ApoA-I in single-site analysis. Haplotype window #110 spanning introns 10–11 showed the best association signal (global *P* = 0.0012) and contained the rs838896 variant with a nominal evidence of association with ApoA-I (*P* = 0.0278) in single-site analysis.

A total of 16 haplotype windows yielded significant associations with HDL-C, of which seven contained three HDL-C-associated variants detected in single-site analysis. The most significant association was found with window #111 (global *P* = 0.0040) spanning intron 11, which contained the rs838895 variant nominally associated with HDL-C (*P* = 0.0162) in single-site analysis.

We observed nine regions (5 regions for ApoA-I and 4 regions for HDL-C) harboring consecutive significant haplotype windows (global *P* < 0.05; ranging from 2 to 6 windows per region; Table 8 and Fig. 3). Seven of those regions contained at least one of the six variants that exhibited nominal associations (*P* < 0.05) with HDL-C and/or ApoA-I (rs4765615, rs7134858, rs838912, rs838896, rs838895, and rs701106) in single-site analysis.

**Table 8:** Significantly associated haplotype regions (global *P* < 0.05) with HDL-C and ApoA-I

Region #	Trait	Consecutive Significantly Associated Haplotype Windows (global *P* < 0.05)							
		Haplotype Windows #	Chr12 Position^a^		The Composited Variants in the Region, 5′ to 3′ Direction		Most Relevant Haplotype		
			(Location)						
			Start (5′)	End (3′)	SNP Name^b^-SNP ID^c^/Chr12 Position^a^	Major/Minor Alleles	Haplotype #	Sequence	β (Min-Max)
**1**	HDL-C	44	125300551	125299542	p48969-rs2343394	C/T	h44.3	CCWGCGG	0.4910–1.0491
		45	(intron 2)	(exon 3)	p49537-rs7305310	C/T	h45.2		
		46			p49570delC-rs145376237	W/D	h46.2		
		47			**p49690-rs4765615**	G/A	hap.base47		
					p49759-rs146272788	C/T	hap.base44	CCWACGG	−0.4701
					p49978-rs5891 (p.Val135Ile)	G/A	hap.base45		
					p50024-rs368880622	G/T	hap.base46		
							h47.1		
**2**	ApoA-I	47	125299830	125299369	**p49690-rs4765615**	G/A	h47.1	ACGGTT	(−0.8907)–3.3792
		48	(intron 2)	(intron 3)	p49759-rs146272788	C/T	h48.1		
		49			p49978-rs5891 (p.Val135Ile)	G/A	h49.1		
					p50024-rs368880622	G/T			
					p50118-rs58710319	C/T			
					p50151-rs2278986	T/C			
**3**	ApoA-I	70	125294893	125292516	p54627-chr12_125294893	G/C	h70.2	GCGTAG	2.0304–2.1103
		71	(intron 5)	(intron 6)	p54856-chr12_125294664^d^	C/T	h71.3		
		72			p55923-rs838900	G/A	h72.3		
					**p55963-rs7134858**	C/T			
					p56845-rs838902	A/G			
					p57004-rs187562853	G/A			
**4**	ApoA-I	78	125291928	125286037	p57592-rs838903	G/A	h78.5	GTTTCGCTG	4.7307–5.5715
		79	(intron 7)	(intron 7)	p58514-rs838905	T/C	h79.6		
		80			p58664-rs865716	A/T	h80.6		
		81			p60255-rs3782287	C/T	hap.base81		
		82			p61872-rs838909	C/T	hap.base82		
		83			p62140-rs838910	G/T	hap.base83		
					p62409-rs838911	C/T	h78.2	GTACCTCTG	0.6384–3.8641
					p62615-rs7138386	T/C	h79.2		
					**p63483-rs838912**	G/A	hap.base80		
							h81.3		
							h82.2		
							h83.4		
**5**	ApoA-I	95	125277653	125272763	p71867-rs7954022	C/T	h95.5	CGTTCT	4.2363-4.7525
		96	(intron 9)	(intron 9)	p72197-rs838861	A/G	h96.5		
		97			p72777-rs838862	C/T	h97.4		
					p75766-rs838866	T/C			
					p75778-rs7301120	C/T			
					p76757-rs9919713	A/T			
6*	ApoA-I	109	125271118	125269475	p78402-rs838898	G/A	h109.6	GCCTGCA	(−3.3720)─(−1.8104)
		**110**	(intron 10)	(intron 11)	p78430-rs838897	C/G	h110.6		
		111			p78747-rs2293440	T/C	h111.2		
		112			p78791-rs75289200	T/C	h112.3		
					**p79721-rs838896**	G/C			
					**p79828-rs838895**	C/G			
					p80045-rs838893	G/A			
7*	HDL-C	**111**	125270773	125267501	p78747-rs2293440	T/C	h111.4	TTGGAGC	0.3755–2.3949
		112	(intron 11)	(intron 11)	p78791-rs75289200	T/C	h112.2		
		113			p79721-rs838896	G/C	h113.1		
		114			**p79828-rs838895**	C/G	h114.1		
					p80045-rs838893	G/A			
					p81863-rs185445624	G/A			
					p82019-rs838890	C/T			
**8**	HDL-C	117	125267501	125267086	p82019-rs838890	C/T	h117.2	TCGGC	(−1.0134)–(−0.9657)
		118	(intron 11)	(intron 12)	p82264-rs141545424 (p.Gly501Gly)^d^	C/A	h118.2		
					p82340-rs77483223	G/A			
					p82369-rs75446635	G/A			
					p82434-rs838889	T/C			
**9**	HDL-C	123	125265636	125262553	***p83884-rs701106***	C/T	h123.4	CCCTGA	(−1.180)–(−0.2329)
		124	(intron 12)	(exon 13-3′ UTR)	p86245-rs188375019	C/T	h124.2		
		125			p86276-rs747155	C/T	h125.2		
					p86316-rs701104	G/T			
					p86481-rs701103 (p.Gly499Arg, isoform 2)	G/A			
					p86967-rs187492239	A/G			

*ApoA-I* apolipoprotein A-I, *del/D* deletion, *HDL-C* high-density lipoprotein cholesterol, *SNP* single nucleotide polymorphism, *UTR* untranslated region, *W* wild type allele for deletion on the RefSeq

All alleles on the reverse strand. HDL-C and ApoA-I variables were in mg/dL and Box-Cox transformed

Results were adjusted for covariates: sex, age, waist, current smoking (yes/no), and minutes of daily walking or biking to work (jobmin) for HDL-C; sex and age for ApoA-I

All nine haplotype regions are shown in Fig. 3

Detailed single-site associations are shown in Additional file 14: Table S9 and Additional file 15: Table S10

Detailed haplotype associations are shown in Table 7 and Additional file 16: Table S11

Regions with asterisk (*) indicate regions that included the haplotype window exhibiting the most significant association signal (the smallest global *P*) for the associated trait

For each region, the most significant associated haplotype window is shown in **bold**

SNPs with significant evidence of association with the same trait in both single-site and haplotype analyses (single-site *P* < 0.05 and global *P* < 0.05) are shown in **bold**

SNPs with significant evidence of association with different trait in single-site and haplotype analyses (single-site *P* < 0.05 and global *P* < 0.05) are shown in *italic* **bold**

^a, b^RefSeq of *SCARB1*: hg19, NM_005505 (CHIP Bioinformatics)

^c^dbSNP build 139: GRCh37.p10

^d^Rare variants of interest with potential effects on lipid traits; see details in Table 6

### Functional evaluation of identified variants

In order to examine the possible regulatory function of all 153 *SCARB1* variants (83 variants identified by our sequencing, 68 common HapMap tagSNPs [excluding rs4765180 due to genotyping failure; see Additional file 7: Table S5], and two relevant variants from the literature), we used the RegulomeDB database (version 1.0, Stanford University, http://www.regulomedb.org/) [48]. Although most of 153 variants (*n* = 132) revealed scores ranging from 1 to 6, only 11 were supported by strong evidence for regulatory function (scores of 1f -2b): one promoter, one 5′ UTR, two coding (rs2070242 [p.Ser4Ser] and rs10396208 [p.Cys21Cys]), five intronic, one 3′ UTR, and one 3′ flanking variants. Summary and detailed regulatory functions are provided in Additional file 17: Table S12 and Additional file 18: Table S13.

Of 10 variants associated with HDL-C and/or ApoA-I, only one ApoA-I associated variant (rs5888 [p.Ala350Ala] in exon 8) showed suggestive evidence of regulatory function with a score of 3a (Table 4).

Of 10 novel variants, one insertion variant (p1048insC/chr12:125348472) located in 5′ UTR-exon 1 had a strong potential for regulatory function with a score of 2a (Additional file 4: Table S4).

### Comparison of *SCARB1* single-site and haplotype association analysis results between African Blacks (this study) and US Non-Hispanic Whites (previous study [49])

**Fig. 4:**
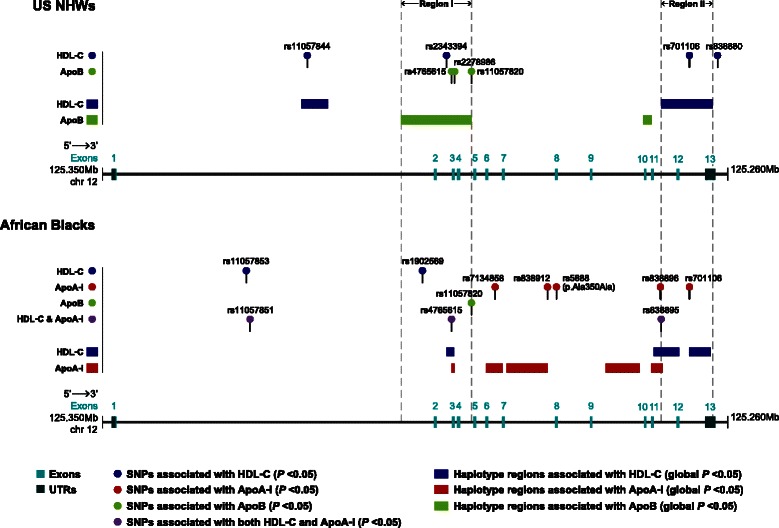
Lipid-associated *SCARB1* common variants and haplotype regions identified in US Non-Hispanic Whites (previous study; Ref [49]) and African Blacks (this study). Lipid-associated variants with MAF ≥5 % with *P*-values <0.05 and haplotype regions with global *P*-values < 0.05 that were previously identified in US Non-Hispanic Whites (US NHWs; *n* = 623) are shown in *top panel* and those identified in African Blacks (*n* = 788) are shown in *bottom panel* (see details in Table 9 and Table 10). *SCARB1* variants and haplotype regions are shown on *SCARB1* gene (5′ → 3′; RefSeq: hg19, NM_005505). All SNP IDs are based on dbSNP build 139. Regions I and II that are defined based on consecutive haplotype windows with evidence of lipid-association in US NHWs (global *P* < 0.05; see details in Ref [49]) also show some significant associations in African Blacks (global *P* < 0.05; see details in Table 7 and Table 8). ApoA-I, apolipoprotein A-I; ApoB, apolipoprotein B; HDL-C, high-density lipoprotein cholesterol; MAF, minor allele frequency; NHW, Non-Hispanic White; SNP, single nucleotide polymorphism; UTR, untranslated region

We compared *SCARB1* single-site and haplotype association results in African Blacks reported in this study to those in US Non-Hispanic Whites (NHWs) reported in our previously published study [49]. In the sequencing stage, the number of variants identified in African Blacks (*n* = 83) was greater than that in US NHWs (*n* = 44). Notably, most (~90 %) of the 22 sequence variants that were shared between the two populations differed in minor alleles and/or MAFs. Although our major findings included the associations with HDL-C and ApoA-I in African Blacks, we also sought to replicate four associations observed with ApoB levels in US NHWs [49] (Table 9); the association between rs11057820 and ApoB (*P* < 0.05) that we previously reported in US NHWs [49] was also observed in African Blacks (US NHWs [G allele]: β = 0.8700, *P* = 0.0436; African Blacks [A allele]: β = 1.8661, *P* = 0.0292). In addition, we observed two variants (rs4765615 and rs701106) exhibiting nominal associations (*P* < 0.05) in both populations, albeit with different lipid traits (US NHWs| rs4765615 [G allele]: β = 1.2493, *P* = 0.0059 for ApoB; rs701106 [T allele]: β = 0.0394, *P* = 0.0066 for HDL-C; African Blacks| rs4765615 [A allele]: β = −0.4646, *P* = 0.013 for HDL-C and β = −0.9139, *P* = 0.048 for ApoA-I; rs701106 [T allele]: β = 1.2967, *P* = 0.0156 for ApoA-I). Moreover, we noticed that two regions associated with HDL-C or ApoA-I (global *P* < 0.05; Table 10) in African Blacks spanning intron 2 and intron 3 overlapped with the ApoB-associated region (Region I in Fig. 4) previously reported in US NHWs [49]. Three haplotype regions associated with HDL-C (global *P* < 0.05) spanning intron 11 and exon 13-3′ UTR in African Blacks also overlapped with a large HDL-C-associated region (Region II in Fig. 4) previously reported in US NHWs [49].

**Table 9:** Results for 7 *SCARB1* lipid-associated variants in US Non-Hispanic Whites (previous study^a^) and in African Blacks (this study)

SNP Name^b^	SNP ID^c^	Chr12 Position^d^	Location	RegDB Score^e^	Alleles	US Non-Hispanic Whites^a^ (*n* = 623)			African Blacks (*n* = 788)			
						MA, MAF	β	*P*	MA, MAF	β	*P*	Other Assoc Trait(s)^f^
							(SE)			(SE)		
HDL-C												
p28957	rs11057844	125320563	Intron 1	5	G/A	A, 0.1839	−0.0395	**0.0035**	A, 0.2362	0.3671	0.1075	
							(0.0135)			(0.2278)		
p83884	rs701106	125265636	Intron 12	5	C/T	T, 0.1527	0.0394	**0.0066**	T, 0.2597	0.2471	0.2601	ApoA-I
							(0.0144)			(0.2192)		
p87927	rs838880	125261593	3′ flanking	5	G/A	G, 0.3237	0.0257	**0.0250**	A, 0.2414	0.0198	0.9314	
							(0.0114)			(0.2302)		
ApoB												
p48969	rs2343394	125300551	Intron 2	5	C/T	T, 0.2850	1.2544	**0.0082**	T, 0.1898	0.0383	0.9544	
							(0.4721)			(0.6696)		
p49690	rs4765615	125299830	Intron 2	5	G/A	G, 0.4497	1.2493	**0.0059**	A, 0.4426	0.7771	0.1338	HDL-C, ApoA-I
							(0.4518)			(0.5178)		
p50151	rs2278986	125299369	Intron 3	5	T/C	C, 0.2890	1.1926	**0.0122**	C, 0.1933	0.1308	0.8434	
							(0.4735)			(0.6619)		
p52556	rs11057820	125296964	Intron 4	5	G/A	G, 0.4871	0.8700	**0.0436**	A, 0.1000	1.8661	**0.0292**	
							(0.4300)			(0.8542)		

*ApoB* apolipoprotein B, *HDL-C* high-density lipoprotein cholesterol, *MA* minor allele, *MAF* minor allele frequency, *RegDB* RegulomeDB, *SE* standard error, *SNP* single nucleotide polymorphism

All alleles on the reverse strand

HDL-C and ApoB values for US Non-Hispanic Whites were in mg/dL, Box-Cox transformed, and adjusted for covariates: sex, age, body mass index, and smoking (past/current/never) for HDL-C; age and smoking for ApoB

HDL-C and ApoB values for African Blacks were in mg/dL, Box-Cox transformed, and adjusted for covariates: sex, age, waist, current smoking (yes/no), and daily walking or biking to work (jobmin) for HDL-C; body mass index and staff status for ApoB

Nominally significant *P*-values (*P* < 0.05) are shown in **bold**

^a^Data from Niemsiri V, et al. *Circ Cardiovasc Genet* 2014, **7**(6):838–847 (Ref [49])

^b, d^RefSeq of *SCARB1*: hg19, NM_005505 (CHIP Bioinformatics)

^c^dbSNP version 139: GRCh37.p10

^e^The RegulomeDB (version 1.0) scoring scheme is described at the footnote of Additional file 17: Table S12 or at http://regulome.stanford.edu/help

^f^Evidence is based on SNPs with MAF ≥5 % exhibiting nominally significant association with either HDL-C or ApoA-I (*P* < 0.05; Additional file 14: Table S9 and Additional file 15: Table S10) in single-site association results in the current study

**Table 10:** Significant lipid-associated regions (global *P* < 0.05) that were observed in US Non-Hispanic Whites (previous study^a^) and African Blacks (this study)

Region #	Consecutive Haplotype Windows in 623 US Non-Hispanic Whites^a^						Consecutive Haplotype Windows in 788 African Blacks					
	Trait	Chr12 Position^b^ (Location)		Length (bp)	The Composited Variants, 5′ to 3′ Direction		Trait	Chr12 Position^b^ (Location)		Length (bp)	The Composited Variants, 5′ to 3′ Direction	
		Start (5′)	End (3′)		SNP Name^c-^SNP ID^d^	Major/Minor Alleles		Start (5′)	End (3′)		SNP Name^c^-SNP ID^d^	Major/Minor Alleles
**I**	**ApoB**	125300551	125299369	1183	p48969-rs2343394	C/T	**HDL-C**	125300551	125299496	1056	p48969-rs2343394	C/T
		(intron 2)	(intron 3)		p49518-rs144194221	G/A		(intron 2)	(intron 3)		p49537-rs7305310	C/T
					**p49690-rs4765615**	A/G					p49570delC-rs145376237	W/D
					p49978-rs5891	G/A					**p49690-rs4765615**	G/A
					(p.Val135Ile)							
					p50151-rs2278986	T/C					p49759-rs146272788	C/T
											p49978-rs5891	G/A
											(p.Val135Ile)	
											p50024-rs368880622	G/T
							**ApoA-I**	125299830	125299369	462	**p49690-rs4765615**	G/A
								(intron 2)	(intron 3)		p49759-rs146272788	C/T
											p49978-rs5891	G/A
											(p.Val135Ile)	
											p50024-rs368880622	G/T
											p50118-rs58710319	C/T
											p50151-rs2278986	T/C
**II**	**HDL-C**	125269692	125262516	7177	p79828-rs838895	C/G	**HDL-C**	125269692	125267501	2192	**p79828-rs838895**	C/G
		(intron 11)	(exon 13- 3′ UTR)		p80045-rs838893	G/A		(intron 11)	(intron 11)		p80045-rs838893	G/A
					p83088-rs797729	A/G					p81863-rs185445624	G/A
					**p83884-rs701106**	C/T					p82019-rs838890	C/T
					p86436-rs10396214	C/T	**HDL-C**	125267501	125267086	416	p82019-rs838890	C/T
					(p.Arg484Trp, isoform 2)							
					p87004-rs184715678	C/A		(intron 11)	(intron 12)		p82264-rs141545424	C/A
											(p.Gly501Gly)	
											p82340-rs77483223	G/A
											p82369-rs75446635	G/A
											p82434-rs838889	T/C
							**HDL-C**	125265636	125262553	3084	***p83884-rs701106***	C/T
								(intron 12)	(exon 13- 3′ UTR)		p86245-rs188375019	C/T
											p86276-rs747155	C/T
											p86316-rs701104	G/T
											p86481-rs701103	G/A
											(p.Gly499Arg, isoform 2)	
											p86967-rs187492239	A/G

*ApoA-I* apolipoprotein A-I, *ApoB* apolipoprotein B, *del/D* deletion, *HDL-C* high-density lipoprotein cholesterol, *SNP* single nucleotide polymorphism, *UTR* untranslated region, *W* wild type allele for deletion on RefSeq

All alleles on the reverse strand

Results for a US Non-Hispanic White sample were Box-Cox transformed, and adjusted for covariates: sex, age, body mass index, and smoking (past/current/never) for HDL-C; age and smoking for ApoB

Results for an African Black sample were Box-Cox transformed, and adjusted for covariates: sex, age, waist, current smoking (yes/no), and minutes of walking or biking to work each day (jobmin) for HDL-C; sex and age for ApoA-I

Location of each region on *SCARB1* gene is shown in Fig. 4

SNPs with significant evidence with the same trait in both single-site and haplotype associations (single-site *P* and global *P* < 0.05) observed in each population are shown in **bold**

SNPs with significant evidence with the different trait in single-site and haplotype associations (single-site *P* and global *P* < 0.05) in each population are shown in ***italic bold***

^a^Data from Niemsiri V, et al. *Circ Cardiovasc Genet* 2014, **7**(6):838–847 (Ref [49])

^b, c^RefSeq of *SCARB1*: hg19, NM_005505 (CHIP Bioinformatics)

^d^dbSNP version 139: GRCh37.p10

## Discussion

Our sequencing identified 83 variants, of which 78 were selected for follow-up genotyping in the total sample of 788 African Blacks. Additional 69 tagSNPs from the HapMap-YRI data along with two previously reported lipid-associated *SCARB1* variants were also genotyped in the total sample. Of 149 genotyped *SCARB1* variants, 137 that passed QC were examined for association with major lipid traits (Table 2). The initial gene-based analyses revealed a nominal association with HDL-C (*P* = 0.0421) as well as a trend for association with ApoA-I (*P* = 0.1016; Table 3). Consistent with the gene-based results, single-site association analyses also revealed 10 common variants nominally associated (*P* < 0.05) with HDL-C (*n* = 5) and/or ApoA-I (*n* = 8; Table 4 and Fig. 2). The best association signal was between rs11057851 in intron 1 and HDL-C (*P* = 0.0043, FDR = 0.1465) followed by two associations with ApoA-I including rs7134858 in intron 6 (*P* = 0.0052, FDR = 0.2918) and rs5888 (p.Ala350Ala) in exon 8 (*P* = 0.0080, FDR = 0.2918). Moreover, three variants (rs11057851, rs4765615, and rs838895) exhibited evidence of associations (*P* < 0.05) with both HDL-C and ApoA-I. These findings are supported by the fact that *SCARB1* appears to influence ApoA-I in addition to HDL-C [15, 17]. In our data, there was a moderate correlation between ApoA-I and HDL-C levels (*r*^2^ = 0.61).

Except for previously reported association of rs5888 (p.Ala350Ala) with lipid traits (HDL-C or LDL-C) in non-African populations [30–34, 36, 37, 39], the remaining nine associations observed in this study with the lipid traits (HDL-C and/or ApoA-I levels) in general population are novel and await replication in independent African or African-derived populations. Two of these nine SNPs have previously been shown to have differential effects on cholesterol levels in response to statin (rs4765615) [50] or on HDL-C/TG levels in response to estradiol in post-menopausal women (rs838895) [51]. Another variant (rs838896) was found to be associated with decreased *SCARB1* expression in liver [51]. Although the latter SNP was not associated with a low RegulomeDB score (<3), we cannot rule out the possibility that it might be affecting the *SCARB1* expression in a tissue-specific manner.

The haplotype analysis revealed evidence of significant association (global *P* < 0.05) of 32 haplotype windows with HDL-C (*n* = 16) and/or ApoA-I (*n* = 21; Table 7) and nine regions harboring consecutive overlapping haplotype windows significantly associated with either HDL-C (4 regions) or ApoA-I (5 regions; Table 8 and Fig. 3). In addition, six variants with nominal association (*P* < 0.05) in single-site analysis were contained in seven of these nine significantly associated regions, indicating the presence of functional variants in these regions. Our findings demonstrate that haplotype analysis may provide more information than single-site analysis.

Our comparison of the single-site and haplotype association results between in African Blacks (this study) and US NHWs (previous study [49]) has revealed three variants (rs11057820, rs4765615, and rs701106; Table 9) and two regions (Regions I and II; Table 10 and Fig. 4) showing evidence of lipid-associations in both ethnic groups. However, there were differences in associated traits, and/or associated alleles or their directional effects between the two ethnic groups, which reflects the genetic heterogeneity of complex phenotypes like lipid traits among diverse populations. This phenomenon can be explained by different ancestry backgrounds associated with differences in LD structure and genetic architecture, as well as by differences in SNP-SNP, gene-gene, and gene-environment interactions. Nonetheless, the lipid associations observed across different ethnic populations provide convincing evidence that causal/functional variants are present in *SCARB1* gene that deserves comprehensive sequencing and functional studies in order to confirm and further characterize the effects of its variants on lipid metabolism.

Rare variant analysis showed significant evidence of association between a group of 23 rare variants (MAF ≤1 %) and HDL-C (*P* = 0.0478; Table 5). Single-site analysis of these rare variants revealed six (including three novel ones) with effects on HDL-C, of which three also had effects on ApoA-I (Table 6). In addition, four of these six rare variants appeared to be carried by individuals with extreme HDL-C and/or ApoA-I levels (above or under the 3rd percentile). This HDL-C-associated rare variant group also included a novel variant (p70201/chr12:125279319) that was observed in one individual with an unusually high plasma HDL-C level (above the mean + 3.5 SD). Our findings suggest that these rare variants might have functional relevance, thus screening of additional large African samples for these rare variants may help to establish their role in HDL-C and ApoA-I metabolism.

To date, there has been limited information concerning possible functional effects of lipid-associated *SCARB1* variants, particularly for those located in non-coding regions. In fact, most of common and rare HDL-C/ApoA-I-associated variants observed in the current study are non-coding and do not show strong evidence of regulatory function based on RegulomeDB database. Nonetheless, three of these HDL-C/ApoA-I-associated *SCARB1* variants (rs5888 [p.Ala350Ala], rs838885, and rs838886) have been previously demonstrated to influence the *SCARB1* expression [51–53]. Therefore, additional functional studies are needed and may help to determine the functional nature of the *SCARB1*-associated variants and those in LD with them.

Our study has revealed a number of novel findings, although we also acknowledge some limitations. *SCARB1* is a large gene and we sequenced only its coding regions and exon-intron junctions and also our sequencing sample size was small. Thus, we may have missed some functional LoF/rare variants due to small sample size and those located in uncovered intronic regions. Moreover, consistent with generally small effect sizes of lipid-associated variants reported in the literature, most of our single-site associations reached nominal significance (*P* < 0.05) but did not survive multiple testing corrections. Only the top variant (rs11057851) associated with HDL-C yielded an FDR cut-off of <0.20 (FDR = 0.1465; Table 4). Therefore, future larger studies in independent African or African-derived populations are necessary to validate all nominal associations observed in this study.

## Conclusions

In conclusion, we report the first comprehensive association study of *SCARB1* variants with lipid traits in a native African population, which revealed a number of novel associations in single-site and haplotype analyses. In addition, resequencing allowed us to identify 10 novel rare variants, of which four were in the group of 23 rare variants that has showed association with HDL-C levels. The *SCARB1* associated common and rare variants observed in our study explained ~11.09 % of the variation in HDL-C levels and ~8.63 % of the variation in ApoA-I levels. Our findings indicate the genetic contribution of *SCARB1*, both common and LoF/rare variants, to inter-individual lipid variation in the general African Black population, which warrants further follow-up in independent studies. Insights into the HDL-C and related lipid traits may also lead to new potential targets for CHD treatment.

## Additional files

Additional file 1: Table S1. Characteristics and lipid profile of the entire sample of 788 African Blacks stratified by sex. (PDF 63 kb)
Additional file 2: Table S2. Primer sequences for 14 polymerase chain reaction (PCR) fragments and the sizes of 13 *SCARB1* exons. (PDF 96 kb)
Additional file 3: Table S3. Characteristics of 83 *SCARB1* sequence variants identified in 95 African Blacks with extreme HDL-C levels. (PDF 146 kb)
Additional file 4: Table S4. Characteristics of 10 *SCARB1* novel variants identified by sequencing. (PDF 81 kb)
Additional file 5: Figure S1. Linkage disequilibrium (LD) plot of 83 *SCARB1* sequence variants. Of 83 sequence variants (see the list in Additional file 3: Table S3), 78 were selected for genotyping. An enlarged view of the part of LD plot (**A**) shows the pairwise correlations (*r*^2^) between four variants including the two variants (shown in **bold**) in the same bin in our data, of which one selected for genotyping. This bin was not identified by Tagger analysis of common *SCARB1* variants in the HapMap-YRI data (see Additional file 7: Table S5 and Additional file 8: Figure S3). The degree of shades and values (*r*^2^ × 100) in each square of LD plot represent the pairwise correlations: black indicating *r*^2^ = 1, white indicating *r*^2^ = 0, and shade intensity indicating *r*^2^ between 0 and 1. LD, linkage disequilibrium; MAF, minor allele frequency; YRI, Yoruba people of Ibadan from Nigeria. (PDF 920 kb)
Additional file 6: Figure S2. Linkage disequilibrium (LD) plot of 32 *SCARB1* common sequence variants. Enlarged view of the parts of the LD plot (**A**, **B**, and **C**) show three LD bins (identified by Tagger analysis of variants with minor allele frequency (MAF) ≥5 % using an *r*^*2*^ cutoff of 0.90) containing more than one variant (*r*^*2*^ ranging between 0.95 and 1.0). The degree of shades and values (*r*^2^ × 100) in each square of LD plot represent the pairwise correlations: black indicating *r*^2^ = 1, white indicating *r*^2^ = 0, and shade intensity indicating *r*^2^ between 0 and 1. LD, linkage disequilibrium; MAF, minor allele frequency. (PDF 370 kb)
Additional file 7: Table S5. List of 77 *SCARB1* HapMap-YRI tagSNPs. (PDF 110 kb)
Additional file 8: Figure S3. Linkage disequilibrium (LD) plot of 108 *SCARB1* common HapMap-YRI tagSNPs. The list of 77 common HapMap-YRI tagSNPs identified by Tagger analysis of variants with minor allele frequency ≥5 % using an *r*^2^ cutoff of 0.80 is shown in Additional file 7: Table S5. The degree of shades and values (*r*^2^ × 100) in each square of LD plot represent the pairwise correlations: black indicating *r*^2^ = 1, white indicating *r*^2^ = 0, and shade intensity indicating *r*^2^ between 0 and 1.LD, linkage disequilibrium; SNP, single nucleotide polymorphism; YRI, Yoruba people of Ibadan from Nigeria. (TIFF 2642 kb)
Additional file 9: Table S6. Characteristics of 138 *SCARB1* variants genotyped in the entire sample of 788 African Blacks. (PDF 212 kb)
Additional file 10: Table S7. List of 87 *SCARB1* genotyped common tagSNPs. (PDF 108 kb)
Additional file 11: Figure S4. Linkage disequilibrium (LD) plot of 137 *SCARB1* genotyped variants. The list of 87 genotyped common tagSNPs identified by Tagger analysis for variants with minor allele frequency ≥5 % using an *r*^2^ cutoff of 0.90 is shown in Additional file 10: Table S7. The degree of shades and values (*r*^2^ × 100) in each square of LD plot represent the pairwise correlations: black indicating *r*^2^ = 1, white indicating *r*^2^ = 0, and shade intensity indicating *r*^2^ between 0 and 1.LD, linkage disequilibrium; SNP, single nucleotide polymorphism. (TIFF 3095 kb)
Additional file 12: Figure S5. Location and minor allele frequency (MAF) distributions of 137 *SCARB1* genotyped variants. Details for each variant are shown in Additional file 9: Table S6. MAF, minor allele frequency; UTR, untranslated region. (TIFF 324 kb)
Additional file 13: Table S8. Covariates used for the statistical analysis of lipid variables. (PDF 78 kb)
Additional file 14: Table S9. Single-site association results for 136 *SCARB1* genotyped variants with HDL-C. (PDF 689 kb)
Additional file 15: Table S10. Single-site association results for 136 *SCARB1* genotyped variants with ApoA-I. (PDF 686 kb)
Additional file 16: Table S11. Haplotype association results for 136 *SCARB1* genotyped variants for HDL-C and ApoA-I. (PDF 257 kb)
Additional file 17: Table S12. Summary of RegulomeDB scores of 153 *SCARB1* variants. (PDF 95 kb)
Additional file 18: Table S13. RegulomeDB scores and functional assignments of 153 *SCARB1* variants. (PDF 149 kb)

## Abbreviations

ApoA-I: Apolipoprotein A-I
ApoB: Apolipoprotein B
BP: Base pair
CE: Cholesteryl esters
CHD: Coronary heart disease
FDR: False discovery rate
GWAS: Genome-wide association studies
HDL-C: High-density lipoprotein cholesterol
HWE: Hardy-Weinberg equilibrium
indel: Insertion and deletion variation
KB: Kilobase pair
LD: Linkage disequilibrium
LDL-C: Low-density lipoprotein cholesterol
LoF: Low-frequency
MAF: Minor allele frequency
NHW: Non-Hispanic White
PCR: Polymerase chain reaction
QC: Quality controls
RCT: Reverse cholesterol transport
SCARB1: Scavenger receptor class B member 1
SD: Standard deviation
SKAT-O: An optimal sequence kernel association test
SNP: Single nucleotide polymorphism
SNV: Singlenucleotide variation
TG: Triglycerides
UTR: Untranslated region
VEGAS: Versatile gene-based association study
VLDL-C: Very low-density lipoprotein cholesterol
YRI: Yoruba people of Ibadan from Nigeria
