# Supplementary material for: Genetic contribution of SCARB1 variants to lipid traits in African Blacks: a candidate gene association study
Source: BMC Med Genet. 2015 Nov 12;16:106. doi: 10.1186/s12881-015-0250-6 (PMC4643515; doi:10.1186/s12881-015-0250-6)
Supplement: Additional file 1: Table S1. — Characteristics and lipid profile of the entire sample of 788 African Blacks stratified by sex. (PDF 63 kb) [file 12881_2015_250_MOESM1_ESM.pdf]

Table S1. Characteristics and lipid profile of the entire sample of 788 African Blacks stratified by sex.

Variables	Males[a]	Females[a]	The Entire Sample[a]
**N**	495	293	788
**Age, years**	42.35 ± 8.62	38.60 ± 7.43	40.95 ± 8.39
**BMI, kg/m^2**	22.03 ± 3.31	24.31 ± 4.71	22.87 ± 4.04
**Total Cholesterol, mg/dL**	166.65 ± 37.37	181.02 ± 38.69	172.01 ± 38.47
**LDL-Cholesterol, mg/dL**	104.52 ± 33.71	117.20 ± 34.14	109.25 ± 34.40
**HDL-Cholesterol, mg/dL**	46.03 ± 12.61	51.00 ± 12.71	47.88 ± 12.87
**Triglycerides, mg/dL**	78.72 ± 42.13	63.27 ± 31.88	72.96 ± 39.32
**Apolipoprotein A-I, mg/dL**	136.13 ± 29.37	138.54 ± 26.85	137.03 ± 28.46
**Apolipoprotein B, mg/dL**	65.35 ± 21.71	69.69 ± 22.74	66.98 ± 22.19

BMI, body mass index; HDL-Cholesterol, high-density lipoprotein cholesterol; LDL-Cholesterol, low-density lipoprotein cholesterol.

Values are presented as unadjusted means ± standard deviation (SD) unless otherwise mentioned.

[a] All data were unadjusted and included individuals with missing values or outliers (values beyond mean ± 3.5 SD).
