# Supplementary material for: Genetic contribution of SCARB1 variants to lipid traits in African Blacks: a candidate gene association study
Source: BMC Med Genet. 2015 Nov 12;16:106. doi: 10.1186/s12881-015-0250-6 (PMC4643515; doi:10.1186/s12881-015-0250-6)
Supplement: Additional file 2: Table S2. — Primer sequences for 14 polymerase chain reaction (PCR) fragments and the sizes of 13 SCARB1 exons. (PDF 96 kb) [file 12881_2015_250_MOESM2_ESM.pdf]

Table S2. Primer sequences for 14 polymerase chain reaction (PCR) fragments, and the sizes of 13 *SCARB1* exons.

Fragment	Forward Primer Sequence	Reverse Primer Sequence	Size of PCR Fragment (bp)	Size of Exon (bp)
**Exon 1**	5′-CCCATAGACGTTTTGGCTCA-3′	5′-AGCAACCTGTCCACACTCCT-3′	896	379
**Exon 2**	5′-TCCCTGCTGTGTTTTCTCTG-3′	5′-CTGGTGATTCGCACCTGTAA-3′	844	158
**Exon 3**	5′-TGGCTTGGAGAGATGAGAGA-3′	5′-TGGGAAACTCAGAACCCACT-3′	752	142
**Exon 4**	5′-CTGTCTTGTTGAGGGCTGAG-3′	5′-CAGGTTGCCAAGAGTCAAGA-3′	848	204
**Exon 5**	5′-CTCCTAGAAAGCTCCCAAGC-3′	5′-TTCACCTCCTGTGTTCAAGC-3′	791	96
**Exon 6**	5′-CTCACCTGCTCACCACACTT-3′	5′-TGATGTCTCAGCACCCAGAT-3′	770	116
**Exon 7**	5′-ACAGGTGTGAGCCATAATGC-3′	5′-GGTATCCAGGAGAGCACGAT-3′	989	167
**Exon 8**	5′-AGGGACACACTCCTGTGGAT-3′	5′-ACAGAACTTCACACGGGACA-3′	1042	119
**Exon 9**	5′-TCAGGAGAGGAGATCCCAGT-3′	5′-CGTGTAGGAAACAGCTTGGA-3′	906	74
**Exon 10**	5′-TGAGGGTAAGAAATGGCAGA-3′	5′-ACAAGCTAGGCCAGAAGGAA-3′	862	52
**Exon 11**	5′-TCCTTCTGGCCTAGCTTGTT-3′	5′-GGTGCTGACTTGATGAATGG-3′	990	147
**Exon 12**	5′-ACATGCGGGTAAACTCAACA-3′	5′-AGCCTTGCTCCTGTCTTTCT-3′	1033	129
**Exon 13 (1)**	5′-GCTCTGCCCCTCACTGTATT-3′	5′-TGTCAGTTTAGGCTGGAGGA-3′	978	
**Exon 13 (2)**	5′-ATGGAGTGAGCACAAGATGC-3′	5′-TAAGGGGAAAAGGGCTAACA-3′	1038	959

RefSeq of *SCARB1*: hg19, NM_005505 (CHIP Bioinformatics, University of Florida, http://snpper.chip.org/).
