# Supplementary material for: Genetic contribution of SCARB1 variants to lipid traits in African Blacks: a candidate gene association study
Source: BMC Med Genet. 2015 Nov 12;16:106. doi: 10.1186/s12881-015-0250-6 (PMC4643515; doi:10.1186/s12881-015-0250-6)
Supplement: Additional file 3: Table S3. — Characteristics of 83 SCARB1 sequence variants identified in 95 African Blacks with extreme HDL-C levels. (PDF 146 kb) [file 12881_2015_250_MOESM3_ESM.pdf]

Table S3. Characteristics of 83 *SCARB1* sequence variants identified in 95 African Blacks with extreme HDL-C levels.

									SEQ MAF					
**SNP Name[a]**	**SNP ID[b]**	**Chr12 Position[c]**	**Location**	**Amino Acid Change**	**Indels**	**Major/ Minor Alleles**	**HWE *P***	**%Call Rate**	**Total 95 Subjects**	**High HDL-C Group (48 subjects)**	**Low HDL-C Group (47 subjects)**	**Selection for GT (n = 78)**	**Success in GT (n = 68)**	**Note**
p972	rs181338950	125348548	5' flanking-promoter			C/T	1	97.9	0.048	0.053	0.043	Y	N	Failed GT assay design
p1048insC (1048_1049)		125348472	Exon 1-5' UTR		insC	[-/C]	1	98.9	0.005	0	**0.011**	Y	Y	
p1257	rs4238001	125348263	Exon 1	Gly2Ser		G/A	0.1595	97.9	0.032	0.032	0.033	Y	N	Failed GT run
p1265	rs2070242	125348255	Exon 1	Ser4Ser		C/T	0.4979	94.7	0.167	0.109	0.227	Y	Y	
p1316	rs10396208	125348204	Exon 1	Cys21Cys		C/T	0.5545	94.7	0.061	0.033	0.091	Y	Y	
p1419	rs201717369	125348101	Intron 1			G/A	1	98.9	0.021	0.021	0.022	Y	Y	
p46964	rs114061302	125302556	Intron 1			G/A	1	100	0.032	0.031	0.032	Y	Y	
p49537	rs7305310	125299983	Intron 2			C/T	0.8226	100	0.095	0.094	0.096	Y	Y	
p49570delC	rs145376237	125299950	Intron 2		delC	[C/-]	0.5967	100	0.242	0.25	0.234	Y	Y	
p49690	rs4765615	125299830	Intron 2			G/A	0.1327	100	0.447	0.365	0.532	Y	Y	
p49759	rs146272788	125299761	Intron 2			C/T	1	100	0.005	**0.01**	0	Y	Y	
p49978	rs5891	125299542	Exon 3	Val135Ile		G/A	1	100	0.011	**0.021**	0	Y	Y	
p50024	rs368880622	125299496	Intron 3			G/T	1	100	0.005	**0.01**	0	Y	Y	
p50118	rs58710319	125299402	Intron 3			C/T	1	100	0.021	0.021	0.021	Y	Y	
p50151	rs2278986	125299369	Intron 3			T/C	1	98.9	0.176	0.223	0.128	Y	Y	
p50380	rs141748317	125299140	Intron 3			A/G	1	100	0.011	0.01	0.011	Y	Y	
p50489	rs61320152	125299031	Intron 3			G/T	1	100	0.021	0.021	0.021	Y	Y	
p50954		125298566	Intron 4			T/C	1	100	0.005	**0.01**	0	Y	Y	
p52919		125296601	Intron 4			G/T	1	100	0.011	0	**0.021**	Y	Y	
p52956	rs77740046	125296564	Intron 4			C/T	1	100	0.047	0.052	0.043	Y	Y	
p52995	rs113910315	125296525	Intron 4-splice site			T/G	1	100	0.005	0	**0.011**	Y	Y	
p53128	rs372212527	125296392	Intron 5			C/T	1	95.8	0.005	**0.011**	0	Y	N	Failed GT run
p53159	rs59809936	125296361	Intron 5			G/A	1	95.8	0.049	0.054	0.044	N		Not selected, tagged with p52956[d]
p53359	rs112371713	125296161	Intron 5			G/A	1	100	0.121	0.156	0.085	Y	Y	
p53372	rs115604379	125296148	Intron 5			C/T	1	100	0.016	**0.031**	0	Y	Y	
p53481	rs143611171	125296039	Intron 5			C/T	0.7877	95.8	0.099	0.109	0.089	Y	N	Failed GT run

SNP Name[a]	SNP ID[b]	Chr12 Position[c]	Location	Amino Acid Change	Indels	Major/ Minor Alleles	HWE *P*	%Call Rate	SEQ MAF			Selection for GT (n = 78)	Success in GT (n = 68)	Note
									Total 95 Subjects	High HDL-C Group (48 subjects)	Low HDL-C Group (47 subjects)			
p54445	rs60910935	125295075	Intron 5			A/G	1	100	0.032	0.021	0.043	Y	Y	
p54475	rs60227139	125295045	Intron 5			C/T	1	100	0.032	0.021	0.043	Y	Y	
p54492	rs61762481	125295028	Intron 5			G/A	0.5776	100	0.111	0.062	0.16	Y	Y	
p54611		125294909	Intron 5			T/C	1	100	0.005	0	**0.011**	Y	Y	
p54627		125294893	Intron 5			G/C	1	100	0.005	**0.01**	0	Y	Y	
p54856		125294664	Intron 6			C/T	1	100	0.005	0	**0.011**	Y	Y	
p56845	rs838902	125292675	Intron 6			A/G	0.6252	98.9	0.41	0.385	0.435	Y	Y	
p57004	rs187562853	125292516	Intron 6			G/A	1	100	0.021	**0.042**	0	Y	Y	
p57107	rs5892	125292413	Exon 7	Phe301Phe		C/T	1	100	0.026	0.031	0.021	Y	Y	
p57508	rs71458866	125292012	Intron 7			G/A	0.646	98.9	0.106	0.073	0.141	Y	Y	
p57592	rs838903	125291928	Intron 7			G/A	0.1308	98.9	0.356	0.323	0.391	Y	Y	
p64772	rs5888	125284748	Exon 8	Ala350Ala		C/T	1	100	0.116	0.125	0.106	Y	Y	
p64923	rs838915	125284597	Intron 8			C/A	0.2751	100	0.137	0.156	0.117	Y	Y	
p69699	rs10396210	125279821	Intron 8-splice site			G/A	1	98.9	0.138	0.096	0.181	Y	Y	
p69994	rs188094006	125279526	Intron 9			C/T	1	100	0.005	**0.01**	0	Y	N	Failed GT run
p69995delC	rs5801571	125279525	Intron 9		delC	[C/-]	0.7558	100	0.253	0.25	0.255	Y	Y	
p70088	rs117585141	125279432	Intron 9			G/A	1	100	0.158	0.125	0.191	Y	N	Failed GT run
p70129	rs1814575	125279391	Intron 9			C/T	1	100	0.047	0.052	0.043	Y	N	Failed GT run
p70148	rs183061101	125279372	Intron 9			T/C	1	100	0.011	0.01	0.011	Y	N	Failed GT run
p70201		125279319	Intron 9			T/C	1	100	0.005	**0.01**	0	Y	Y	
p70292	rs150388176	125279228	Intron 9			C/T	1	98.9	0.016	0	**0.033**	Y	N	Failed GT assay design
p77181	rs146246031	125272339	Intron 9			T/C	1	98.9	0.005	**0.01**	0	Y	Y	
p77250	rs201901986	125272270	Intron 9			G/T	0.8226	100	0.095	0.052	0.138	N		Not selected, tagged with p77251[d]
p77251	rs34339961	125272269	Intron 9			A/T	0.8954	97.9	0.091	0.043	0.138	Y	Y	
p77381	rs138499966	125272139	Intron 9			T/C	1	100	0.005	**0.01**	0	Y	Y	
p77620	rs377124254	125271900	Intron 10			G/A	1	100	0.005	**0.01**	0	Y	Y	
p77682	rs150082885	125271838	Intron 10			A/G	0.0317	100	0.016	0.01	0.021	Y	Y	
p77704		125271816	Intron 10			C/A	1	100	0.005	**0.01**	0	Y	Y	
p77842	rs2272310	125271678	Intron 10			G/A	1	100	0.084	0.094	0.074	Y	Y	
p78255	rs184052375	125271265	Intron 10			A/G	1	100	0.016	0.021	0.011	Y	Y	
p78402	rs838898	125271118	Intron 10			G/A	1	100	0.063	0.073	0.053	Y	Y	
p78430	rs838897	125271090	Intron 10			C/G	0.3016	94.7	0.356	0.344	0.367	Y	Y	
p78747	rs2293440	125270773	Intron 11			T/C	0.8126	96.8	0.315	0.244	0.383	Y	Y	

SNP Name[a]	SNP ID[b]	Chr12 Position[c]	Location	Amino Acid Change	Indels	Major/ Minor Alleles	HWE *P*	%Call Rate	SEQ MAF			Selection for GT (n = 78)	Success in GT (n = 68)	Note
									Total 95 Subjects	High HDL-C Group (48 subjects)	Low HDL-C Group (47 subjects)			
p78791	rs75289200	125270729	Intron 11			T/C	1	98.9	0.043	0.043	0.043	Y	Y	
p81863	rs185445624	125267657	Intron 11			G/A	1	100	0.005	0	**0.011**	Y	Y	
p82019	rs838890	125267501	Intron 11			C/T	1	100	0.032	0.031	0.032	Y	Y	
p82264	rs141545424	125267256	Exon 12	Gly501Gly		C/A	1	100	0.005	**0.01**	0	Y	Y	
p82340	rs77483223	125267180	Intron 12			G/A	1	100	0.005	0	**0.011**	Y	Y	
p82369	rs75446635	125267151	Intron 12			G/A	1	100	0.032	0.042	0.021	Y	Y	
p82434	rs838889	125267086	Intron 12			T/C	1	100	0.032	0.031	0.032	Y	Y	
p86245	rs188375019	125263275	Intron 12			C/T	1	98.9	0.032	0.031	0.033	Y	Y	
p86276	rs747155	125263244	Intron 12			C/T	0.8101	98.9	0.122	0.16	0.085	Y	Y	
p86316	rs701104	125263204	Intron 12			G/T	1	100	0.032	0.021	0.043	Y	Y	
p86481	rs701103	125263039	Exon 13- 3' UTR	Gly499Arg (isoform 2)		G/A	0.4173	97.9	0.237	0.298	0.174	Y	Y	
p86967	rs187492239	125262553	Exon 13- 3' UTR			A/G	1	97.9	0.032	0.032	0.033	Y	Y	
p87011	rs58032386	125262509	Exon 13- 3' UTR			C/T	0.3196	100	0.126	0.104	0.149	Y	Y	
p87195	rs10396211	125262325	Exon 13- 3' UTR			G/C	0.8101	98.9	0.122	0.094	0.152	N		Not selected, tagged with p87011[d]
p87266	rs150512235	125262254	Exon 13- 3' UTR[e]			T/C	1	100	0.032	0.042	0.021	Y	Y	
p87416	rs838884	125262104	3' flanking			C/T	0.6922	97.9	0.301	0.323	0.278	N		Not selected, tagged with p87723[d]
p87459		125262061	3' flanking			C/T	1	98.9	0.005	**0.01**	0	Y	N	Failed GT run
p87611	rs190688220	125261909	3' flanking			C/T	1	100	0.032	0.031	0.032	Y	Y	
p87681	rs838883	125261839	3' flanking			G/A	1	100	0.032	0.021	0.043	Y	Y	
p87694		125261826	3' flanking			C/T	1	100	0.005	**0.01**	0	Y	Y	
p87707	rs838882	125261813	3' flanking			G/A	0.9969	98.9	0.303	0.333	0.272	N		Not selected, tagged with p87723[d]
p87723	rs838881	125261797	3' flanking			C/T	0.6922	97.9	0.301	0.326	0.277	Y	Y	
p87749	rs76465225	125261771	3' flanking			G/A	0.3707	100	0.095	0.094	0.096	Y	Y	
p87927	rs838880	125261593	3' flanking			G/A	1	100	0.226	0.25	0.202	Y	Y	

del, deletion; GT, genotyping; HDL-C, high-density lipoprotein cholesterol; HWE; Hardy-Weinberg equilibrium; Indels, insertion and deletion variations; ins, insertion; MAF, minor allele frequency; SEQ, sequencing; SNP, single nucleotide polymorphism; UTR, untranslated region. N, no; Y, yes.
All alleles on the reverse strand. Splice site is defined as ± 20 bp from the start or end of an exon.
All 10 novel variants identified in this study have been submitted to dbSNP database (batch ID: SCARB1_AB): http://www.ncbi.nlm.nih.gov/SNP/snp_viewTable.cgi?handle=KAMBOH.
MAFs of the variants that were specifically found in only one extreme HDL-C group are shown in **bold**.
[a, c] RefSeq of *SCARB1*: hg19, NM_005505 (CHIP Bioinformatics).
[b] dbSNP build 139: GRCh37.p10.
[d] LD plots of all 83 sequence variants and the 32 sequence variants with MAF ≥5% are shown in Additional file 5 Figure S1 and Additional file 6 Figure S2, respectively.
[e] Close to a miRNA-145 seed site based on TargetScanHuman (version 6.2, http://www.targetscan.org/).
