# Supplementary material for: Genetic contribution of SCARB1 variants to lipid traits in African Blacks: a candidate gene association study
Source: BMC Med Genet. 2015 Nov 12;16:106. doi: 10.1186/s12881-015-0250-6 (PMC4643515; doi:10.1186/s12881-015-0250-6)
Supplement: Additional file 4: Table S4. — Characteristics of 10 SCARB1 novel variants identified by sequencing. (PDF 81 kb) [file 12881_2015_250_MOESM4_ESM.pdf]

Table S4. Characteristics of 10 *SCARB1* novel[a] variants identified by sequencing.

SNP Name[b]	Chr12 Position[c]	Location	RegulomeDB Score[d]	Major/Minor Alleles	MAF	MA Effect on Traits[e]
p1048insC (1048_1049)	125348472	Exon 1-5' UTR	2a	[-/C]	0.0079	
p50954	125298566	Intron 4	5	T/C	0.0007	
p52919	125296601	Intron 4	5	G/T	0.0013	↓ HDL-C, ↓ ApoA-I
p54611	125294909	Intron 5	4	T/C	0.0007	↓ HDL-C, ↓ ApoA-I
p54627	125294893	Intron 5	4	G/C	0.0020	
p54856	125294664	Intron 6	4	C/T	0.0007	↓ HDL-C, ↓ ApoA-I
p70201[f]	125279319	Intron 9	6	T/C	0.0010	
p77704	125271816	Intron 10	5	C/A	0.0040	
p87459	125262061	3' flanking	4	C/T	0.0050[g]	
p87694	125261826	3' flanking	5	C/T	0.0020	

ApoA-I, apolipoprotein A-I; HDL-C, high-density lipoprotein cholesterol; ins, insertion; MA, minor allele; MAF, minor allele frequency; SNP; single nucleotide polymorphism; UTR, untranslated region. ↓, decreased.
All alleles on the reverse strand.
All 10 novel variants identified in this study have been submitted to dbSNP database (batch ID: SCARB1_AB): http://www.ncbi.nlm.nih.gov/SNP/snp_viewTable.cgi?handle=KAMBOH.
[a] dbSNP build 139: GRCh37.p10.
[b, c] RefSeq of *SCARB1*: hg19, NM_005505 (CHIP Bioinformatics).
[d] The RegulomeDB (version 1.0) scoring scheme: **score 1a**, expression quantitative trait loci (eQTL) + transcription factor (TF) binding + matched TF motif + matched DNase Footprint + DNase peak; **score 1b**, eQTL + TF binding + any motif + DNase Footprint + DNase peak; **score 1c**, eQTL + TF binding + matched TF motif + DNase peak; **score 1d**, eQTL + TF binding + any motif + DNase peak; **score 1e**, eQTL + TF binding + matched TF motif; **score 1f**, eQTL + TF binding / DNase peak, **score 2a**, TF binding + matched TF motif + matched DNase Footprint + DNase peak; **score 2b**, TF binding + any motif + DNase Footprint + DNase peak; **score 2c**, TF binding + matched TF motif + DNase peak; **score 3a**, TF binding + any motif + DNase peak; **score 3b**, TF binding + matched TF motif; **score 4**,TF binding + DNase peak; **score 5**, TF binding or DNase peak; **score 6**, others; **score 7**, no data (see http://regulome.stanford.edu/help). See detailed functional assignments in Additional file 19 Table S13.
[e] Evidence of association is based on single-site analysis; see details in Additional file 14 Table S9 and Additional file 15 Table S10.
[f] p70201/chr12:125279319 was excluded from gene-based, single-site, and haplotype analyses, but included in rare variant analysis; see details in Additional file 9 Table S6 and Figure 1.
[g] MAF based on sequencing data/sample due to genotyping failure.
