# Supplementary figures and images for: Genetic contribution of SCARB1 variants to lipid traits in African Blacks: a candidate gene association study
Source: BMC Med Genet. 2015 Nov 12;16:106. doi: 10.1186/s12881-015-0250-6 (PMC4643515; doi:10.1186/s12881-015-0250-6)

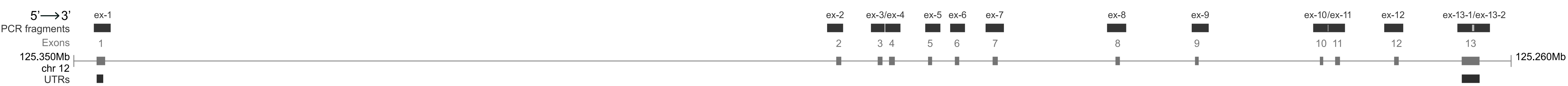

LD structure by sequencing

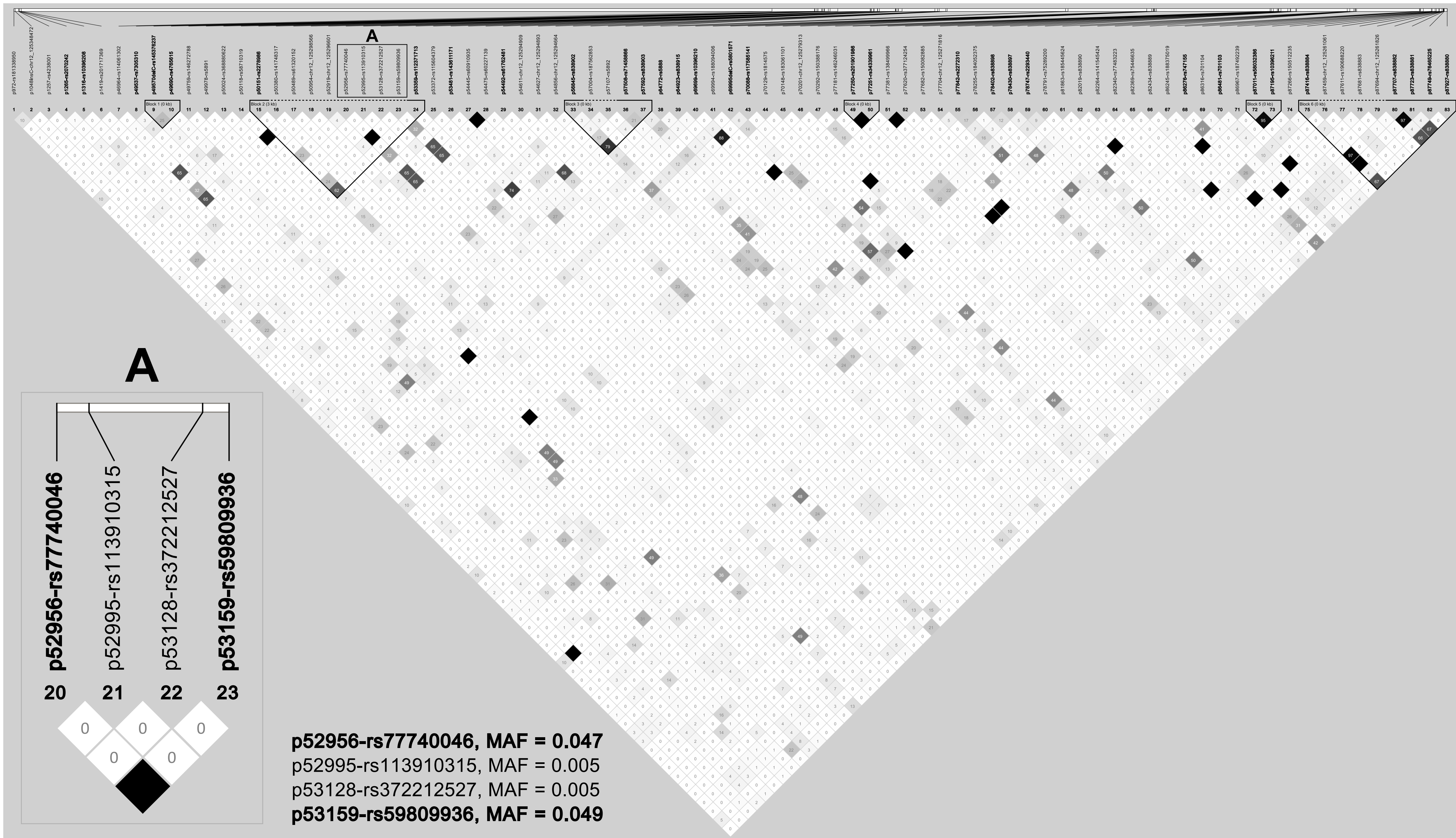

Supplement: Additional file 5: Figure S1. — Linkage disequilibrium (LD) plot of 83 SCARB1 sequence variants. Of 83 sequence variants (see the list in Additional file 3: Table S3), 78 were selected for genotyping. An enlarged view of the part of LD plot (A) shows the pairwise correlations (r2) between four variants including the two variants (shown in bold) in the same bin in our data, of which one selected for genotyping. This bin was not identified by Tagger analysis of common SCARB1 variants in the HapMap-YRI data (see Additional file 7: Table S5 and Additional file 8: Figure S3). The degree of shades and values (r2 × 100) in each square of LD plot represent the pairwise correlations: black indicating r2 = 1, white indicating r2 = 0, and shade intensity indicating r2 between 0 and 1. LD, linkage disequilibrium; MAF, minor allele frequency; YRI, Yoruba people of Ibadan from Nigeria. (PDF 920 kb) [file 12881_2015_250_MOESM5_ESM.pdf]

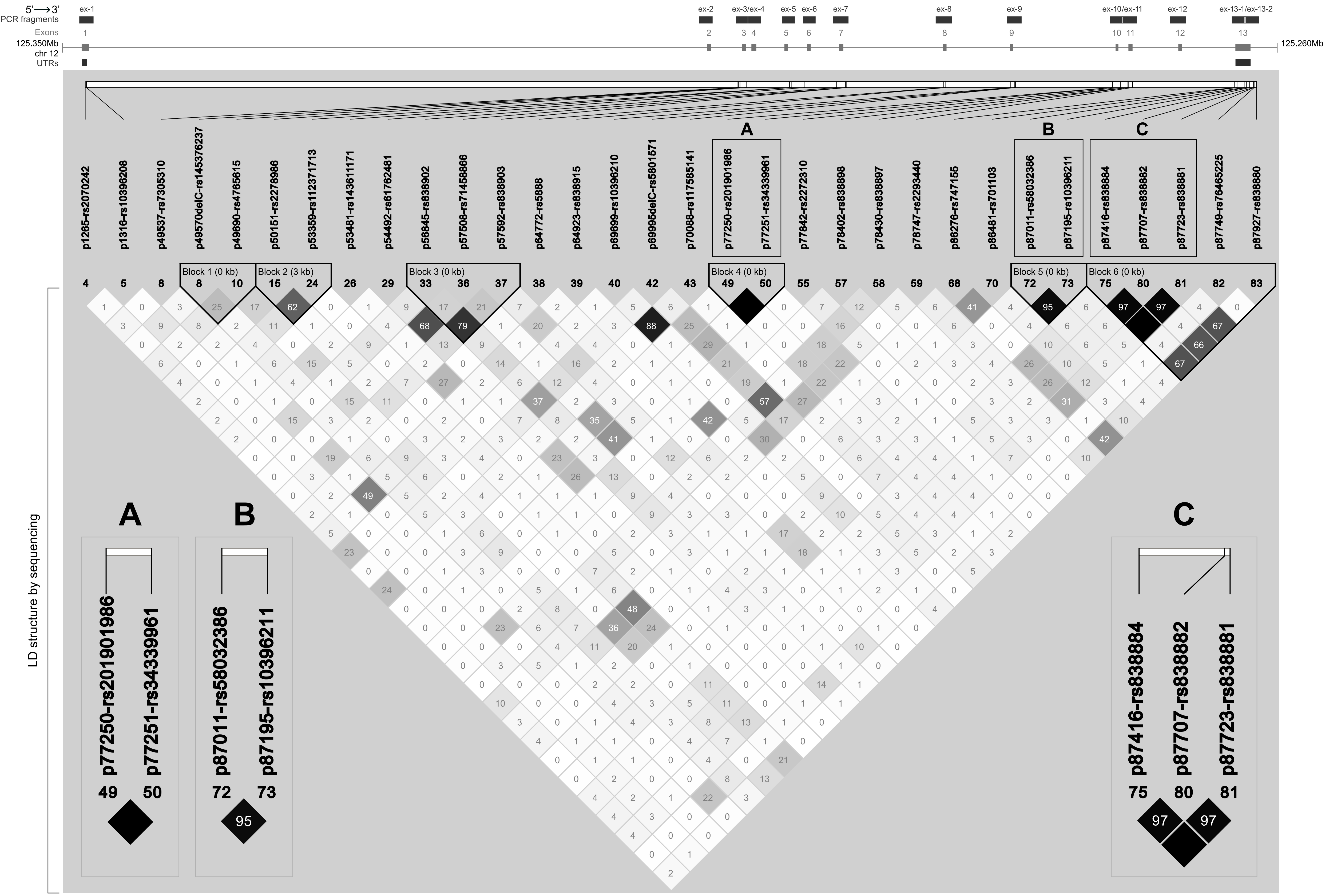

Supplement: Additional file 6: Figure S2. — Linkage disequilibrium (LD) plot of 32 SCARB1 common sequence variants. Enlarged view of the parts of the LD plot (A, B, and C) show three LD bins (identified by Tagger analysis of variants with minor allele frequency (MAF) ≥5 % using an r2 cutoff of 0.90) containing more than one variant (r2 ranging between 0.95 and 1.0). The degree of shades and values (r2 × 100) in each square of LD plot represent the pairwise correlations: black indicating r2 = 1, white indicating r2 = 0, and shade intensity indicating r2 between 0 and 1. LD, linkage disequilibrium; MAF, minor allele frequency. (PDF 370 kb) [file 12881_2015_250_MOESM6_ESM.pdf]

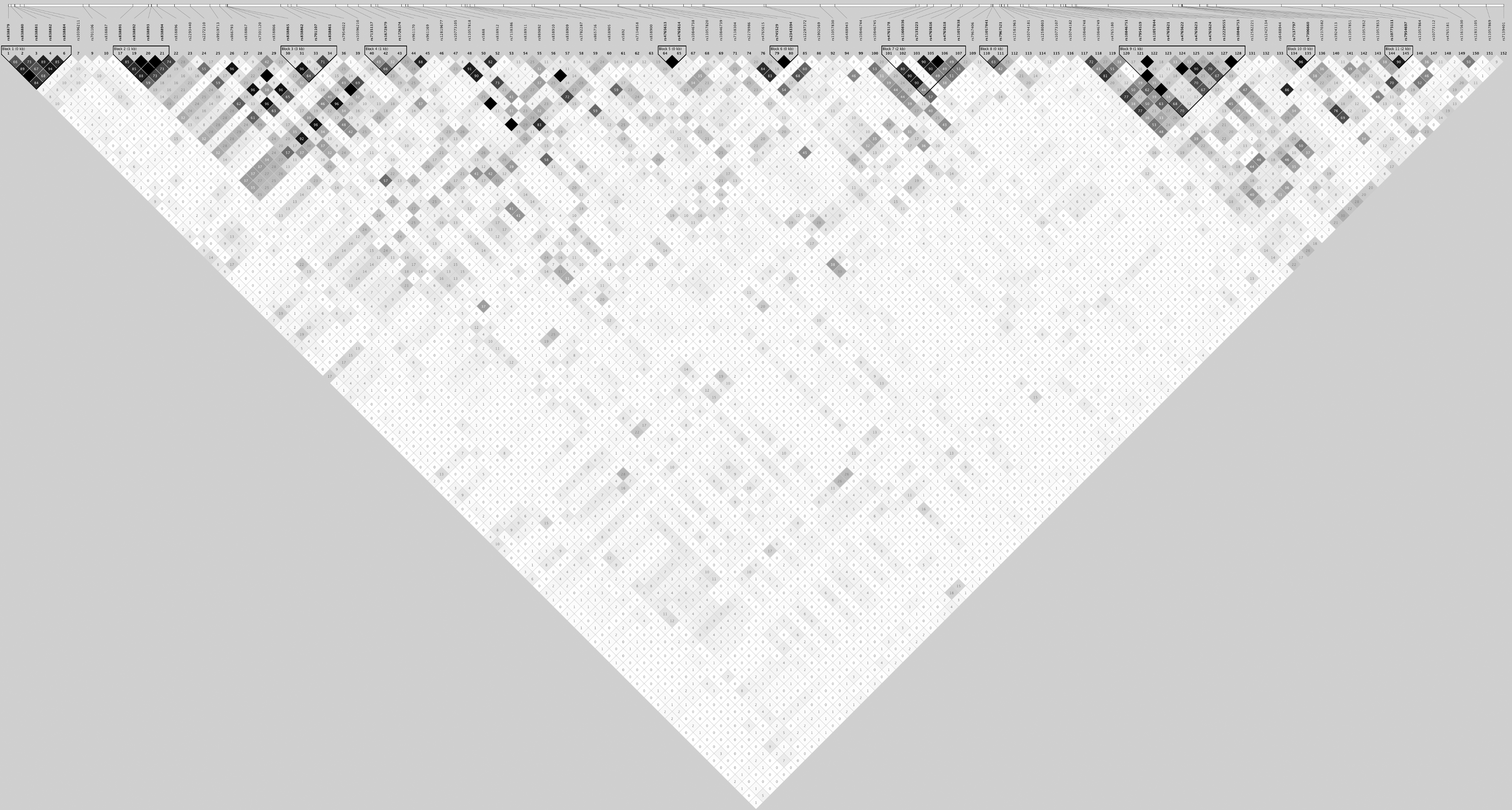

Supplement: Additional file 8: Figure S3. — Linkage disequilibrium (LD) plot of 108 SCARB1 common HapMap-YRI tagSNPs. The list of 77 common HapMap-YRI tagSNPs identified by Tagger analysis of variants with minor allele frequency ≥5 % using an r2 cutoff of 0.80 is shown in Additional file 7: Table S5. The degree of shades and values (r2 × 100) in each square of LD plot represent the pairwise correlations: black indicating r2 = 1, white indicating r2 = 0, and shade intensity indicating r2 between 0 and 1.LD, linkage disequilibrium; SNP, single nucleotide polymorphism; YRI, Yoruba people of Ibadan from Nigeria. (TIFF 2642 kb) [file 12881_2015_250_MOESM8_ESM.tiff]

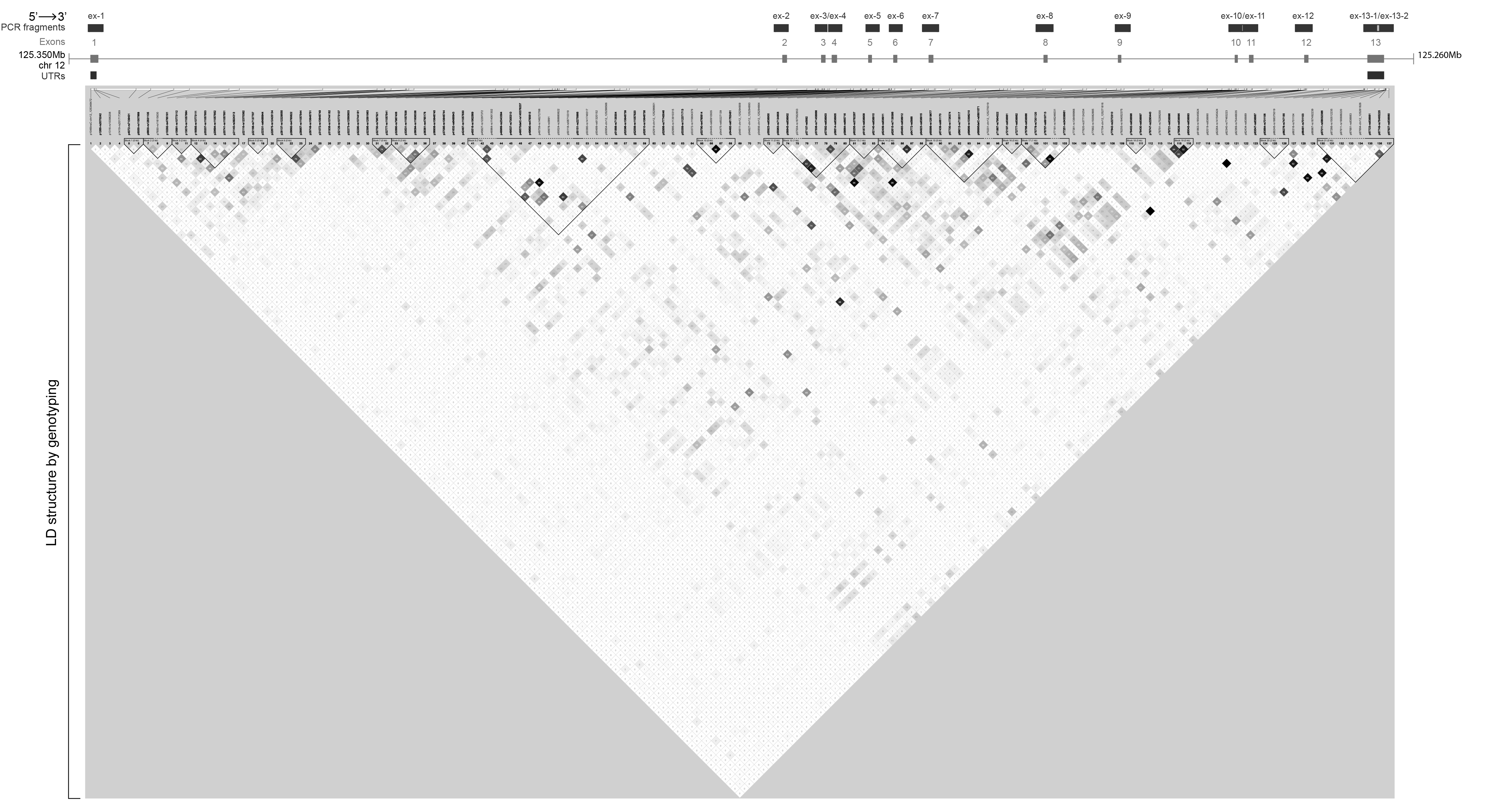

Supplement: Additional file 11: Figure S4. — Linkage disequilibrium (LD) plot of 137 SCARB1 genotyped variants. The list of 87 genotyped common tagSNPs identified by Tagger analysis for variants with minor allele frequency ≥5 % using an r2 cutoff of 0.90 is shown in Additional file 10: Table S7. The degree of shades and values (r2 × 100) in each square of LD plot represent the pairwise correlations: black indicating r2 = 1, white indicating r2 = 0, and shade intensity indicating r2 between 0 and 1.LD, linkage disequilibrium; SNP, single nucleotide polymorphism. (TIFF 3095 kb) [file 12881_2015_250_MOESM11_ESM.tiff]

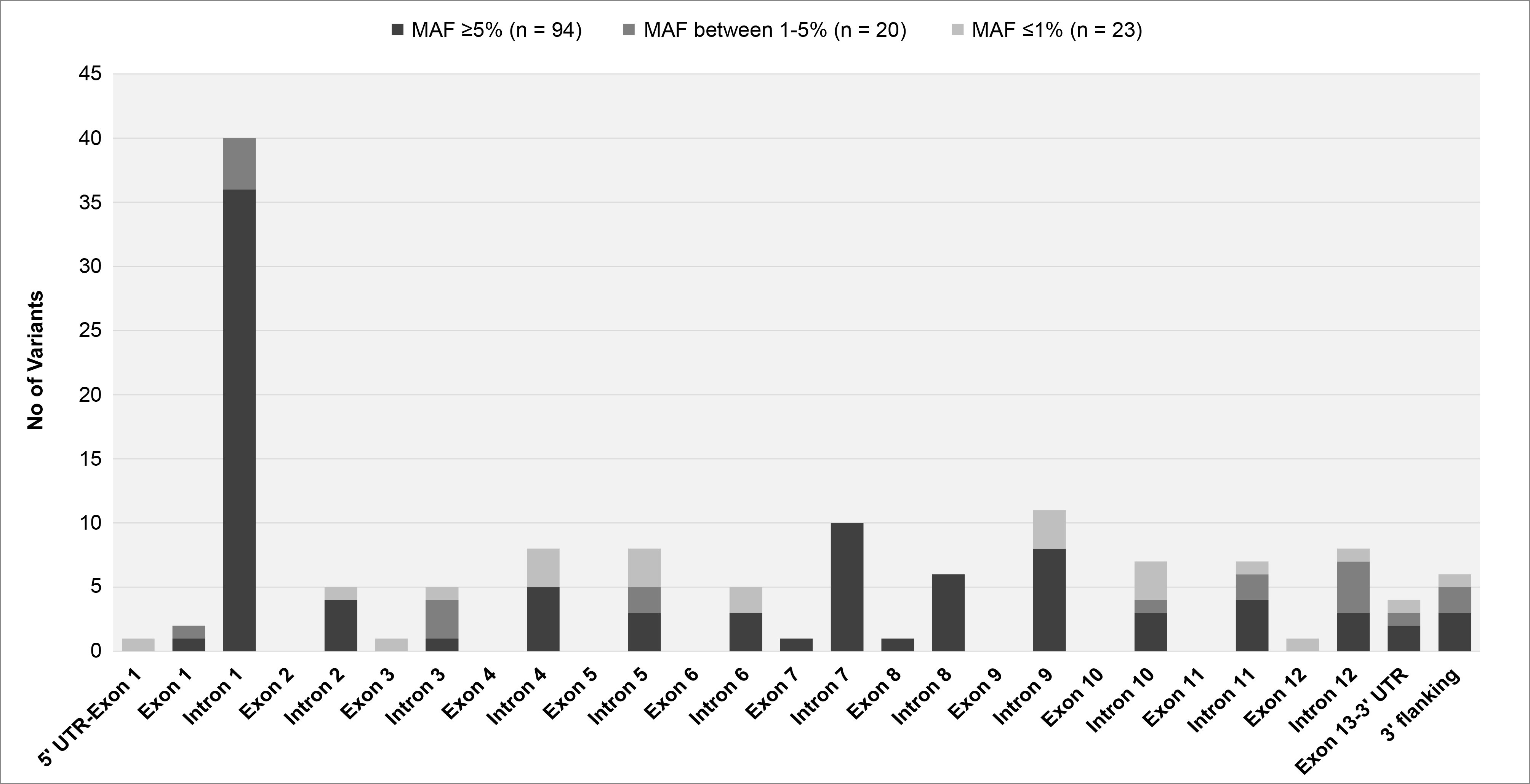

Supplement: Additional file 12: Figure S5. — Location and minor allele frequency (MAF) distributions of 137 SCARB1 genotyped variants. Details for each variant are shown in Additional file 9: Table S6. MAF, minor allele frequency; UTR, untranslated region. (TIFF 324 kb) [file 12881_2015_250_MOESM12_ESM.tiff]
