# Supplementary material for: Genetic contribution of SCARB1 variants to lipid traits in African Blacks: a candidate gene association study
Source: BMC Med Genet. 2015 Nov 12;16:106. doi: 10.1186/s12881-015-0250-6 (PMC4643515; doi:10.1186/s12881-015-0250-6)
Supplement: Additional file 7: Table S5. — List of 77 SCARB1 HapMap-YRI tagSNPs. (PDF 110 kb) [file 12881_2015_250_MOESM7_ESM.pdf]

Table S5. List of 77 *SCARB1* HapMap[a]-YRI tagSNPs (using Tagger analysis for variants with MAF ≥5% and r^2 ≥0.80, capturing a total of 108 common HapMap-YRI tagSNPs with a mean r^2 of 0.98; see LD plot in Additional file 8 Figure S3).

Bin #	TagSNP	Alleles Captured
1	rs9919713	rs838867,rs838865,rs1672879,rs701107
2	rs838893	rs838892,rs838894,rs838891
3	rs4765178	rs4765616,rs4765618,rs7135223
4	**rs838881**	rs838882,rs838879,rs838884
5	rs10846751	rs4765623,rs10846748,rs4765621
6	rs2343394	rs2278986, rs745529
7	rs838912[b]	rs5888[b],rs10773105
8	rs12819677	rs989892,rs3782287
9	rs7137797	rs7306660
10	rs4765614	rs4765613
11	rs10773111	rs7954697
12	rs12370382	rs12582221
13	rs11057844	rs7954519
14	rs838905	rs11057818
15	rs12229555	rs10846753
16	rs4765622	rs4765624
17	rs961170	rs961169
18	rs3924313	
19	rs838866	
20	rs10846744	
21	rs10744181	
22	rs4765181	
23	rs7135117	
24	rs7138304	
25	rs838887	
26	rs12581963	
27	rs11057853[b]	
28	rs11608336	
29	rs10846749	
30	rs838896[b]	
31	rs6488944	
32	rs10773112	
33	**rs4765615**[b]	
34	rs11057869	
35	rs7954022	
36	rs11057852	
37	rs6488943	
38	rs11057820	
39	rs10744182	
40	rs838861	
41	rs10846745	
42	rs838911	

43	rs4765180[e]	
44	**rs10396210**	
45	rs838862	
46	rs7967521	
47	**rs2272310**	
48	rs865716	
49	rs12297372	
50	rs7134858[b]	
51	rs7139401	
52	rs1726374	
53	rs12580803	
54	rs701106[b]	
55	rs838909	
56	rs11057864	
57	**rs5892**	
58	rs10846739	
59	**rs838880**	
60	rs866793[d]	
61	**rs2293440**	
62	rs10846738	
63	rs838910	
64	rs12425134	
65	rs838900	
66	rs1902569[b]	
67	rs10773107	
68	rs10396211[c]	
69	rs11615630	
70	rs7301120	
71	rs11057838	
72	rs7967406	
73	rs7138386	
74	rs12831105	
75	rs11057830	
76	rs11057841	
77	rs11057851[b]	

LD, linkage disequilibrium; MAF, minor allele frequency; SNP, single nucleotide polymorphism; YRI, Yoruba people of Ibadan, Nigeria.

Of 77 common HapMap-YRI tagSNPs, 69 HapMap-YRI tagSNPs were selected for genotyping in addition to seven tagSNPs that were already selected (Additional file 3 Table S3) based on our sequencing data (shown in **bold**), and one was tagged by another already selected sequence variant in our data (see below under “c”).

Of theses 69 common HapMap tagSNPs selected for genotyping, 68 were successfully genotyped, and 67 were advance into the downstream analyses (Additional file 9 Table S6); see below under “d” the variant that was excluded from the downstream analyses as it failed quality control checks, and see below under “e” the variant that failed genotyping.

[a] HapMap release #27: NCBI build 36, dbSNP version 126.

[b] SNPs with nominally significant evidence of single-site association with either high-density lipoprotein cholesterol or apolipoprotein A-I (*P* <0.05; Additional file 14 Table S9 and Additional file 15 Table S10) observed in the current study.

[c] rs10396211 was not selected for genotyping. rs10396211 was tagged by one SNP identified by sequencing, rs58032386 (r^2 = 0.95), in our sample (Enlarged view B in Additional file 6 Figure S2).

[d] rs866793 deviated from Hardy-Weinberg equilibrium and was excluded from the downstream analyses.

[e] rs4765180 failed genotyping and was excluded from the downstream analyses.
