# Supplementary material for: Genetic contribution of SCARB1 variants to lipid traits in African Blacks: a candidate gene association study
Source: BMC Med Genet. 2015 Nov 12;16:106. doi: 10.1186/s12881-015-0250-6 (PMC4643515; doi:10.1186/s12881-015-0250-6)
Supplement: Additional file 9: Table S6. — Characteristics of 138 SCARB1 variants genotyped in the entire sample of 788 African Blacks. (PDF 212 kb) [file 12881_2015_250_MOESM9_ESM.pdf]

Table S6. Characteristics of 138 *SCARB1* variants genotyped in the entire sample of 788 African Blacks.

SNP Name[a]	SNP ID[b]	Chr12 Position[c]	Location	Amino acid Change	Major/Minor Alleles	HWE *P*	%Call Rate	MAF	Source of Variants	Success in GT (n = 138)	Proceed to Analyses (n = 137)	Note
p1048insC (1048_1049)		125348472	Exon 1- 5' UTR		[-/C]	1	96.1	0.0079	SEQ	Y	Y	
p1265	rs2070242	125348255	Exon 1	Ser4Ser	C/T	0.8725	93.4	0.1284	SEQ	Y	Y	
p1316	rs10396208	125348204	Exon 1	Cys21Cys	C/T	0.1503	93.4	0.0476	SEQ	Y	Y	
p1419	rs201717369	125348101	Intron 1		G/A	0.1976	94.4	0.0121	SEQ	Y	Y	
p4072	rs7139401	125345448	Intron 1		T/C	0.331	95.1	0.4386	HapMap	Y	Y	
p5055	rs11057869	125344465	Intron 1		G/A	0.0974	97.7	0.0740	HapMap	Y	Y	
p6600	rs12831105	125342920	Intron 1		C/T	0.979	95.1	0.1188	HapMap	Y	Y	
p7650	rs11615630	125341870	Intron 1		G/A	0.4345	99	0.0436	HapMap	Y	Y	
p10292	rs4765181	125339228	Intron 1		G/T	0.6396	98.4	0.2490	HapMap	Y	Y	
p10991	rs10773112	125338529	Intron 1		G/A	0.8029	95.7	0.3534	HapMap	Y	Y	
p13570	rs11057864	125335950	Intron 1		G/T	0.193	97.8	0.1180	HapMap	Y	Y	
p16565	rs10773111	125332955	Intron 1		G/A	0.3383	98.1	0.1928	HapMap	Y	Y	
p20207	rs11057853	125329313	Intron 1		G/A	0.7053	97.2	0.4484	HapMap	Y	Y	
p20694	rs11057852	125328826	Intron 1		G/A	0.3519	94.3	0.1050	HapMap	Y	Y	
p20741	rs11057851	125328779	Intron 1		C/T	0.4728	96.8	0.3237	HapMap	Y	Y	
p21145	rs3924313	125328375	Intron 1		C/T	1	98.1	0.1772	HapMap	Y	Y	
p22116	rs12370382	125327404	Intron 1		G/A	1	96.4	0.0645	HapMap	Y	Y	
p22168	rs7137797	125327352	Intron 1		T/C	0.6804	94.9	0.3977	HapMap	Y	Y	
p22331	rs6488944	125327189	Intron 1		T/G	0.2943	94.3	0.1474	HapMap	Y	Y	
p22675	rs12425134	125326845	Intron 1		G/T	1	96.4	0.0526	HapMap	Y	Y	
p28137	rs12229555	125321383	Intron 1		A/G	0.0888	95.9	0.3896	HapMap	Y	Y	
p28692	rs4765622	125320828	Intron 1		C/T	0.2025	97	0.2565	HapMap	Y	Y	
p28957	rs11057844	125320563	Intron 1		G/A	0.9612	96.4	0.2362	HapMap	Y	Y	
p29749	rs10846751	125319771	Intron 1		C/T	0.6254	93.7	0.4492	HapMap	Y	Y	
p31072	rs10846749	125318448	Intron 1		C/G	0.9027	96.6	0.4461	HapMap	Y	Y	
p31938	rs10744182	125317582	Intron 1		G/A	0.9847	96.7	0.1837	HapMap	Y	Y	
p32129	rs10773107	125317391	Intron 1		G/T	0.7277	96.8	0.1009	HapMap	Y	Y	
p32273	rs12580803	125317247	Intron 1		T/C	2.00E-04	99	0.1006	HapMap	Y	Y	
p32290	rs10744181	125317230	Intron 1		T/C	0.0356	92.3	0.1238	HapMap	Y	Y	
p32395	rs12581963	125317125	Intron 1		C/T	0.5734	98.5	0.1314	HapMap	Y	Y	
p32750	rs7967521	125316770	Intron 1		A/G	0.2715	91.9	0.3425	HapMap	Y	Y	

SNP Name[a]	SNP ID[b]	Chr12 Position[c]	Location	Amino acid Change	Major/Minor Alleles	HWE *P*	%Call Rate	MAF	Source of Variants	Success in GT (n = 138)	Proceed to Analyses (n = 137)	Note
p32777	rs11057841	125316743	Intron 1		G/A	0.3613	94.5	0.2805	HapMap	Y	Y	
p32860	rs7967406	125316660	Intron 1		A/C	0.6852	98.6	0.0991	HapMap	Y	Y	
p33531	rs11057838	125315989	Intron 1		C/A	0.7642	95.8	0.2278	HapMap	Y	Y	
p36094	rs11608336	125313426	Intron 1		G/A	0.7249	95.4	0.1543	HapMap	Y	Y	
p36461	rs4765178	125313059	Intron 1		C/T	0.8082	93.4	0.1671	HapMap	Y	Y	
p36908	rs10846745	125312612	Intron 1		C/G	0.6639	97.2	0.3257	HapMap	Y	Y	
p37095	rs10846744	125312425	Intron 1		C/G	0.0194	98.2	0.3056	HapMap	Y	Y	
p41632	rs6488943	125307888	Intron 1		A/C	0.0626	91.5	0.2954	HapMap	Y	Y	
p42467	rs11057830	125307053	Intron 1		C/T	0.3753	97.1	0.1523	HapMap	Y	Y	
p45516	rs1902569	125304004	Intron 1		G/A	1	94.9	0.1544	HapMap	Y	Y	
p45627	rs12297372	125303893	Intron 1		A/G	0.9301	95.1	0.0487	HapMap	Y	Y	
p46964	rs114061302	125302556	Intron 1		G/A	1	98.1	0.0388	SEQ	Y	Y	
p48969	rs2343394	125300551	Intron 2		C/T	0.401	98.6	0.1898	HapMap	Y	Y	
p49537	rs7305310	125299983	Intron 2		C/T	0.0033	95.2	0.1007	SEQ	Y	Y	
p49570delC	rs145376237	125299950	Intron 2		[C/-]	0.8066	95.1	0.2276	SEQ	Y	Y	
p49690	rs4765615	125299830	Intron 2		G/A	0.0084	93.9	0.4426	SEQ	Y	Y	
p49759	rs146272788	125299761	Intron 2		C/T	1	95.2	0.0020	SEQ	Y	Y	
p49978	rs5891	125299542	Exon 3	Val135Ile	G/A	1	99.1	0.0058	SEQ	Y	Y	
p50024	rs368880622	125299496	Intron 3		G/T	1	96.7	0.0026	SEQ	Y	Y	
p50118	rs58710319	125299402	Intron 3		C/T	1	97.6	0.0208	SEQ	Y	Y	
p50151	rs2278986	125299369	Intron 3		T/C	0.3798	97.8	0.1933	SEQ	Y	Y	
p50380	rs141748317	125299140	Intron 3		A/G	1	96.4	0.0112	SEQ	Y	Y	
p50489	rs61320152	125299031	Intron 3		G/T	1	96.3	0.0257	SEQ	Y	Y	
p50954		125298566	Intron 4		T/C	1	96.2	0.0007	SEQ	Y	Y	
p51888	rs7138304	125297632	Intron 4		C/T	0.0164	95.8	0.1079	HapMap	Y	Y	
p52096	rs10846739	125297424	Intron 4		A/G	0.0939	95.2	0.4693	HapMap	Y	Y	
p52556	rs11057820	125296964	Intron 4		G/A	0.1399	98.4	0.1000	HapMap	Y	Y	
p52610	rs10846738	125296910	Intron 4		C/T	0.9717	95.9	0.1349	HapMap	Y	Y	
p52919		125296601	Intron 4		G/T	1	96.2	0.0013	SEQ	Y	Y	
p52956	rs77740046	125296564	Intron 4		C/T	0.7667	95.3	0.0546	SEQ	Y	Y	
p52995	rs113910315	125296525	Intron 4-splice site		T/G	1	97.1	0.0020	SEQ	Y	Y	
p53359	rs112371713	125296161	Intron 5		G/A	0.7881	93.9	0.1243	SEQ	Y	Y	
p53372	rs115604379	125296148	Intron 5		C/T	1	96.6	0.0066	SEQ	Y	Y	
p53790	rs4765614	125295730	Intron 5		G/A	0.2998	95.7	0.2653	HapMap	Y	Y	
p54445	rs60910935	125295075	Intron 5		A/G	0.7209	92.6	0.0418	SEQ	Y	Y	

SNP Name[a]	SNP ID[b]	Chr12 Position[c]	Location	Amino acid Change	Major/Minor Alleles	HWE *P*	%Call Rate	MAF	Source of Variants	Success in GT (n = 138)	Proceed to Analyses (n = 137)	Note
p54475	rs60227139	125295045	Intron 5		C/T	0.8434	95.8	0.0437	SEQ	Y	Y	
p54492	rs61762481	125295028	Intron 5		G/A	1	99.1	0.1005	SEQ	Y	Y	
p54611		125294909	Intron 5		T/C	1	97.1	0.0007	SEQ	Y	Y	
p54627		125294893	Intron 5		G/C	1	97	0.0020	SEQ	Y	Y	
p54856		125294664	Intron 6		C/T	1	97.1	0.0007	SEQ	Y	Y	
p55923	rs838900	125293597	Intron 6		G/A	0.6724	96.4	0.3921	HapMap	Y	Y	
p55963	rs7134858	125293557	Intron 6		C/T	0.189	97.6	0.1560	HapMap	Y	Y	
p56845	rs838902	125292675	Intron 6		A/G	0.2799	97.2	0.4249	SEQ	Y	Y	
p57004	rs187562853	125292516	Intron 6		G/A	1	97	0.0098	SEQ	Y	Y	
p57107	rs5892	125292413	Exon 7	Phe301Phe	C/T	0.4893	97	0.0589	SEQ	Y	Y	
p57508	rs71458866	125292012	Intron 7		G/A	0.3467	98.9	0.1130	SEQ	Y	Y	
p57592	rs838903	125291928	Intron 7		G/A	0.3215	97	0.3763	SEQ	Y	Y	
p58514	rs838905	125291006	Intron 7		T/C	0.2442	97.3	0.4329	HapMap	Y	Y	
p58664	rs865716	125290856	Intron 7		A/T	0.1006	94.7	0.2708	HapMap	Y	Y	
p60255	rs3782287	125289265	Intron 7		C/T	0.4661	97.7	0.2831	Literature	Y	Y	
p61872	rs838909	125287648	Intron 7		C/T	0.9762	95.8	0.2199	HapMap	Y	Y	
p62140	rs838910	125287380	Intron 7		G/T	0.7286	95.2	0.3047	HapMap	Y	Y	
p62409	rs838911	125287111	Intron 7		C/T	0.5513	94.9	0.4211	HapMap	Y	Y	
p62615	rs7138386	125286905	Intron 7		T/C	0.4533	93.8	0.1137	HapMap	Y	Y	
p63483	rs838912	125286037	Intron 7		G/A	1	97.3	0.0867	HapMap	Y	Y	
p64772	rs5888	125284748	Exon 8	Ala350Ala	C/T	0.823	97.7	0.0961	SEQ	Y	Y	
p64923	rs838915	125284597	Intron 8		C/A	0.3967	96.8	0.1435	SEQ	Y	Y	
p65999	rs12819677	125283521	Intron 8		G/A	0.2879	95.4	0.2813	HapMap	Y	Y	
p67439	rs961170	125282081	Intron 8		G/A	0.0532	95.2	0.0893	HapMap	Y	Y	
p67700	rs1726374	125281820	Intron 8		G/A	0.2011	98.5	0.1933	HapMap	Y	Y	
p69013	rs7135117	125280507	Intron 8		A/G	0.0258	94.9	0.2901	HapMap	Y	Y	
p69699	rs10396210	125279821	Intron 8-splice site		G/A	1	94.9	0.1511	SEQ	Y	Y	
p69995delC	rs5801571	125279525	Intron 9		[C/-]	0.2836	96.1	0.2761	SEQ	Y	Y	
p70201		125279319	Intron 9		T/C	1	97.1	0.0010	SEQ	Y	Y-only in SKAT-O	Found in one subject with an outlier HDL-C value (above mean + 3.5 SD), then excluded from other analyses, except for SKAT-O test (see Figure 1).
p71867	rs7954022	125277653	Intron 9		C/T	0.8535	95.9	0.1323	HapMap	Y	Y	
p72197	rs838861	125277323	Intron 9		A/G	0.0045	95.4	0.3777	HapMap	Y	Y	
p72777	rs838862	125276743	Intron 9		C/T	0.7348	95.2	0.0887	HapMap	Y	Y	

SNP Name[a]	SNP ID[b]	Chr12 Position[c]	Location	Amino acid Change	Major/Minor Alleles	HWE *P*	%Call Rate	MAF	Source of Variants	Success in GT (n = 138)	Proceed to Analyses (n = 137)	Note
p75766	rs838866	125273754	Intron 9		T/C	0.4407	96.6	0.2116	HapMap	Y	Y	
p75778	rs7301120	125273742	Intron 9		C/T	1	93.9	0.1135	HapMap	Y	Y	
p76204	rs866793	125273316	Intron 9		A/G	1.66E-18	92.9	0.0520	HapMap	Y	N	Excluded due to a deviation from HWE
p76757	rs9919713	125272763	Intron 9		A/T	0.252	96.7	0.4390	HapMap	Y	Y	
p77181	rs146246031	125272339	Intron 9		T/C	1	96.2	0.0053	SEQ	Y	Y	
p77251	rs34339961	125272269	Intron 9		A/T	0.9341	95.4	0.1177	SEQ	Y	Y	
p77381	rs138499966	125272139	Intron 9		T/C	1	97	0.0046	SEQ	Y	Y	
p77620	rs377124254	125271900	Intron 10		G/A	1	96.2	0.0007	SEQ	Y	Y	
p77682	rs150082885	125271838	Intron 10		A/G	0.1549	95.4	0.0106	SEQ	Y	Y	
p77704		125271816	Intron 10		C/A	1	95.6	0.0040	SEQ	Y	Y	
p77842	rs2272310	125271678	Intron 10		G/A	1	98.2	0.0807	SEQ	Y	Y	
p78255	rs184052375	125271265	Intron 10		A/G	1	97.1	0.0072	SEQ	Y	Y	
p78402	rs838898	125271118	Intron 10		G/A	0.1137	89.7	0.0714	SEQ	Y	Y	
p78430	rs838897	125271090	Intron 10		C/G	0.0119	95.4	0.3830	SEQ	Y	Y	
p78747	rs2293440	125270773	Intron 11		T/C	0.5489	95.1	0.4112	SEQ	Y	Y	
p78791	rs75289200	125270729	Intron 11		T/C	0.9169	94.8	0.0321	SEQ	Y	Y	
p79721	rs838896	125269799	Intron 11		G/C	0.9259	97.7	0.3104	HapMap	Y	Y	
p79828	rs838895	125269692	Intron 11		C/G	0.8944	96.4	0.3171	Literature	Y	Y	
p80045	rs838893	125269475	Intron 11		G/A	0.9108	97.6	0.3244	HapMap	Y	Y	
p81863	rs185445624	125267657	Intron 11		G/A	1	97	0.0020	SEQ	Y	Y	
p82019	rs838890	125267501	Intron 11		C/T	0.343	95.1	0.0320	SEQ	Y	Y	
p82264	rs141545424	125267256	Exon 12	Gly501Gly	C/A	1	96.7	0.0007	SEQ	Y	Y	
p82340	rs77483223	125267180	Intron 12		G/A	1	95.9	0.0231	SEQ	Y	Y	
p82369	rs75446635	125267151	Intron 12		G/A	1	97	0.0059	SEQ	Y	Y	
p82434	rs838889	125267086	Intron 12		T/C	0.3344	96.6	0.0315	SEQ	Y	Y	
p83547	rs838887	125265973	Intron 12		C/G	0.5641	96.1	0.4564	HapMap	Y	Y	
p83884	rs701106	125265636	Intron 12		C/T	0.9346	98	0.2597	HapMap	Y	Y	
p86245	rs188375019	125263275	Intron 12		C/T	0.8126	96.7	0.0341	SEQ	Y	Y	
p86276	rs747155	125263244	Intron 12		C/T	0.8803	97.2	0.1495	SEQ	Y	Y	
p86316	rs701104	125263204	Intron 12		G/T	1	93.8	0.0487	SEQ	Y	Y	
p86481	rs701103	125263039	Exon 13-3' UTR	Gly499Arg (isoform2)	G/A	0.3376	96.6	0.2451	SEQ	Y	Y	
p86967	rs187492239	125262553	Exon 13-3' UTR		A/G	0.7536	96.4	0.0355	SEQ	Y	Y	
p87011	rs58032386	125262509	Exon 13-3' UTR		C/T	0.8106	97.6	0.1417	SEQ	Y	Y	
p87266	rs150512235	125262254	Exon 13-		T/C	1	99.4	0.0057	SEQ	Y	Y	

SNP Name[a]	SNP ID[b]	Chr12 Position[c]	Location	Amino acid Change	Major/Minor Alleles	HWE *P*	%Call Rate	MAF	Source of Variants	Success in GT (n = 138)	Proceed to Analyses (n = 137)	Note
			3' UTR[d]									
p87611	rs190688220	125261909	3' flanking		C/T	0.9286	96.3	0.0316	SEQ	Y	Y	
p87681	rs838883	125261839	3' flanking		G/A	1	93.9	0.0459	SEQ	Y	Y	
p87694		125261826	3' flanking		C/T	1	95.4	0.0020	SEQ	Y	Y	
p87723	rs838881	125261797	3' flanking		C/T	0.0678	97.1	0.3183	SEQ	Y	Y	
p87749	rs76465225	125261771	3' flanking		G/A	0.5802	97	0.0844	SEQ	Y	Y	
p87927	rs838880	125261593	3' flanking		G/A	0.5951	96.4	0.2414	SEQ	Y	Y	

del, deletion; GT, genotyping; HDL-C, high-density lipoprotein cholesterol; HWE, Hardy-Weinberg equilibrium; ins, insertion; MAF, minor allele frequency; Literature, variants selected from the literature; SD, standard deviation; SEQ, sequencing; SKAT-O, an optimal sequence kernel association test; SNP, single nucleotide polymorphism; UTR, untranslated region. N, no; Y, yes.

All alleles on the reverse strand. Splice site is defined as ± 20 bp from the start or end of an exon.

All 10 novel variants identified in this study have been submitted to dbSNP database (batch ID: SCARB1_AB): http://www.ncbi.nlm.nih.gov/SNP/snp_viewTable.cgi?handle=KAMBOH.

A total of 137 genotyped variants (68 variants identified by sequencing, 67 common HapMap-YRI tagSNPs, and 2 additional associated variants from the literatures) that passed genotyping quality controls were included in the downstream analyses.

An LD plot of 137 genotyped variants that passed genotyping quality controls and were included in the downstream analyses is shown in Additional file 11 Figure S4.

A list of 87 genotyped common tagSNPs (MAF ≥5%) identified by Tagger analysis using an r^2 cut-off of 0.90 is shown in Additional file 10 Table S7.

[a, c] RefSeq of *SCARB1*: hg19, NM_005505 (CHIP Bioinformatics).

[b] dbSNP build 139: GRCh37.p10.

[d] Close to a miRNA-145 seed site based on TargetScanHuman (version 6.2, http://www.targetscan.org/).
