# Supplementary material for: Genetic contribution of SCARB1 variants to lipid traits in African Blacks: a candidate gene association study
Source: BMC Med Genet. 2015 Nov 12;16:106. doi: 10.1186/s12881-015-0250-6 (PMC4643515; doi:10.1186/s12881-015-0250-6)
Supplement: Additional file 10: Table S7. — List of 87 SCARB1 genotyped common tagSNPs. (PDF 108 kb) [file 12881_2015_250_MOESM10_ESM.pdf]

Table S7. List of 87 *SCARB1* common tagSNPs identified by Tagger analysis (MAF ≥5% and r^2 ≥0.90) of the variants genotyped in the entire sample (capturing a total of 94 variants with a mean r^2 of 0.996).

Bin #	TagSNP	Alleles Captured
1	p1265	
2	p4072	
3	p5055	
4	p6600	
5	p10292	
6	p10991	
7	p13570	
8	p16565	
9	**p20207**	
10	p20694	
11	**p20741**	
12	p21145	
13	p22116	
14	p22168	
15	p22331	
16	p22675	
17	p28137	
18	p28692	
19	p28957	
20	p29749	
21	p31072	
22	p31938	
23	p32129	
24	p32273	
25	p32290	
26	p32395	
27	p32750	
28	p32777	
29	p32860	
30	p33531	
31	p36094	
32	p36361	
33	p36908	
34	p37095	
35	p41632	
36	p42467	
37	**p45516**	
38	p49537	
39	p49570delC	
40	**p49690**	
41	p50151	p48969
42	p51888	
43	p52096	
44	p52556	
45	p52610	

46	p52956	
47	p53359	
48	p53790	
49	p54492	
50	p55923	
51	**p55963**	
52	p57107	
53	p57508	p62615
54	p57592	
55	p58514	p56845
56	p58664	
57	p61872	
58	p62140	
59	p62409	
60	**p63483**	
61	**p64772**	
62	p64923	
63	p65999	p60255
64	p67439	
65	p67700	
66	p69013	p69995delC
67	p69699	
68	p71867	
69	p72197	
70	p72777	
71	p75766	
72	p75778	
73	p76757	
74	p77842	
75	p78402	
76	p78430	
77	p78747	
78	**p79721**	
79	**p79828**	p80045
80	p83547	
81	**p83884**	
82	p86276	
83	p86481	
84	p87011	
85	p87723	
86	p87749	
87	p87927	

del, deletion; MAF, minor allele frequency; SNP, single nucleotide polymorphism.
All SNPs are presented with “SNP Name”; see Additional file 9 Table S6 for details for each SNP.
SNPs with nominally significant evidence of single-site association with either high-density lipoprotein cholesterol or apolipoprotein A-I (P <0.05; Additional file 14 Table S9 and Additional file 15 Table S10) observed in the current study are shown in **bold**.
