# Supplementary material for: Genetic contribution of SCARB1 variants to lipid traits in African Blacks: a candidate gene association study
Source: BMC Med Genet. 2015 Nov 12;16:106. doi: 10.1186/s12881-015-0250-6 (PMC4643515; doi:10.1186/s12881-015-0250-6)
Supplement: Additional file 13: Table S8. — Covariates used for the statistical analysis of lipid variables. (PDF 78 kb) [file 12881_2015_250_MOESM13_ESM.pdf]

Table S8. Covariates used for the statistical analysis of lipid variables.

	Lipid Traits				
**Covariates**	**HDL-C**	**LDL-C**	**TG**	**ApoA-I**	**ApoB**
**Sex (M/F)**	X	X	X	X	
**Age, years**	X		X	X	
**Body mass index, kg/m^2**		X			X
**Waist, cm**	X		X		
**Current smoking (yes/no)**	X	X			
**Jobmin, min**	X	X	X		
**Staff (junior/senior)**		X			X

ApoA-I, apolipoprotein A-I; ApoB, apolipoprotein B; HDL-C, high-density lipoprotein cholesterol; LDL-C, low-density lipoprotein cholesterol; TG, triglycerides.
All phenotypic values were Box-Cox transformed prior to statistical analyses.
"X" represents covariates that were used in adjustment in statistical analyses.
Body mass index (BMI), weight (kg)/[height (m)]^2.
Jobmin, minutes of walking or biking to work each day.
Staff, occupational status: junior, non-professional staff with salary grades 1-6; and senior, professional and administrative staff with salary grades 7-16.
Waist, waist measurement at the narrowest point or at the umbilicus if the narrowest point was undetermined.
