# Supplementary material for: Genetic contribution of SCARB1 variants to lipid traits in African Blacks: a candidate gene association study
Source: BMC Med Genet. 2015 Nov 12;16:106. doi: 10.1186/s12881-015-0250-6 (PMC4643515; doi:10.1186/s12881-015-0250-6)
Supplement: Additional file 14: Table S9. — Single-site association results for 136 SCARB1 genotyped variants with HDL-C. (PDF 689 kb) [file 12881_2015_250_MOESM14_ESM.pdf]

Table S9. Single-site association results for 136 *SCARB1* genotyped variants with HDL-C.

SNP Name[a]	SNP ID[b]	Chr12 Position[c]	Location	Amino Acid Change	RegDB Score[d]	MA, MAF	Genotype	Genotype Count	Adjusted Mean (mg/dL)	SD (mg/dL)	β	SE	*P*	FDR[e]
**MAF ≥5%**														
p1265	rs2070242	125348255	Exon 1	Ser4Ser	2b	T, 0.1284	CC/CT/TT	541/163/10	48.38/46.08/49.75	12.44/13.11/12.75	-0.5040	0.2906	0.0833	0.4530
p4072	rs7139401	125345448	Intron 1		3b	C, 0.4386	TT/TC/CC	232/343/147	47.46/48.27/47.73	13.65/12.32/11.97	0.0877	0.1905	0.6453	0.8199
p5055	rs11057869	125344465	Intron 1		7	A, 0.0740	AA/GA/GG	7/96/638	45.72/48.5/47.75	12.97/12.78/12.69	0.0976	0.3538	0.7828	0.9022
p6600	rs12831105	125342920	Intron 1		7	T, 0.1188	CC/CT/TT	566/151/11	47.51/49.2/47.82	12.24/14.20/12.34	0.3570	0.2933	0.2239	0.6829
p10292	rs4765181	125339228	Intron 1		5	T, 0.2490	TT/GT/GG	44/285/417	46.42/48.72/47.39	12.15/12.86/12.52	0.1348	0.2201	0.5405	0.8199
p10991	rs10773112	125338529	Intron 1		4	A, 0.3534	GG/GA/AA	303/341/89	47.35/48.09/48.59	13.39/12.02/12.81	0.2146	0.2010	0.2860	0.6833
p13570	rs11057864	125335950	Intron 1		4	T, 0.1180	TT/GT/GG	14/147/581	49.3/47.24/47.89	9.25/11.91/12.98	-0.0393	0.2886	0.8917	0.9622
p16565	rs10773111	125332955	Intron 1		6	A, 0.1928	GG/GA/AA	481/239/24	47.65/48.23/47.55	12.70/12.62/13.49	0.1089	0.2436	0.6550	0.8199
p20207	rs11057853	125329313	Intron 1		5	A, 0.4484	AA/GA/GG	150/360/228	48.27/48.84/45.85	12.70/13.23/11.74	0.4082	0.1925	**0.0343**	0.4235
p20694	rs11057852	125328826	Intron 1		7	A, 0.1050	GG/GA/AA	581/130/11	48.04/47.5/49.33	12.91/11.75/9.83	-0.0308	0.3061	0.9198	0.9622
p20741	rs11057851	125328779	Intron 1		5	T, 0.3237	TT/CT/CC	72/334/328	43.9/47.68/48.86	11.05/13.00/12.57	-0.5924	0.2067	**0.0043**	**0.1465**
p21145	rs3924313	125328375	Intron 1		6	T, 0.1772	CC/CT/TT	503/219/22	47.6/48.13/48.4	12.50/13.00/9.98	0.1488	0.2489	0.5502	0.8199
p22116	rs12370382	125327404	Intron 1		1f	A, 0.0645	GG/GA/AA	643/86/3	47.57/49.06/35.39	12.67/13.28/6.62	0.1369	0.3965	0.7300	0.8709
p22168	rs7137797	125327352	Intron 1		2b	C, 0.3977	CC/TC/TT	113/355/259	48.48/48.04/47.1	13.14/12.49/12.77	0.2153	0.1976	0.2763	0.6833
p22331	rs6488944	125327189	Intron 1		4	G, 0.1474	TT/TG/GG	522/188/12	47.54/48.91/47.47	12.66/12.99/12.80	0.3091	0.2778	0.2661	0.6829
p22675	rs12425134	125326845	Intron 1		4	T, 0.0526	TT/GT/GG	2/75/662	54.65/49.92/47.6	4.88/11.66/12.79	0.7556	0.4221	0.0738	0.4530
p28137	rs12229555	125321383	Intron 1		7	G, 0.3896	GG/AG/AA	99/373/257	46.88/47.29/49.16	12.96/12.60/12.64	-0.3976	0.2025	0.0500	0.4473
p28692	rs4765622	125320828	Intron 1		5	T, 0.2565	TT/CT/CC	43/289/403	47.61/48.48/47.36	13.61/12.50/12.84	0.1962	0.2242	0.3817	0.7635
p28957	rs11057844	125320563	Intron 1		5	A, 0.2362	GG/GA/AA	428/263/40	47.34/47.87/50.96	13.11/12.40/10.13	0.3671	0.2278	0.1075	0.5151
p29749	rs10846751	125319771	Intron 1		7	T, 0.4492	TT/CT/CC	142/365/210	46.42/47.81/48.73	13.57/12.59/12.27	-0.3558	0.1961	0.0701	0.4530
p31072	rs10846749	125318448	Intron 1		4	G, 0.4461	CC/CG/GG	222/366/144	48.83/47.61/46.39	12.22/12.86/13.04	-0.3792	0.1940	0.0510	0.4473
p31938	rs10744182	125317582	Intron 1		5	A, 0.1837	AA/GA/GG	25/221/487	45.31/47.42/47.94	16.27/13.13/12.29	-0.2885	0.2474	0.2438	0.6829
p32129	rs10773107	125317391	Intron 1		7	T, 0.1009	TT/GT/GG	9/132/593	47/46.79/48.03	8.10/12.92/12.72	-0.3186	0.3141	0.3107	0.6927
p32273	rs12580803	125317247	Intron 1		5	C, 0.1006	TT/TC/CC	619/114/18	47.7/47.52/51.35	12.85/11.79/13.59	0.2010	0.2968	0.4984	0.8199
p32290	rs10744181	125317230	Intron 1		5	C, 0.1238	TT/TC/CC	547/141/18	47.89/48.46/42.63	12.60/13.86/9.02	-0.1852	0.2851	0.5162	0.8199
p32395	rs12581963	125317125	Intron 1		5	T, 0.1314	TT/CT/CC	11/179/557	47.47/46.69/48.1	8.81/11.70/12.94	-0.3173	0.2808	0.2589	0.6829

p32750	rs7967521	125316770	Intron 1		7	G, 0.3425	GG/AG/AA	89/306/310	45.26/48.42/47.89	11.20/12.62/13.26	-0.2137	0.2034	0.2936	0.6833
p32777	rs11057841	125316743	Intron 1		7	A, 0.2805	GG/GA/AA	367/307/50	47.82/48.49/44.86	12.82/12.79/10.19	-0.1217	0.2187	0.5781	0.8199
p32860	rs7967406	125316660	Intron 1		6	C, 0.0991	CC/AC/AA	9/131/608	46.8/48.71/47.54	8.12/12.43/12.81	0.2647	0.3141	0.3996	0.7876
p33531	rs11057838	125315989	Intron 1		7	A, 0.2278	CC/CA/AA	439/254/41	48.09/47.82/45.51	12.94/12.64/10.74	-0.2064	0.2258	0.3609	0.7635
p36094	rs11608336	125313426	Intron 1		4	A, 0.1543	GG/GA/AA	521/195/15	47.49/48.96/48.39	12.47/13.43/10.99	0.3354	0.2678	0.2108	0.6829
p36461	rs4765178	125313059	Intron 1		4	T, 0.1671	TT/CT/CC	19/201/496	47.45/47.5/48.12	10.94/12.52/12.99	-0.1369	0.2628	0.6024	0.8199
p36908	rs10846745	125312612	Intron 1		4	G, 0.3257	GG/CG/CC	76/334/329	44.67/49.15/47.23	10.95/13.05/12.30	-0.0384	0.2045	0.8511	0.9488
p37095	rs10846744	125312425	Intron 1		4	G, 0.3056	CC/CG/GG	348/340/57	47.72/47.75/48.9	13.10/11.99/13.09	0.1168	0.2125	0.5829	0.8199
p41632	rs6488943	125307888	Intron 1		5	C, 0.2954	CC/AC/AA	50/309/337	45.63/48.2/48.34	9.68/13.16/12.65	-0.2195	0.2226	0.3244	0.7041
p42467	rs11057830	125307053	Intron 1		7	T, 0.1523	TT/CT/CC	13/201/523	40.37/48.03/47.91	15.96/13.41/12.33	-0.2810	0.2718	0.3015	0.6833
p45516	rs1902569	125304004	Intron 1		5	A, 0.1544	AA/GA/GG	18/190/519	49.5/49.31/47.28	12.57/11.81/12.95	0.5447	0.2629	**0.0386**	0.4375
p48969	rs2343394	125300551	Intron 2		5	T, 0.1898	TT/CT/CC	32/225/491	47.56/48.89/47.23	11.15/12.64/12.73	0.3165	0.2352	0.1788	0.6236
p49537	rs7305310	125299983	Intron 2		5	T, 0.1007	CC/CT/TT	595/117/16	48.06/47.04/45.04	12.87/12.58/9.83	-0.3396	0.2991	0.2566	0.6829
p49570 delC	rs145376237	125299950	Intron 2		5	delC, 0.2276	DD/WD/WW	36/260/432	44.33/49.8/46.89	11.46/13.26/12.43	0.3121	0.2311	0.1773	0.6236
p49690	rs4765615	125299830	Intron 2		5	A, 0.4426	AA/GA/GG	156/318/244	45.87/48/49.03	13.19/12.44/13.01	-0.4646	0.1866	**0.0130**	0.2526
p50151	rs2278986	125299369	Intron 3		5	C, 0.1933	CC/TC/TT	33/225/484	49.64/48.72/47.21	11.26/12.41/12.86	0.4333	0.2350	0.0656	0.4530
p51888	rs7138304	125297632	Intron 4		2b	T, 0.1079	TT/CT/CC	16/129/589	45.08/48.13/47.83	9.22/12.36/12.75	-0.0610	0.2898	0.8334	0.9367
p52096	rs10846739	125297424	Intron 4		3a	G, 0.4693	GG/AG/AA	169/343/217	46.18/49.5/46.33	13.08/12.63/12.17	0.0195	0.1868	0.9168	0.9622
p52556	rs11057820	125296964	Intron 4		5	A, 0.1000	AA/GA/GG	12/124/610	40.36/48.71/47.69	11.34/13.80/12.49	-0.1513	0.3081	0.6235	0.8199
p52610	rs10846738	125296910	Intron 4		4	T, 0.1349	TT/CT/CC	12/172/545	46.62/48.16/47.93	12.58/13.45/12.36	-0.0102	0.2804	0.9710	0.9848
p52956	rs77740046	125296564	Intron 4		5	T, 0.0546	CC/CT/TT	651/75/3	47.69/48.97/46.21	12.89/11.66/13.34	0.3300	0.4168	0.4288	0.8025
p53359	rs112371713	125296161	Intron 5		5	A, 0.1243	AA/GA/GG	9/160/549	44.06/49.59/47.4	13.40/12.95/12.69	0.4193	0.3018	0.1651	0.6236
p53790	rs4765614	125295730	Intron 5		5	A, 0.2653	GG/GA/AA	399/276/58	48.17/48.05/44.21	13.17/12.42/10.42	-0.3281	0.2118	0.1218	0.5370
p54492	rs61762481	125295028	Intron 5		4	A, 0.1005	AA/GA/GG	8/137/607	47.58/47.31/47.92	8.15/12.76/12.74	-0.1522	0.3119	0.6257	0.8199
p55923	rs838900	125293597	Intron 6		7	A, 0.3921	AA/GA/GG	113/345/275	47.05/49.45/46.18	13.85/13.31/11.20	0.2787	0.1957	0.1549	0.6194
p55963	rs7134858	125293557	Intron 6		6	T, 0.1560	TT/CT/CC	24/184/532	50.7/48.34/47.23	7.08/11.91/12.90	0.4418	0.2520	0.0799	0.4530
p56845	rs838902	125292675	Intron 6		5	G, 0.4249	AA/AG/GG	249/347/141	48.01/47.88/47.42	12.84/12.60/12.79	-0.0786	0.1905	0.6801	0.8343
p57107	rs5892	125292413	Exon 7	Phe301Phe	4	T, 0.0589	CC/CT/TT	656/85/1	47.84/47.53/54.22	12.92/11.23/NA	0.0079	0.4138	0.9848	0.9848
p57508	rs71458866	125292012	Intron 7		4	A, 0.1130	AA/GA/GG	13/144/594	47.5/47.11/47.93	9.18/12.92/12.68	-0.1926	0.2914	0.5089	0.8199
p57592	rs838903	125291928	Intron 7		4	A, 0.3763	AA/GA/GG	111/334/290	47.18/47.64/48.29	11.62/13.06/12.86	-0.1598	0.1959	0.4149	0.8025
p58514	rs838905	125291006	Intron 7		4	C, 0.4329	CC/TC/TT	146/350/243	47.34/47.97/48.02	12.68/12.56/13.04	-0.0855	0.1905	0.6536	0.8199

p58664	rs865716	125290856	Intron 7		5	T, 0.2708	AA/AT/TT	395/268/62	48.35/46.87/49.05	13.05/12.47/10.76	-0.0965	0.2094	0.6449	0.8199
p60255	rs3782287	125289265	Intron 7		5	T, 0.2831	CC/CT/TT	378/312/51	46.89/48.96/46.64	12.02/13.23/14.24	0.2555	0.2197	0.2453	0.6829
p61872	rs838909	125287648	Intron 7		4	T, 0.2199	CC/CT/TT	449/249/36	47.59/48.12/48.15	12.60/13.16/10.94	0.1223	0.2297	0.5945	0.8199
p62140	rs838910	125287380	Intron 7		5	T, 0.3047	GG/GT/TT	355/304/70	47.6/48.51/45.61	12.61/12.92/12.07	-0.0759	0.2071	0.7143	0.8597
p62409	rs838911	125287111	Intron 7		5	T, 0.4211	CC/CT/TT	247/347/133	48.21/48.12/46.87	13.10/12.87/11.68	-0.1556	0.1934	0.4213	0.8025
p62615	rs7138386	125286905	Intron 7		5	C, 0.1137	TT/TC/CC	568/138/12	48.18/46.68/50.88	12.66/12.76/9.42	-0.2083	0.2982	0.4851	0.8199
p63483	rs838912	125286037	Intron 7		7	A, 0.0867	AA/GA/GG	6/117/615	47.88/48.51/47.79	8.05/12.85/12.52	0.1814	0.3311	0.5840	0.8199
p64772	rs5888	125284748	Exon 8	Ala350Ala	3a	T, 0.0961	CC/CT/TT	605/129/8	47.6/48.54/52.11	12.65/13.16/10.27	0.3620	0.3186	0.2561	0.6829
p64923	rs838915	125284597	Intron 8		5	A, 0.1435	AA/CA/CC	19/177/539	45.25/48.03/47.87	9.15/12.97/12.72	-0.0858	0.2654	0.7466	0.8829
p65999	rs12819677	125283521	Intron 8		6	A, 0.2813	GG/GA/AA	371/310/50	46.78/49.09/47.82	12.14/13.01/13.90	0.4021	0.2192	0.0670	0.4530
p67439	rs961170	125282081	Intron 8		4	A, 0.0893	AA/GA/GG	11/108/610	45.83/48.01/47.82	17.60/12.30/12.70	-0.0481	0.3220	0.8812	0.9622
p67700	rs1726374	125281820	Intron 8		7	A, 0.1933	GG/GA/AA	491/221/35	47.61/47.44/51.33	12.81/12.01/14.89	0.2289	0.2330	0.3262	0.7041
p69013	rs7135117	125280507	Intron 8		7	G, 0.2901	GG/AG/AA	75/275/377	48.2/48.1/47.63	12.45/12.42/13.01	0.1133	0.2024	0.5758	0.8199
p69699	rs10396210	125279821	Intron 8-splice site		4	A, 0.1511	AA/GA/GG	17/186/523	46.35/46.27/48.29	10.51/11.82/13.06	-0.4778	0.2675	0.0745	0.4530
p69995 delC	rs5801571	125279525	Intron 9		5	delC, 0.2761	DD/WD/WW	63/281/386	47.7/47.72/47.45	13.42/12.34/13.04	0.0614	0.2123	0.7724	0.8978
p71867	rs7954022	125277653	Intron 9		5	T, 0.1323	TT/CT/CC	11/172/552	45/49.47/47.43	12.10/12.93/12.59	0.3876	0.2864	0.1764	0.6236
p72197	rs838861	125277323	Intron 9		7	G, 0.3777	AA/AG/GG	300/308/123	48.56/47.21/47.97	13.20/12.13/13.06	-0.1415	0.1883	0.4527	0.8101
p72777	rs838862	125276743	Intron 9		5	T, 0.0887	CC/CT/TT	607/115/7	47.9/47.97/51.52	12.46/13.53/18.41	0.1013	0.3334	0.7613	0.8925
p75766	rs838866	125273754	Intron 9		6	C, 0.2116	TT/TC/CC	457/239/38	48.02/47.68/47.04	12.90/12.55/10.89	-0.1058	0.2276	0.6423	0.8199
p75778	rs7301120	125273742	Intron 9		6	T, 0.1135	TT/CT/CC	9/147/563	50.67/46.79/48.17	7.48/12.53/12.82	-0.2367	0.3041	0.4366	0.8025
p76757	rs9919713	125272763	Intron 9		6	T, 0.4390	AA/AT/TT	235/347/151	48.16/47.8/47.14	13.78/12.06/12.19	-0.1264	0.1892	0.5044	0.8199
p77251	rs34339961	125272269	Intron 9		6	T, 0.1177	AA/AT/TT	561/151/11	47.91/46.76/50.68	12.87/12.10/8.13	-0.1372	0.2955	0.6426	0.8199
p77842	rs2272310	125271678	Intron 10		5	A, 0.0807	AA/GA/GG	5/110/630	51.11/48.16/47.65	12.21/13.23/12.64	0.2011	0.3494	0.5651	0.8199
p78402	rs838898	125271118	Intron 10		5	A, 0.0714	AA/GA/GG	7/86/594	45.47/48/48.01	7.94/12.21/13.05	-0.0602	0.3738	0.8720	0.9622
p78430	rs838897	125271090	Intron 10		5	G, 0.3830	GG/CG/CC	125/308/291	46.86/48.36/47.33	12.37/12.40/12.86	0.0070	0.1877	0.9704	0.9848
p78747	rs2293440	125270773	Intron 11		5	C, 0.4112	CC/TC/TT	128/342/252	46.7/47.72/48.12	10.50/12.59/13.48	-0.1684	0.1919	0.3806	0.7635
p79721	rs838896	125269799	Intron 11		5	C, 0.3104	GG/GC/CC	349/319/73	47.39/47.6/50.86	13.07/12.12/12.38	0.3565	0.2045	0.0817	0.4530
p79828	rs838895	125269692	Intron 11		5	G, 0.3171	GG/CG/CC	74/322/337	50.65/48.22/46.95	12.74/12.27/13.10	0.4961	0.2059	**0.0162**	0.2756
p80045	rs838893	125269475	Intron 11		5	A, 0.3244	GG/GA/AA	335/325/81	47.17/48.1/49.33	12.98/12.45/12.54	0.3127	0.2022	0.1224	0.5370
p83547	rs838887	125265973	Intron 12		5	G, 0.4564	CC/CG/GG	225/357/154	46.99/48.49/47.62	12.57/12.51/13.34	0.1202	0.1898	0.5267	0.8199
p83884	rs701106	125265636	Intron 12		5	T, 0.2597	TT/CT/CC	49/289/405	50.45/47.79/47.56	13.58/12.76/12.53	0.2471	0.2192	0.2601	0.6829

p86276	rs747155	125263244	Intron 12		2b	T, 0.1495	TT/CT/CC	17/187/533	48.37/48.5/47.47	13.90/11.86/12.96	0.2793	0.2681	0.2980	0.6833
p86481	rs701103	125263039	Exon 13-3' UTR	Gly499Arg (isoform 2)	5	A, 0.2451	AA/GA/GG	50/259/424	46.92/48.83/47.38	13.72/12.39/12.59	0.1642	0.2169	0.4492	0.8101
p87011	rs58032386	125262509	Exon 13-3' UTR		2a	T, 0.1417	CC/CT/TT	544/183/14	47.8/48.03/47.93	12.88/11.70/16.17	0.0575	0.2733	0.8333	0.9367
p87723	rs838881	125261797	3' flanking		6	T, 0.3183	TT/CT/CC	63/341/332	47.01/48.22/47.16	12.74/12.21/13.05	0.1390	0.2132	0.5146	0.8199
p87749	rs76465225	125261771	3' flanking		7	A, 0.0844	AA/GA/GG	7/109/620	43.03/47.81/47.93	19.91/12.52/12.66	-0.1992	0.3402	0.5583	0.8199
p87927	rs838880	125261593	3' flanking		5	A, 0.2414	AA/GA/GG	39/275/418	44.74/48.67/47.52	12.40/12.15/13.23	0.0198	0.2302	0.9314	0.9670
**MAF between 1-5%**														
p1316	rs10396208	125348204	Exon 1	Cys21Cys	2b	T, 0.0476	CC/CT/TT	652/58/4	47.77/49.2/47.27	12.64/12.69/18.13	0.2987	0.4428	0.5001	0.8199
p1419	rs201717369	125348101	Intron 1		4	A, 0.0121	AA/GA/GG	1/14/707	55.72/48.94/47.8	NA/15.28/12.67	0.4892	0.8717	0.5748	0.8199
p7650	rs11615630	125341870	Intron 1		5	A, 0.0436	GG/GA	685/67	47.63/49.16	12.20/16.83	0.3382	0.4681	0.4702	0.8199
p45627	rs12297372	125303893	Intron 1		5	G, 0.0487	GG/AG/AA	1/68/659	30.17/48.3/47.89	NA/12.68/12.74	-0.0483	0.4556	0.9156	0.9622
p46964	rs114061302	125302556	Intron 1		4	A, 0.0388	AA/GA/GG	1/55/688	34.33/48.54/47.73	NA/13.75/12.65	0.0526	0.4975	0.9158	0.9622
p50118	rs58710319	125299402	Intron 3		5	T, 0.0208	CT/CC	31/711	50.42/47.73	13.43/12.65	0.7885	0.6704	0.2399	0.6829
p50380	rs141748317	125299140	Intron 3		2b	G, 0.0112	AA/AG	723/15	47.81/48.13	12.68/15.76	-0.0273	0.9564	0.9772	0.9848
p50489	rs61320152	125299031	Intron 3		4	T, 0.0257	GG/GT	699/38	47.82/48.65	12.69/13.60	0.2390	0.6110	0.6958	0.8449
p54445	rs60910935	125295075	Intron 5		4	G, 0.0418	AA/AG/GG	650/56/2	47.98/46.86/53.78	13.00/10.86/1.84	-0.1247	0.4830	0.7963	0.9101
p54475	rs60227139	125295045	Intron 5		4	T, 0.0437	CC/CT/TT	670/61/2	47.84/47.01/53.7	12.92/10.81/1.84	-0.0602	0.4628	0.8966	0.9622
p77682	rs150082885	125271838	Intron 10		5	G, 0.0106	AA/AG/GG	716/13/1	47.88/48.15/31.47	12.76/13.91/NA	-0.5377	0.9010	0.5509	0.8199
p78791	rs75289200	125270729	Intron 11		5	C, 0.0321	TC/TT	46/679	50.26/47.65	14.67/12.57	0.7037	0.5581	0.2078	0.6829
p82019	rs838890	125267501	Intron 11		5	T, 0.0320	CC/CT/TT	683/42/2	48.07/44.74/39.26	12.91/10.83/17.25	-1.0051	0.5373	0.0618	0.4530
p82340	rs77483223	125267180	Intron 12		5	A, 0.0231	GA/GG	35/699	44.56/47.96	11.90/12.76	-1.0458	0.6373	0.1012	0.5098
p82434	rs838889	125267086	Intron 12		5	C, 0.0315	CC/TC/TT	2/42/695	39.33/44.57/48.04	17.25/10.84/12.85	-1.0389	0.5352	0.0526	0.4473
p86245	rs188375019	125263275	Intron 12		4	T, 0.0341	CC/CT	690/50	47.7/50.2	12.65/12.86	0.7447	0.5344	0.1639	0.6236
p86316	rs701104	125263204	Intron 12		4	T, 0.0487	TT/GT/GG	2/66/643	39.94/45.01/48.22	7.78/11.60/12.86	-0.9838	0.4484	**0.0286**	0.3887
p86967	rs187492239	125262553	Exon 13-3' UTR		4	G, 0.0355	AA/AG	686/52	47.68/50.25	12.71/12.51	0.7743	0.5256	0.1412	0.5818
p87611	rs190688220	125261909	3' flanking		4	T, 0.0316	CC/CT	691/46	47.66/50.44	12.72/12.61	0.8329	0.5573	0.1355	0.5759
p87681	rs838883	125261839	3' flanking		5	A, 0.0459	AA/GA/GG	1/65/646	45.6/44.85/48.19	NA/11.43/12.90	-0.9433	0.4647	**0.0427**	0.4471
**MAF ≤1%**														
p1048 insC (1048_ 1049)		125348472	Exon 1-5' UTR		2a	insC, 0.0079	WI/WW	12/723	45.31/47.92	15.77/12.65	-0.9389	1.0655	0.3785	0.7635
p49759	rs146272788	125299761	Intron 2		5	T, 0.0020	CC/CT	725/3	47.84/56.6	12.78/13.38	2.5988	2.1256	0.2219	0.6829

p49978	rs5891	125299542	Exon 3	Val135Ile	5	A, 0.0058	GA/GG	9/743	52.42/47.7	18.19/12.61	1.3374	1.2347	0.2791	0.6833
p50024	rs368880622	125299496	Intron 3		5	T, 0.0026	GG/GT	737/3	47.83/53.32	12.72/12.83	1.6506	2.1188	0.4362	0.8025
p50954		125298566	Intron 4		5	C, 0.0007	TC/TT	1/735	63.83/47.81	NA/12.71	4.5639	3.6627	0.2131	0.6829
p52919		125296601	Intron 4		5	T, 0.0013	GG/GT	734/2	47.87/24.62	12.71/9.26	-7.4063	2.5863	**0.0043**	0.1465
p52995	rs113910315	125296525	Intron 4-splice site		5	G, 0.0020	TG/TT	3/740	43.06/47.83	12.96/12.72	-1.4175	2.1192	0.5038	0.8199
p53372	rs115604379	125296148	Intron 5		5	T, 0.0066	CC/CT	729/10	47.68/58.2	12.64/13.03	3.0372	1.1642	**0.0093**	0.2190
p54611		125294909	Intron 5		4	C, 0.0007	TC/TT	1/742	19.59/47.86	NA/12.68	-9.5243	3.6710	**0.0097**	0.2190
p54627		125294893	Intron 5		4	C, 0.0020	GC/GG	3/733	50.9/47.79	15.45/12.75	0.9473	2.1330	0.6571	0.8199
p54856		125294664	Intron 6		4	T, 0.0007	CC/CT	742/1	47.85/21.48	12.70/NA	-8.4305	3.6579	**0.0215**	0.3243
p57004	rs187562853	125292516	Intron 6		4	A, 0.0098	GG/GA	721/15	47.61/53.57	12.63/18.00	1.6474	0.9619	0.0872	0.4560
p77181	rs146246031	125272339	Intron 9		7	C, 0.0053	TC/TT	6/731	45.71/47.91	12.57/12.72	-0.6173	1.5007	0.6809	0.8343
p77381	rs138499966	125272139	Intron 9		6	C, 0.0046	TC/TT	7/735	53.23/47.76	17.65/12.68	1.5523	1.3921	0.2652	0.6829
p77620	rs377124254	125271900	Intron 10		5	A, 0.0007	GA/GG	1/735	90.2/47.77	NA/12.67	11.5518	3.6514	**0.0016**	0.1104
p77704		125271816	Intron 10		5	A, 0.0040	CA/CC	5/726	42.75/47.86	9.16/12.79	-1.4602	1.6591	0.3791	0.7635
p78255	rs184052375	125271265	Intron 10		4	G, 0.0072	AA/AG	732/11	47.75/52.05	12.64/17.17	1.1808	1.1134	0.2893	0.6833
p81863	rs185445624	125267657	Intron 11		5	A, 0.0020	GA/GG	3/739	45.05/47.83	20.36/12.71	-0.9612	2.1241	0.6510	0.8199
p82264	rs141545424	125267256	Exon 12	Gly501Gly	5	A, 0.0007	CA/CC	1/739	90.31/47.77	NA/12.66	11.5850	3.6469	**0.0016**	0.1104
p82369	rs75446635	125267151	Intron 12		5	A, 0.0059	GA/GG	9/733	50.65/47.8	20.95/12.59	0.5896	1.2312	0.6322	0.8199
p87266	rs150512235	125262254	Exon 13-3' UTR[f]		4	C, 0.0057	TC/TT	9/746	48.46/47.75	20.83/12.58	-0.0325	1.2316	0.9789	0.9848
p87694		125261826	3' flanking		5	T, 0.0020	CC/CT	722/3	47.85/59.52	12.75/12.25	3.4021	2.1251	0.1098	0.5151

del/D, deletion; FDR, false discovery rate; HDL-C, high-density lipoprotein cholesterol; ins/I, insertion; MA, minor allele; MAF, minor allele frequency; NA, not analyzed; RegDB, RegulomeDB score; SD, standard deviation; SE, standard error; SNP, single nucleotide polymorphism; UTR, untranslated region; W, wild type allele for insertion or deletion on RefSeq.

All alleles on the reverse strand. Splice site is defined as ± 20 bp from the start or end of an exon.

All 10 novel variants identified in this study have been submitted to dbSNP database (batch ID: SCARB1_AB): http://www.ncbi.nlm.nih.gov/SNP/snp_viewTable.cgi?handle=KAMBOH.

HDL-C values were Box-Cox transformed. Results were adjusted for covariates: sex, age, waist, current smoking (yes/no), and minutes of daily walking or biking to work (jobmin).

Nominally significant *P*-values (*P* <0.05) and FDR values that passed the threshold (FDR <0.20) are shown in **bold**.

[a, c] RefSeq of *SCARB1*: hg19, NM_005505 (CHIP Bioinformatics).

[b] dbSNP build 139: GRCh37.p10.

[d] The RegulomeDB (version 1.0) scoring scheme is described at the footnote of Additional file 4 Table S4 or can be seen at http://regulome.stanford.edu/help.

[e] Corresponding to a *q*-value from Benjamini-Hochberg procedure.

[f] Close to a miRNA-145 seed site based on TargetScanHuman (version 6.2, http://www.targetscan.org/).
