# Supplementary material for: Genetic contribution of SCARB1 variants to lipid traits in African Blacks: a candidate gene association study
Source: BMC Med Genet. 2015 Nov 12;16:106. doi: 10.1186/s12881-015-0250-6 (PMC4643515; doi:10.1186/s12881-015-0250-6)
Supplement: Additional file 15: Table S10. — Single-site association results for 136 SCARB1 genotyped variants with ApoA-I. (PDF 686 kb) [file 12881_2015_250_MOESM15_ESM.pdf]

Table S10. Single-site association results for 136 *SCARB1* genotyped variants with ApoA-I.

SNP Name[a]	SNP ID[b]	Chr12 Position[c]	Location	Amino Acid Change	RegDB Score[d]	MA, MAF	Genotype	Genotype Count	Adjusted Mean (mg/dL)	SD (mg/dL)	β	SE	*P*	FDR[e]
**MAF ≥5%**														
p1265	rs2070242	125348255	Exon 1	Ser4Ser	2b	T, 0.1284	CC/CT/TT	545/165/10	138.25/132.98/141.25	28.10/26.38/36.12	-1.2535	0.7296	0.0862	0.5583
p4072	rs7139401	125345448	Intron 1		3b	C, 0.4386	TT/TC/CC	236/350/148	136.26/138.43/135.18	27.17/28.38/27.51	-0.0802	0.4732	0.8654	0.9654
p5055	rs11057869	125344465	Intron 1		7	A, 0.0740	AA/GA/GG	7/95/650	138.23/138.44/136.59	27.94/25.35/28.24	0.5638	0.8858	0.5247	0.8490
p6600	rs12831105	125342920	Intron 1		7	T, 0.1188	CC/CT/TT	571/152/10	137.08/135.15/135.11	27.64/27.93/25.69	-0.5681	0.7373	0.4412	0.8109
p10292	rs4765181	125339228	Intron 1		5	T, 0.2490	TT/GT/GG	44/287/426	132.59/138.1/136.53	27.04/27.35/28.00	-0.0286	0.5474	0.9583	0.9931
p10991	rs10773112	125338529	Intron 1		4	A, 0.3534	GG/GA/AA	309/339/90	135/138.69/135.87	28.55/26.69/27.19	0.4896	0.4950	0.3230	0.7761
p13570	rs11057864	125335950	Intron 1		4	T, 0.1180	TT/GT/GG	15/147/591	131.15/138.99/136.48	19.01/28.45/27.80	0.3006	0.7107	0.6725	0.9051
p16565	rs10773111	125332955	Intron 1		6	A, 0.1928	GG/GA/AA	486/246/23	135.99/138.39/134.71	27.30/28.56/28.10	0.4484	0.6077	0.4608	0.8230
p20207	rs11057853	125329313	Intron 1		5	A, 0.4484	AA/GA/GG	153/366/230	136.24/139.87/132.18	27.54/29.21/25.20	0.8422	0.4726	0.0751	0.5540
p20694	rs11057852	125328826	Intron 1		7	A, 0.1050	GG/GA/AA	587/129/11	136.59/138.27/135.18	27.73/28.21/20.66	0.3519	0.7640	0.6452	0.9003
p20741	rs11057851	125328779	Intron 1		5	T, 0.3237	TT/CT/CC	73/336/337	130.12/136.32/138.94	27.67/27.25/28.14	-1.2331	0.5117	**0.0162**	0.3186
p21145	rs3924313	125328375	Intron 1		6	T, 0.1772	CC/CT/TT	511/221/23	136.58/137.05/136.75	27.68/28.31/22.08	0.1215	0.6158	0.8437	0.9642
p22116	rs12370382	125327404	Intron 1		1f	A, 0.0645	GG/GA/AA	650/89/3	135.9/139.94/107.7	27.29/31.23/14.64	0.5264	0.9717	0.5882	0.8589
p22168	rs7137797	125327352	Intron 1		2b	C, 0.3977	CC/TC/TT	112/358/262	139.7/135.97/135.95	27.57/26.56/28.95	0.5066	0.4888	0.3003	0.7761
p22331	rs6488944	125327189	Intron 1		4	G, 0.1474	TT/TG/GG	524/191/12	136.47/138.31/134.29	27.51/28.77/27.05	0.3684	0.6902	0.5936	0.8589
p22675	rs12425134	125326845	Intron 1		4	T, 0.0526	TT/GT/GG	2/74/668	149.31/139.33/136.52	0.28/23.43/28.01	1.1151	1.0532	0.2900	0.7761
p28137	rs12229555	125321383	Intron 1		7	G, 0.3896	GG/AG/AA	100/373/265	134.51/136.62/138.27	24.37/27.67/28.92	-0.5752	0.5022	0.2524	0.7761
p28692	rs4765622	125320828	Intron 1		5	T, 0.2565	TT/CT/CC	42/298/406	134.55/140.06/135.18	28.51/29.32/26.22	0.7871	0.5534	0.1553	0.6416
p28957	rs11057844	125320563	Intron 1		5	A, 0.2362	GG/GA/AA	433/269/40	136.15/136.91/142.06	28.60/26.35/29.06	0.6107	0.5626	0.2781	0.7761
p29749	rs10846751	125319771	Intron 1		7	T, 0.4492	TT/CT/CC	142/366/215	132.92/137.38/138.1	25.65/27.79/28.67	-0.7763	0.4856	0.1104	0.5748
p31072	rs10846749	125318448	Intron 1		4	G, 0.4461	CC/CG/GG	226/371/146	139.23/136.07/134.5	28.54/28.23/26.18	-0.8005	0.4818	0.0970	0.5748
p31938	rs10744182	125317582	Intron 1		5	A, 0.1837	AA/GA/GG	25/226/493	131.55/136.6/137.11	27.78/27.47/28.13	-0.4389	0.6139	0.4748	0.8255
p32129	rs10773107	125317391	Intron 1		7	T, 0.1009	TT/GT/GG	9/131/605	151.68/137.5/136.38	25.54/28.08/27.45	0.8714	0.7738	0.2605	0.7761
p32273	rs12580803	125317247	Intron 1		5	C, 0.1006	TT/TC/CC	627/117/18	135.83/140.74/140.03	27.84/27.87/24.49	1.2550	0.7258	0.0842	0.5583
p32290	rs10744181	125317230	Intron 1		5	C, 0.1238	TT/TC/CC	551/143/18	136.61/139.47/129.44	27.77/28.30/23.29	0.1584	0.7002	0.8211	0.9545
p32395	rs12581963	125317125	Intron 1		5	T, 0.1314	TT/CT/CC	11/177/570	134.74/134.94/137.17	27.03/27.17/27.89	-0.6483	0.7013	0.3556	0.7761

p32750	rs7967521	125316770	Intron 1		7	G, 0.3425	GG/AG/AA	91/304/315	135.77/138.19/136.64	27.06/27.89/28.28	0.0564	0.5011	0.9104	0.9749
p32777	rs11057841	125316743	Intron 1		7	A, 0.2805	GG/GA/AA	374/303/52	136.83/137.07/136.83	27.62/27.46/29.55	0.0373	0.5407	0.9450	0.9923
p32860	rs7967406	125316660	Intron 1		6	C, 0.0991	CC/AC/AA	9/135/616	142.53/137.42/136.54	29.57/26.48/28.02	0.4768	0.7717	0.5369	0.8490
p33531	rs11057838	125315989	Intron 1		7	A, 0.2278	CC/CA/AA	440/259/40	136.66/137.21/137.74	28.28/26.63/27.18	0.1993	0.5594	0.7218	0.9136
p36094	rs11608336	125313426	Intron 1		4	A, 0.1543	GG/GA/AA	526/195/16	136.58/137.43/130.93	26.64/30.54/19.66	-0.0956	0.6599	0.8848	0.9654
p36461	rs4765178	125313059	Intron 1		4	T, 0.1671	TT/CT/CC	18/205/497	143.57/137.38/136.71	28.60/27.50/28.03	0.5087	0.6538	0.4368	0.8109
p36908	rs10846745	125312612	Intron 1		4	G, 0.3257	GG/CG/CC	78/335/337	133.81/138.5/136.23	26.52/28.07/27.17	0.0089	0.5014	0.9859	0.9965
p37095	rs10846744	125312425	Intron 1		4	G, 0.3056	CC/CG/GG	350/350/56	137.16/135.63/141.68	27.54/27.45/30.05	0.1538	0.5335	0.7732	0.9144
p41632	rs6488943	125307888	Intron 1		5	C, 0.2954	CC/AC/AA	52/312/340	139.91/137.51/137.18	25.93/27.34/27.81	0.3025	0.5440	0.5783	0.8571
p42467	rs11057830	125307053	Intron 1		7	T, 0.1523	TT/CT/CC	14/200/533	137.96/133.93/137.64	41.61/27.21/27.42	-0.9351	0.6668	0.1612	0.6416
p45516	rs1902569	125304004	Intron 1		5	A, 0.1544	AA/GA/GG	18/191/523	142.37/137.21/136.5	23.01/25.54/28.50	0.5127	0.6537	0.4331	0.8109
p48969	rs2343394	125300551	Intron 2		5	T, 0.1898	TT/CT/CC	32/226/501	138.49/138.05/135.89	20.81/26.09/28.67	0.6411	0.5825	0.2714	0.7761
p49537	rs7305310	125299983	Intron 2		5	T, 0.1007	CC/CT/TT	601/118/16	136.94/137.31/131.4	28.45/24.39/24.26	-0.2265	0.7408	0.7599	0.9144
p49570 delC	rs145376237	125299950	Intron 2		5	delC, 0.2276	DD/WD/WW	37/260/437	132.41/139.59/135.62	31.14/26.82/28.01	0.4929	0.5706	0.3880	0.7761
p49690	rs4765615	125299830	Intron 2		5	A, 0.4426	AA/GA/GG	157/323/244	132.82/137.57/138.51	31.67/26.38/26.87	-0.9139	0.4614	**0.0480**	0.5022
p50151	rs2278986	125299369	Intron 3		5	C, 0.1933	CC/TC/TT	33/226/494	142.72/137.51/135.83	21.16/25.91/28.90	0.8568	0.5828	0.1419	0.6311
p51888	rs7138304	125297632	Intron 4		2b	T, 0.1079	TT/CT/CC	16/130/593	130.58/138.07/136.64	24.75/23.20/28.33	-0.0031	0.7159	0.9965	0.9965
p52096	rs10846739	125297424	Intron 4		3a	G, 0.4693	GG/AG/AA	172/342/221	135.77/137.19/136.77	28.27/27.28/27.46	-0.1537	0.4586	0.7377	0.9144
p52556	rs11057820	125296964	Intron 4		5	A, 0.1000	AA/GA/GG	12/126/619	127.43/134.64/137.37	29.15/29.91/27.33	-1.1588	0.7609	0.1282	0.6225
p52610	rs10846738	125296910	Intron 4		4	T, 0.1349	TT/CT/CC	13/172/553	130.76/135.7/137.19	33.53/27.12/27.43	-0.6490	0.6886	0.3462	0.7761
p52956	rs77740046	125296564	Intron 4		5	T, 0.0546	CC/CT/TT	657/75/3	136.98/135.38/134.32	28.41/22.14/29.35	-0.4428	1.0350	0.6689	0.9051
p53359	rs112371713	125296161	Intron 5		5	A, 0.1243	AA/GA/GG	10/161/554	129.69/140.18/136.1	21.21/27.65/28.05	0.8266	0.7340	0.2605	0.7761
p53790	rs4765614	125295730	Intron 5		5	A, 0.2653	GG/GA/AA	402/277/59	137.25/136.67/133.06	28.76/25.80/28.38	-0.4621	0.5232	0.3775	0.7761
p54492	rs61762481	125295028	Intron 5		4	A, 0.1005	AA/GA/GG	8/137/618	148.92/134.86/137.03	32.80/27.06/27.71	-0.1546	0.7735	0.8416	0.9642
p55923	rs838900	125293597	Intron 6		7	A, 0.3921	AA/GA/GG	115/346/282	138.57/136.64/135.92	31.53/27.67/26.43	0.3606	0.4823	0.4549	0.8230
p55963	rs7134858	125293557	Intron 6		6	T, 0.1560	TT/CT/CC	24/190/537	147.89/139.6/135.05	30.36/25.98/28.12	1.7537	0.6260	**0.0052**	0.2918
p56845	rs838902	125292675	Intron 6		5	G, 0.4249	AA/AG/GG	256/350/143	136.96/138.11/134.33	28.98/27.23/26.41	-0.3052	0.4662	0.5129	0.8404
p57107	rs5892	125292413	Exon 7	Phe301Phe	4	T, 0.0589	CC/CT/TT	663/84/1	136.93/135.46/135.43	27.81/27.27/NA	-0.4697	1.0308	0.6488	0.9003
p57508	rs71458866	125292012	Intron 7		4	A, 0.1130	AA/GA/GG	12/147/602	136.02/134.65/137.08	32.39/27.43/27.74	-0.6325	0.7294	0.3861	0.7761
p57592	rs838903	125291928	Intron 7		4	A, 0.3763	AA/GA/GG	113/336/297	132.17/137.53/138	26.07/27.56/28.72	-0.7661	0.4801	0.1109	0.5748
p58514	rs838905	125291006	Intron 7		4	C, 0.4329	CC/TC/TT	149/352/250	134.23/138.05/137.42	26.43/27.51/28.81	-0.4213	0.4644	0.3646	0.7761

p58664	rs865716	125290856	Intron 7		5	T, 0.2708	AA/AT/TT	395/272/63	136.68/135.18/144.1	28.06/26.58/28.78	0.5369	0.5185	0.3008	0.7761
p60255	rs3782287	125289265	Intron 7		5	T, 0.2831	CC/CT/TT	384/314/55	136.2/136.74/139.92	26.41/28.49/33.12	0.3715	0.5324	0.4856	0.8255
p61872	rs838909	125287648	Intron 7		4	T, 0.2199	CC/CT/TT	453/251/36	135.22/138.63/139.38	27.18/28.46/28.12	0.9232	0.5699	0.1056	0.5748
p62140	rs838910	125287380	Intron 7		5	T, 0.3047	GG/GT/TT	358/304/72	135.54/139.34/130.23	27.71/28.09/24.10	-0.0755	0.5087	0.8821	0.9654
p62409	rs838911	125287111	Intron 7		5	T, 0.4211	CC/CT/TT	251/346/135	137.46/138/132.71	28.39/28.04/25.39	-0.6245	0.4748	0.1888	0.6940
p62615	rs7138386	125286905	Intron 7		5	C, 0.1137	TT/TC/CC	571/141/11	137.41/132.86/147.97	27.69/26.95/32.74	-0.6495	0.7473	0.3851	0.7761
p63483	rs838912	125286037	Intron 7		7	A, 0.0867	AA/GA/GG	6/119/624	147.92/141.68/136.06	22.36/28.42/27.33	1.8700	0.8230	**0.0234**	0.3972
p64772	rs5888	125284748	Exon 8	Ala350Ala	3a	T, 0.0961	CC/CT/TT	614/130/8	135.6/141.63/150.76	27.57/28.87/21.81	2.0962	0.7888	**0.0080**	0.2918
p64923	rs838915	125284597	Intron 8		5	A, 0.1435	AA/CA/CC	19/179/547	128.24/137.41/136.97	24.55/26.33/28.38	-0.3684	0.6595	0.5766	0.8571
p65999	rs12819677	125283521	Intron 8		6	A, 0.2813	GG/GA/AA	375/309/52	135.65/137.67/140.14	26.43/28.07/31.39	0.6769	0.5339	0.2052	0.7157
p67439	rs961170	125282081	Intron 8		4	A, 0.0893	AA/GA/GG	11/108/615	127.38/135.18/137.19	30.33/28.13/27.62	-0.9808	0.8009	0.2211	0.7435
p67700	rs1726374	125281820	Intron 8		7	A, 0.1933	GG/GA/AA	500/226/32	136.28/137.42/137.84	27.77/28.32/24.61	0.3249	0.5867	0.5798	0.8571
p69013	rs7135117	125280507	Intron 8		7	G, 0.2901	GG/AG/AA	74/275/383	139.15/137.26/135.9	27.28/28.32/27.33	0.5010	0.5031	0.3196	0.7761
p69699	rs10396210	125279821	Intron 8-splice site		4	A, 0.1511	AA/GA/GG	17/188/527	133.13/133.32/138.01	24.50/25.99/28.45	-1.2921	0.6639	0.0520	0.5052
p69995 delC	rs5801571	125279525	Intron 9		5	delC, 0.2761	DD/WD/WW	63/282/394	138.36/136.65/136.08	29.51/27.84/27.69	0.2920	0.5230	0.5768	0.8571
p71867	rs7954022	125277653	Intron 9		5	T, 0.1323	TT/CT/CC	12/172/556	138.36/139.03/136.02	28.42/27.41/27.66	0.8502	0.6988	0.2241	0.7435
p72197	rs838861	125277323	Intron 9		7	G, 0.3777	AA/AG/GG	304/310/122	138.19/135.01/138.6	28.51/26.54/28.32	-0.1507	0.4657	0.7464	0.9144
p72777	rs838862	125276743	Intron 9		5	T, 0.0887	CC/CT/TT	611/116/7	136.49/140.05/130.13	27.50/27.25/33.12	0.7012	0.8218	0.3938	0.7762
p75766	rs838866	125273754	Intron 9		6	C, 0.2116	TT/TC/CC	466/240/37	136.81/137.6/134.32	27.72/27.49/29.38	-0.0497	0.5699	0.9306	0.9887
p75778	rs7301120	125273742	Intron 9		6	T, 0.1135	TT/CT/CC	9/146/569	153.8/135.98/136.84	34.57/28.15/27.34	0.3767	0.7539	0.6174	0.8839
p76757	rs9919713	125272763	Intron 9		6	T, 0.4390	AA/AT/TT	243/353/148	137.25/137.33/135.86	29.00/27.02/27.54	-0.1860	0.4696	0.6921	0.9051
p77251	rs34339961	125272269	Intron 9		6	T, 0.1177	AA/AT/TT	573/150/11	136.68/136.84/153.06	27.85/28.26/33.32	0.7120	0.7432	0.3383	0.7761
p77842	rs2272310	125271678	Intron 10		5	A, 0.0807	AA/GA/GG	5/110/642	136.12/139.14/136.1	4.96/29.15/27.59	0.8339	0.8661	0.3359	0.7761
p78402	rs838898	125271118	Intron 10		5	A, 0.0714	AA/GA/GG	7/86/601	124.15/135.76/137.49	33.52/24.94/28.40	-0.9806	0.9240	0.2889	0.7761
p78430	rs838897	125271090	Intron 10		5	G, 0.3830	GG/CG/CC	121/315/299	135.15/137.21/136.75	28.01/28.01/27.48	-0.1887	0.4709	0.6887	0.9051
p78747	rs2293440	125270773	Intron 11		5	C, 0.4112	CC/TC/TT	126/348/258	136.06/136.3/137.79	27.37/26.42/29.64	-0.2984	0.4810	0.5352	0.8490
p79721	rs838896	125269799	Intron 11		5	C, 0.3104	GG/GC/CC	357/324/71	135.09/137.26/143.1	28.43/26.98/24.61	1.1147	0.5056	**0.0278**	0.4197
p79828	rs838895	125269692	Intron 11		5	G, 0.3171	GG/CG/CC	73/325/345	142.03/137.79/134.49	25.40/26.62/28.83	1.2206	0.5074	**0.0164**	0.3186
p80045	rs838893	125269475	Intron 11		5	A, 0.3244	GG/GA/AA	344/328/80	135.11/137.56/140.43	29.04/26.78/25.93	0.8859	0.5009	0.0774	0.5540
p83547	rs838887	125265973	Intron 12		5	G, 0.4564	CC/CG/GG	227/358/157	136.15/137.97/135.3	25.51/27.56/30.61	-0.1113	0.4676	0.8119	0.9518
p83884	rs701106	125265636	Intron 12		5	T, 0.2597	TT/CT/CC	51/291/412	142.85/138.67/134.71	28.78/29.50/26.27	1.2967	0.5352	**0.0156**	0.3186

p86276	rs747155	125263244	Intron 12		2b	T, 0.1495	TT/CT/CC	17/191/541	131.8/136.97/136.81	35.42/26.39/28.03	-0.2164	0.6605	0.7433	0.9144
p86481	rs701103	125263039	Exon 13-3' UTR	Gly499Arg (isoform 2)	5	A, 0.2451	AA/GA/GG	50/265/428	132.37/138.77/136.53	27.64/27.23/28.05	-0.0074	0.5400	0.9891	0.9965
p87011	rs58032386	125262509	Exon 13-3' UTR		2a	T, 0.1417	CC/CT/TT	551/186/14	137.07/137/129.91	28.90/24.33/24.12	-0.2769	0.6776	0.6829	0.9051
p87723	rs838881	125261797	3' flanking		6	T, 0.3183	TT/CT/CC	65/344/338	134.34/137.04/136.76	28.25/27.36/28.31	-0.1897	0.5263	0.7187	0.9136
p87749	rs76465225	125261771	3' flanking		7	A, 0.0844	AA/GA/GG	7/111/629	128.6/137.32/136.7	23.34/27.81/27.96	-0.1205	0.8434	0.8865	0.9654
p87927	rs838880	125261593	3' flanking		5	A, 0.2414	AA/GA/GG	40/280/423	134.92/136.85/137.12	29.87/27.16/28.26	-0.2130	0.5636	0.7056	0.9136
**MAF between 1-5%**														
p1316	rs10396208	125348204	Exon 1	Cys21Cys	2b	T, 0.0476	CC/CT/TT	657/60/4	136.18/145.72/126.27	27.33/32.28/23.99	2.0253	1.0962	0.0651	0.5206
p1419	rs201717369	125348101	Intron 1		4	A, 0.0121	GA/GG	15/713	143.53/136.72	33.66/27.75	2.1508	2.3880	0.3681	0.7761
p7650	rs11615630	125341870	Intron 1		5	A, 0.0436	GG/GA	695/68	137.43/130.27	27.44/30.31	-2.4339	1.1578	**0.0359**	0.4359
p45627	rs12297372	125303893	Intron 1		5	G, 0.0487	GG/AG/AA	1/71/661	132.07/139.72/136.41	NA/29.73/27.51	0.9720	1.1101	0.3816	0.7761
p46964	rs114061302	125302556	Intron 1		4	A, 0.0388	AA/GA/GG	1/58/696	92.79/139.86/136.49	NA/31.62/27.47	0.4839	1.2105	0.6895	0.9051
p50118	rs58710319	125299402	Intron 3		5	T, 0.0208	CT/CC	32/719	146.19/136.45	32.51/27.51	3.1376	1.6463	0.0571	0.5173
p50380	rs141748317	125299140	Intron 3		2b	G, 0.0112	AA/AG	727/17	136.64/137.87	27.67/30.07	0.3920	2.2372	0.8609	0.9654
p50489	rs61320152	125299031	Intron 3		4	T, 0.0257	GG/GT	704/39	136.63/143.42	27.39/31.34	2.1813	1.4908	0.1439	0.6311
p54445	rs60910935	125295075	Intron 5		4	G, 0.0418	AA/AG/GG	656/57/2	137.28/132.79/148.43	28.47/22.46/21.21	-0.9880	1.1918	0.4074	0.7915
p54475	rs60227139	125295045	Intron 5		4	T, 0.0437	CC/CT/TT	676/61/2	137.11/133.52/148.4	28.23/22.68/21.21	-0.7550	1.1492	0.5114	0.8404
p77682	rs150082885	125271838	Intron 10		5	G, 0.0106	AA/AG/GG	721/14/1	137.13/122.98/156.01	27.77/29.30/NA	-2.9877	2.1744	0.1698	0.6416
p78791	rs75289200	125270729	Intron 11		5	C, 0.0321	TC/TT	46/685	147.19/136.06	27.99/27.68	3.6568	1.3876	**0.0086**	0.2918
p82019	rs838890	125267501	Intron 11		5	T, 0.0320	CC/CT/TT	690/41/2	137.12/135.58/112.02	27.74/28.19/72.83	-1.1864	1.3456	0.3782	0.7761
p82340	rs77483223	125267180	Intron 12		5	A, 0.0231	GA/GG	35/705	132.2/137.01	25.03/27.96	-1.5601	1.5901	0.3268	0.7761
p82434	rs838889	125267086	Intron 12		5	C, 0.0315	CC/TC/TT	2/41/702	112.08/136.37/136.99	72.83/26.99/27.63	-0.9096	1.3374	0.4967	0.8339
p86245	rs188375019	125263275	Intron 12		4	T, 0.0341	CC/CT	696/50	136.54/142.03	27.69/26.72	1.8399	1.3314	0.1674	0.6416
p86316	rs701104	125263204	Intron 12		4	T, 0.0487	TT/GT/GG	2/64/658	128.07/135.86/137.43	50.49/29.05/27.70	-0.6627	1.1303	0.5579	0.8571
p86967	rs187492239	125262553	Exon 13-3' UTR		4	G, 0.0355	AA/AG	692/52	136.5/140.59	27.82/26.49	1.3818	1.3114	0.2924	0.7761
p87611	rs190688220	125261909	3' flanking		4	T, 0.0316	CC/CT	697/46	136.41/141.77	27.77/27.47	1.7851	1.3894	0.1993	0.7132
p87681	rs838883	125261839	3' flanking		5	A, 0.0459	AA/GA/GG	1/62/659	163.53/136.09/137.17	NA/28.31/27.91	-0.0535	1.1812	0.9639	0.9931
**MAF ≤1%**														
p1048 insC (1048_ 1049)		125348472	Exon 1-5' UTR		2a	insC, 0.0079	WI/WW	12/729	134.79/136.97	31.94/27.67	-0.7733	2.6557	0.7710	0.9144
p49759	rs146272788	125299761	Intron 2		5	T, 0.0020	CC/CT	731/3	137.03/141.39	27.75/17.56	1.5883	5.2643	0.7630	0.9144

p49978	rs5891	125299542	Exon 3	Val135Ile	5	A, 0.0058	GA/GG	9/754	154.31/136.52	39.14/27.50	5.6762	3.0464	0.0628	0.5206
p50024	rs368880622	125299496	Intron 3		5	T, 0.0026	GG/GT	742/4	136.83/142.91	27.51/57.60	1.6012	4.5586	0.7255	0.9136
p50954		125298566	Intron 4		5	C, 0.0007	TC/TT	1/742	110.14/136.79	NA/27.75	-8.7626	9.1297	0.3375	0.7761
p52919		125296601	Intron 4		5	T, 0.0013	GG/GT	741/2	136.81/97.42	27.74/18.38	-13.4137	6.4689	**0.0385**	0.4359
p52995	rs113910315	125296525	Intron 4-splice site		5	G, 0.0020	TG/TT	3/746	138.8/136.77	34.93/27.70	0.6563	5.2739	0.9010	0.9725
p53372	rs115604379	125296148	Intron 5		5	T, 0.0066	CC/CT	735/10	136.57/149.32	27.83/17.13	4.3130	2.9092	0.1386	0.6311
p54611		125294909	Intron 5		4	C, 0.0007	TC/TT	1/748	80.62/136.83	NA/27.66	-19.2831	9.0970	**0.0344**	0.4359
p54627		125294893	Intron 5		4	C, 0.0020	GC/GG	3/744	147.45/136.54	23.34/27.76	3.6910	5.2836	0.4850	0.8255
p54856		125294664	Intron 6		4	T, 0.0007	CC/CT	748/1	136.87/67.98	27.61/NA	-24.0757	9.0781	**0.0082**	0.2918
p57004	rs187562853	125292516	Intron 6		4	A, 0.0098	GG/GA	731/15	136.33/146.22	27.82/26.41	3.2853	2.3865	0.1690	0.6416
p77181	rs146246031	125272339	Intron 9		7	C, 0.0053	TC/TT	8/734	138.87/137.05	40.35/27.46	0.4564	3.2210	0.8874	0.9654
p77381	rs138499966	125272139	Intron 9		6	C, 0.0046	TC/TT	7/741	135.79/136.78	22.32/27.79	-0.2236	3.4610	0.9485	0.9923
p77620	rs377124254	125271900	Intron 10		5	A, 0.0007	GA/GG	1/741	181.07/136.69	NA/27.81	14.4685	9.1465	0.1141	0.5748
p77704		125271816	Intron 10		5	A, 0.0040	CA/CC	6/731	127.91/136.87	31.82/27.82	-3.0744	3.7716	0.4152	0.7954
p78255	rs184052375	125271265	Intron 10		4	G, 0.0072	AA/AG	740/9	136.63/147.01	27.63/34.72	3.3097	3.0552	0.2790	0.7761
p81863	rs185445624	125267657	Intron 11		5	A, 0.0020	GA/GG	3/745	129.03/136.84	26.05/27.73	-2.5166	5.2725	0.6333	0.8972
p82264	rs141545424	125267256	Exon 12	Gly501Gly	5	A, 0.0007	CA/CC	1/745	181.22/136.74	NA/27.71	14.4986	9.1095	0.1119	0.5748
p82369	rs75446635	125267151	Intron 12		5	A, 0.0059	GA/GG	9/739	144.2/136.77	41.63/27.47	2.2239	3.0489	0.4660	0.8230
p87266	rs150512235	125262254	Exon 13-3' UTR[f]		4	C, 0.0057	TC/TT	9/756	137.13/136.56	38.11/27.67	0.0318	3.0667	0.9917	0.9965
p87694		125261826	3' flanking		5	T, 0.0020	CC/CT	731/3	136.86/145.93	27.92/24.62	3.0751	5.3006	0.5620	0.8571

ApoA-I, apolipoprotein A-I; del/D, deletion; FDR, false discovery rate; ins/I, insertion; MA, minor allele; MAF, minor allele frequency; NA, not analyzed; RegDB, RegulomeDB score; SD, standard deviation; SE, standard error; SNP, single nucleotide polymorphism; UTR, untranslated region; W, wild type allele for insertion or deletion on RefSeq.
All alleles on the reverse strand. Splice site is defined as ± 20 bp from the start or end of an exon.
All 10 novel variants identified in this study have been submitted to dbSNP database (batch ID: SCARB1_AB): http://www.ncbi.nlm.nih.gov/SNP/snp_viewTable.cgi?handle=KAMBOH.
ApoA-I values were Box-Cox transformed. Results were adjusted for covariates: sex and age.
Nominally significant *P*-values (*P* <0.05) and FDR values that passed the threshold (FDR <0.20) are shown in **bold**.
[a, c] RefSeq of *SCARB1*: hg19, NM_005505 (CHIP Bioinformatics).
[b] dbSNP build 139: GRCh37.p10.
[d] The RegulomeDB (version 1.0) scoring scheme is described at the footnote of Additional file 4 Table S4 or can be seen at http://regulome.stanford.edu/help.
[e] Corresponding to a *q*-value from Benjamini-Hochberg procedure.
[f] Close to a miRNA-145 seed site based on TargetScanHuman (version 6.2, http://www.targetscan.org/).
