# Supplementary material for: Genetic contribution of SCARB1 variants to lipid traits in African Blacks: a candidate gene association study
Source: BMC Med Genet. 2015 Nov 12;16:106. doi: 10.1186/s12881-015-0250-6 (PMC4643515; doi:10.1186/s12881-015-0250-6)
Supplement: Additional file 16: Table S11. — Haplotype association results for 136 SCARB1 genotyped variants for HDL-C and ApoA-I. (PDF 257 kb) [file 12881_2015_250_MOESM16_ESM.pdf]

Table S11. Haplotype association results for 136 *SCARB1* genotyped variants for HDL-C and ApoA-I.

						HDL-C				ApoA-I			
wind	wind SNP	SNP Name[a]-SNP ID[b] /Chr12 Position[c]	Location	MA, MAF	Genotype	Genotype Count	β	Single-site *P*	Global *P*	Genotype Count	β	Single-site *P*	Global *P*
1	SNP 1	p1048insC-chr12_125348472	Exon 1-5' UTR	insC, 0.0079	WI/WW	12/723	-0.9389	0.3785	0.3506	12/729	-0.7733	0.7710	0.1160
1	SNP 2	p1265-rs2070242	Exon 1	T, 0.1284	CC/CT/TT	541/163/10	-0.5040	0.0833		545/165/10	-1.2535	0.0862	
1	SNP 3	p1316-rs10396208	Exon 1	T, 0.0476	CC/CT/TT	652/58/4	0.2987	0.5001		657/60/4	2.0253	0.0651	
1	SNP 4	p1419-rs201717369	Intron 1	A, 0.0121	AA/GA/GG	1/14/707	0.4892	0.5748		0/15/713	2.1508	0.3681	
2	SNP 1	p1265-rs2070242	Exon 1	T, 0.1284	CC/CT/TT	541/163/10	-0.5040	0.0833	0.6456	545/165/10	-1.2535	0.0862	0.1454
2	SNP 2	p1316-rs10396208	Exon 1	T, 0.0476	CC/CT/TT	652/58/4	0.2987	0.5001		657/60/4	2.0253	0.0651	
2	SNP 3	p1419-rs201717369	Intron 1	A, 0.0121	AA/GA/GG	1/14/707	0.4892	0.5748		0/15/713	2.1508	0.3681	
2	SNP 4	p4012-rs7139401	Intron 1	C, 0.4386	TT/TC/CC	232/343/147	0.0877	0.6453		236/350/148	-0.0802	0.8654	
3	SNP 1	p1316-rs10396208	Exon 1	T, 0.0476	CC/CT/TT	652/58/4	0.2987	0.5001	0.8268	657/60/4	2.0253	0.0651	0.2992
3	SNP 2	p1419-rs201717369	Intron 1	A, 0.0121	AA/GA/GG	1/14/707	0.4892	0.5748		0/15/713	2.1508	0.3681	
3	SNP 3	p4012-rs7139401	Intron 1	C, 0.4386	TT/TC/CC	232/343/147	0.0877	0.6453		236/350/148	-0.0802	0.8654	
3	SNP 4	p5055-rs11057869	Intron 1	A, 0.074	AA/GA/GG	7/96/638	0.0976	0.7828		7/95/650	0.5638	0.5247	
4	SNP 1	p1419-rs201717369	Intron 1	A, 0.0121	AA/GA/GG	1/14/707	0.4892	0.5748	0.6474	0/15/713	2.1508	0.3681	0.7873
4	SNP 2	p4012-rs7139401	Intron 1	C, 0.4386	TT/TC/CC	232/343/147	0.0877	0.6453		236/350/148	-0.0802	0.8654	
4	SNP 3	p5055-rs11057869	Intron 1	A, 0.074	AA/GA/GG	7/96/638	0.0976	0.7828		7/95/650	0.5638	0.5247	
4	SNP 4	p6600-rs12831105	Intron 1	T, 0.1188	CC/CT/TT	566/151/11	0.3570	0.2239		571/152/10	-0.5681	0.4412	
5	SNP 1	p4012-rs7139401	Intron 1	C, 0.4386	TT/TC/CC	232/343/147	0.0877	0.6453	0.6759	236/350/148	-0.0802	0.8654	0.2798
5	SNP 2	p5055-rs11057869	Intron 1	A, 0.074	AA/GA/GG	7/96/638	0.0976	0.7828		7/95/650	0.5638	0.5247	
5	SNP 3	p6600-rs12831105	Intron 1	T, 0.1188	CC/CT/TT	566/151/11	0.3570	0.2239		571/152/10	-0.5681	0.4412	
5	SNP 4	p7650-rs11615630	Intron 1	A, 0.0436	GG/GA	685/67	0.3382	0.4702		695/68	-2.4339	0.0359	
6	SNP 1	p5055-rs11057869	Intron 1	A, 0.074	AA/GA/GG	7/96/638	0.0976	0.7828	0.7353	7/95/650	0.5638	0.5247	0.3274
6	SNP 2	p6600-rs12831105	Intron 1	T, 0.1188	CC/CT/TT	566/151/11	0.3570	0.2239		571/152/10	-0.5681	0.4412	
6	SNP 3	p7650-rs11615630	Intron 1	A, 0.0436	GG/GA	685/67	0.3382	0.4702		695/68	-2.4339	0.0359	
6	SNP 4	p10292-rs4765181	Intron 1	T, 0.249	TT/GT/GG	44/285/417	0.1348	0.5405		44/287/426	-0.0286	0.9583	
7	SNP 1	p6600-rs12831105	Intron 1	T, 0.1188	CC/CT/TT	566/151/11	0.3570	0.2239	0.4139	571/152/10	-0.5681	0.4412	0.1691
7	SNP 2	p7650-rs11615630	Intron 1	A, 0.0436	GG/GA	685/67	0.3382	0.4702		695/68	-2.4339	0.0359	
7	SNP 3	p10292-rs4765181	Intron 1	T, 0.249	TT/GT/GG	44/285/417	0.1348	0.5405		44/287/426	-0.0286	0.9583	
7	SNP 4	p10991-rs10773112	Intron 1	A, 0.3534	GG/GA/AA	303/341/89	0.2146	0.2860		309/339/90	0.4896	0.3230	
8	SNP 1	p7650-rs11615630	Intron 1	A, 0.0436	GG/GA	685/67	0.3382	0.4702	0.2071	695/68	-2.4339	0.0359	0.1742
8	SNP 2	p10292-rs4765181	Intron 1	T, 0.249	TT/GT/GG	44/285/417	0.1348	0.5405		44/287/426	-0.0286	0.9583	
8	SNP 3	p10991-rs10773112	Intron 1	A, 0.3534	GG/GA/AA	303/341/89	0.2146	0.2860		309/339/90	0.4896	0.3230	
8	SNP 4	p13570-rs11057864	Intron 1	T, 0.118	TT/GT/GG	14/147/581	-0.0393	0.8917		15/147/591	0.3006	0.6725	
9	SNP 1	p10292-rs4765181	Intron 1	T, 0.249	TT/GT/GG	44/285/417	0.1348	0.5405	0.6128	44/287/426	-0.0286	0.9583	0.5689
9	SNP 2	p10991-rs10773112	Intron 1	A, 0.3534	GG/GA/AA	303/341/89	0.2146	0.2860		309/339/90	0.4896	0.3230	
9	SNP 3	p13570-rs11057864	Intron 1	T, 0.118	TT/GT/GG	14/147/581	-0.0393	0.8917		15/147/591	0.3006	0.6725	
9	SNP 4	p16565-rs10773111	Intron 1	A, 0.1928	GG/GA/AA	481/239/24	0.1089	0.6550		486/246/23	0.4484	0.4608	
10	SNP 1	p10991-rs10773112	Intron 1	A, 0.3534	GG/GA/AA	303/341/89	0.2146	0.2860	0.5086	309/339/90	0.4896	0.3230	0.8093
10	SNP 2	p13570-rs11057864	Intron 1	T, 0.118	TT/GT/GG	14/147/581	-0.0393	0.8917		15/147/591	0.3006	0.6725	
10	SNP 3	p16565-rs10773111	Intron 1	A, 0.1928	GG/GA/AA	481/239/24	0.1089	0.6550		486/246/23	0.4484	0.4608	
10	SNP 4	p20207-rs11057853	Intron 1	A, 0.4484	AA/GA/GG	150/360/228	0.4082	0.0343		153/366/230	0.8422	0.0751	
11	SNP 1	p13570-rs11057864	Intron 1	T, 0.118	TT/GT/GG	14/147/581	-0.0393	0.8917	0.4147	15/147/591	0.3006	0.6725	0.4335
11	SNP 2	p16565-rs10773111	Intron 1	A, 0.1928	GG/GA/AA	481/239/24	0.1089	0.6550		486/246/23	0.4484	0.4608	
11	SNP 3	p20207-rs11057853	Intron 1	A, 0.4484	AA/GA/GG	150/360/228	0.4082	0.0343		153/366/230	0.8422	0.0751	
11	SNP 4	p20694-rs11057852	Intron 1	A, 0.105	GG/GA/AA	581/130/11	-0.0308	0.9198		587/129/11	0.3519	0.6452	
12	SNP 1	p16565-rs10773111	Intron 1	A, 0.1928	GG/GA/AA	481/239/24	0.1089	0.6550	0.0764	486/246/23	0.4484	0.4608	0.2988
12	SNP 2	p20207-rs11057853	Intron 1	A, 0.4484	AA/GA/GG	150/360/228	0.4082	0.0343		153/366/230	0.8422	0.0751	
12	SNP 3	p20694-rs11057852	Intron 1	A, 0.105	GG/GA/AA	581/130/11	-0.0308	0.9198		587/129/11	0.3519	0.6452	
12	SNP 4	p20741-rs11057851	Intron 1	T, 0.3237	TT/CT/CC	72/334/328	-0.5924	0.0043		73/336/337	-1.2331	0.0162	
13	SNP 1	p20207-rs11057853	Intron 1	A, 0.4484	AA/GA/GG	150/360/228	0.4082	0.0343	0.0762	153/366/230	0.8422	0.0751	0.0931
13	SNP 2	p20694-rs11057852	Intron 1	A, 0.105	GG/GA/AA	581/130/11	-0.0308	0.9198		587/129/11	0.3519	0.6452	
13	SNP 3	p20741-rs11057851	Intron 1	T, 0.3237	TT/CT/CC	72/334/328	-0.5924	0.0043		73/336/337	-1.2331	0.0162	
13	SNP 4	p21145-rs3924313	Intron 1	T, 0.1772	CC/CT/TT	503/219/22	0.1488	0.5502		511/221/23	0.1215	0.8437	

14	SNP 1	p20694-rs11057852	Intron 1	A, 0.105	GG/GA/AA	581/130/11	-0.0308	0.9198	0.0886	587/129/11	0.3519	0.6452	0.1116
14	SNP 2	p20741-rs11057851	Intron 1	T, 0.3237	TT/CT/CC	72/334/328	-0.5924	0.0043		73/336/337	-1.2331	0.0162	
14	SNP 3	p21145-rs3924313	Intron 1	T, 0.1772	CC/CT/TT	503/219/22	0.1488	0.5502		511/221/23	0.1215	0.8437	
14	SNP 4	p22116-rs12370382	Intron 1	A, 0.0645	GG/GA/AA	643/86/3	0.1369	0.7300		650/89/3	0.5264	0.5882	
15	SNP 1	p20741-rs11057851	Intron 1	T, 0.3237	TT/CT/CC	72/334/328	-0.5924	0.0043	0.1004	73/336/337	-1.2331	0.0162	0.1461
15	SNP 2	p21145-rs3924313	Intron 1	T, 0.1772	CC/CT/TT	503/219/22	0.1488	0.5502		511/221/23	0.1215	0.8437	
15	SNP 3	p22116-rs12370382	Intron 1	A, 0.0645	GG/GA/AA	643/86/3	0.1369	0.7300		650/89/3	0.5264	0.5882	
15	SNP 4	p22168-rs7137797	Intron 1	C, 0.3977	CC/TC/TT	113/355/259	0.2153	0.2763		112/358/262	0.5066	0.3003	
16	SNP 1	p21145-rs3924313	Intron 1	T, 0.1772	CC/CT/TT	503/219/22	0.1488	0.5502	0.4920	511/221/23	0.1215	0.8437	0.7203
16	SNP 2	p22116-rs12370382	Intron 1	A, 0.0645	GG/GA/AA	643/86/3	0.1369	0.7300		650/89/3	0.5264	0.5882	
16	SNP 3	p22168-rs7137797	Intron 1	C, 0.3977	CC/TC/TT	113/355/259	0.2153	0.2763		112/358/262	0.5066	0.3003	
16	SNP 4	p22331-rs6488944	Intron 1	G, 0.1474	TT/TG/GG	522/188/12	0.3091	0.2661		524/191/12	0.3684	0.5936	
17	SNP 1	p22116-rs12370382	Intron 1	A, 0.0645	GG/GA/AA	643/86/3	0.1369	0.7300	0.2834	650/89/3	0.5264	0.5882	0.6519
17	SNP 2	p22168-rs7137797	Intron 1	C, 0.3977	CC/TC/TT	113/355/259	0.2153	0.2763		112/358/262	0.5066	0.3003	
17	SNP 3	p22331-rs6488944	Intron 1	G, 0.1474	TT/TG/GG	522/188/12	0.3091	0.2661		524/191/12	0.3684	0.5936	
17	SNP 4	p22675-rs12425134	Intron 1	T, 0.0526	TT/GT/GG	2/75/662	0.7556	0.0738		2/74/668	1.1151	0.2900	
18	SNP 1	p22168-rs7137797	Intron 1	C, 0.3977	CC/TC/TT	113/355/259	0.2153	0.2763	0.2862	112/358/262	0.5066	0.3003	0.7096
18	SNP 2	p22331-rs6488944	Intron 1	G, 0.1474	TT/TG/GG	522/188/12	0.3091	0.2661		524/191/12	0.3684	0.5936	
18	SNP 3	p22675-rs12425134	Intron 1	T, 0.0526	TT/GT/GG	2/75/662	0.7556	0.0738		2/74/668	1.1151	0.2900	
18	SNP 4	p28137-rs12229555	Intron 1	G, 0.3896	GG/AG/AA	99/373/257	-0.3976	0.0500		100/373/265	-0.5752	0.2524	
19	SNP 1	p22331-rs6488944	Intron 1	G, 0.1474	TT/TG/GG	522/188/12	0.3091	0.2661	0.1757	524/191/12	0.3684	0.5936	0.5933
19	SNP 2	p22675-rs12425134	Intron 1	T, 0.0526	TT/GT/GG	2/75/662	0.7556	0.0738		2/74/668	1.1151	0.2900	
19	SNP 3	p28137-rs12229555	Intron 1	G, 0.3896	GG/AG/AA	99/373/257	-0.3976	0.0500		100/373/265	-0.5752	0.2524	
19	SNP 4	p28692-rs4765622	Intron 1	T, 0.2565	TT/CT/CC	43/289/403	0.1962	0.3817		42/298/406	0.7871	0.1553	
20	SNP 1	p22675-rs12425134	Intron 1	T, 0.0526	TT/GT/GG	2/75/662	0.7556	0.0738	0.1080	2/74/668	1.1151	0.2900	0.2584
20	SNP 2	p28137-rs12229555	Intron 1	G, 0.3896	GG/AG/AA	99/373/257	-0.3976	0.0500		100/373/265	-0.5752	0.2524	
20	SNP 3	p28692-rs4765622	Intron 1	T, 0.2565	TT/CT/CC	43/289/403	0.1962	0.3817		42/298/406	0.7871	0.1553	
20	SNP 4	p28957-rs11057844	Intron 1	A, 0.2362	GG/GA/AA	428/263/40	0.3671	0.1075		433/269/40	0.6107	0.2781	
21	SNP 1	p28137-rs12229555	Intron 1	G, 0.3896	GG/AG/AA	99/373/257	-0.3976	0.0500	0.2221	100/373/265	-0.5752	0.2524	0.2655
21	SNP 2	p28692-rs4765622	Intron 1	T, 0.2565	TT/CT/CC	43/289/403	0.1962	0.3817		42/298/406	0.7871	0.1553	
21	SNP 3	p28957-rs11057844	Intron 1	A, 0.2362	GG/GA/AA	428/263/40	0.3671	0.1075		433/269/40	0.6107	0.2781	
21	SNP 4	p29749-rs10846751	Intron 1	T, 0.4492	TT/CT/CC	142/365/210	-0.3558	0.0701		142/366/215	-0.7763	0.1104	
22	SNP 1	p28692-rs4765622	Intron 1	T, 0.2565	TT/CT/CC	43/289/403	0.1962	0.3817	0.1369	42/298/406	0.7871	0.1553	0.6135
22	SNP 2	p28957-rs11057844	Intron 1	A, 0.2362	GG/GA/AA	428/263/40	0.3671	0.1075		433/269/40	0.6107	0.2781	
22	SNP 3	p29749-rs10846751	Intron 1	T, 0.4492	TT/CT/CC	142/365/210	-0.3558	0.0701		142/366/215	-0.7763	0.1104	
22	SNP 4	p31072-rs10846749	Intron 1	G, 0.4461	CC/CG/GG	222/366/144	-0.3792	0.0510		226/371/146	-0.8005	0.0970	
23	SNP 1	p28957-rs11057844	Intron 1	A, 0.2362	GG/GA/AA	428/263/40	0.3671	0.1075	0.2085	433/269/40	0.6107	0.2781	0.6333
23	SNP 2	p29749-rs10846751	Intron 1	T, 0.4492	TT/CT/CC	142/365/210	-0.3558	0.0701		142/366/215	-0.7763	0.1104	
23	SNP 3	p31072-rs10846749	Intron 1	G, 0.4461	CC/CG/GG	222/366/144	-0.3792	0.0510		226/371/146	-0.8005	0.0970	
23	SNP 4	p31938-rs10744182	Intron 1	A, 0.1837	AA/GA/GG	25/221/487	-0.2885	0.2438		25/226/493	-0.4389	0.4748	
24	SNP 1	p29749-rs10846751	Intron 1	T, 0.4492	TT/CT/CC	142/365/210	-0.3558	0.0701	0.1804	142/366/215	-0.7763	0.1104	0.5858
24	SNP 2	p31072-rs10846749	Intron 1	G, 0.4461	CC/CG/GG	222/366/144	-0.3792	0.0510		226/371/146	-0.8005	0.0970	
24	SNP 3	p31938-rs10744182	Intron 1	A, 0.1837	AA/GA/GG	25/221/487	-0.2885	0.2438		25/226/493	-0.4389	0.4748	
24	SNP 4	p32129-rs10773107	Intron 1	T, 0.1009	TT/GT/GG	9/132/593	-0.3186	0.3107		9/131/605	0.8714	0.2605	
25	SNP 1	p31072-rs10846749	Intron 1	G, 0.4461	CC/CG/GG	222/366/144	-0.3792	0.0510	0.1155	226/371/146	-0.8005	0.0970	0.3437
25	SNP 2	p31938-rs10744182	Intron 1	A, 0.1837	AA/GA/GG	25/221/487	-0.2885	0.2438		25/226/493	-0.4389	0.4748	
25	SNP 3	p32129-rs10773107	Intron 1	T, 0.1009	TT/GT/GG	9/132/593	-0.3186	0.3107		9/131/605	0.8714	0.2605	
25	SNP 4	p32273-rs12580803	Intron 1	C, 0.1006	TT/TC/CC	619/114/18	0.2010	0.4984		627/117/18	1.2550	0.0842	
26	SNP 1	p31938-rs10744182	Intron 1	A, 0.1837	AA/GA/GG	25/221/487	-0.2885	0.2438	0.9308	25/226/493	-0.4389	0.4748	0.1939
26	SNP 2	p32129-rs10773107	Intron 1	T, 0.1009	TT/GT/GG	9/132/593	-0.3186	0.3107		9/131/605	0.8714	0.2605	
26	SNP 3	p32273-rs12580803	Intron 1	C, 0.1006	TT/TC/CC	619/114/18	0.2010	0.4984		627/117/18	1.2550	0.0842	
26	SNP 4	p32290-rs10744181	Intron 1	C, 0.1238	TT/TC/CC	547/141/18	-0.1852	0.5162		551/143/18	0.1584	0.8211	
27	SNP 1	p32129-rs10773107	Intron 1	T, 0.1009	TT/GT/GG	9/132/593	-0.3186	0.3107	0.7762	9/131/605	0.8714	0.2605	0.5428
27	SNP 2	p32273-rs12580803	Intron 1	C, 0.1006	TT/TC/CC	619/114/18	0.2010	0.4984		627/117/18	1.2550	0.0842	
27	SNP 3	p32290-rs10744181	Intron 1	C, 0.1238	TT/TC/CC	547/141/18	-0.1852	0.5162		551/143/18	0.1584	0.8211	
27	SNP 4	p32395-rs12581963	Intron 1	T, 0.1314	TT/CT/CC	11/179/557	-0.3173	0.2589		11/177/570	-0.6483	0.3556	
28	SNP 1	p32273-rs12580803	Intron 1	C, 0.1006	TT/TC/CC	619/114/18	0.2010	0.4984	0.6853	627/117/18	1.2550	0.0842	0.8033
28	SNP 2	p32290-rs10744181	Intron 1	C, 0.1238	TT/TC/CC	547/141/18	-0.1852	0.5162		551/143/18	0.1584	0.8211	
28	SNP 3	p32395-rs12581963	Intron 1	T, 0.1314	TT/CT/CC	11/179/557	-0.3173	0.2589		11/177/570	-0.6483	0.3556	
28	SNP 4	p32750-rs7967521	Intron 1	G, 0.3425	GG/AG/AA	89/306/310	-0.2137	0.2936		91/304/315	0.0564	0.9104	
29	SNP 1	p32290-rs10744181	Intron 1	C, 0.1238	TT/TC/CC	547/141/18	-0.1852	0.5162	0.3363	551/143/18	0.1584	0.8211	0.8929
29	SNP 2	p32395-rs12581963	Intron 1	T, 0.1314	TT/CT/CC	11/179/557	-0.3173	0.2589		11/177/570	-0.6483	0.3556	
29	SNP 3	p32750-rs7967521	Intron 1	G, 0.3425	GG/AG/AA	89/306/310	-0.2137	0.2936		91/304/315	0.0564	0.9104	

29	SNP 4	p32777-rs11057841	Intron 1	A, 0.2805	GG/GA/AA	367/307/50	-0.1217	0.5781		374/303/52	0.0373	0.9450	
30	SNP 1	p32395-rs12581963	Intron 1	T, 0.1314	TT/CT/CC	11/179/557	-0.3173	0.2589	0.3979	11/177/570	-0.6483	0.3556	0.9607
30	SNP 2	p32750-rs7967521	Intron 1	G, 0.3425	GG/AG/AA	89/306/310	-0.2137	0.2936		91/304/315	0.0564	0.9104	
30	SNP 3	p32777-rs11057841	Intron 1	A, 0.2805	GG/GA/AA	367/307/50	-0.1217	0.5781		374/303/52	0.0373	0.9450	
30	SNP 4	p32860-rs7967406	Intron 1	C, 0.0991	CC/AC/AA	9/131/608	0.2647	0.3996		9/135/616	0.4768	0.5369	
31	SNP 1	p32750-rs7967521	Intron 1	G, 0.3425	GG/AG/AA	89/306/310	-0.2137	0.2936	0.3062	91/304/315	0.0564	0.9104	0.9880
31	SNP 2	p32777-rs11057841	Intron 1	A, 0.2805	GG/GA/AA	367/307/50	-0.1217	0.5781		374/303/52	0.0373	0.9450	
31	SNP 3	p32860-rs7967406	Intron 1	C, 0.0991	CC/AC/AA	9/131/608	0.2647	0.3996		9/135/616	0.4768	0.5369	
31	SNP 4	p33531-rs11057838	Intron 1	A, 0.2278	CC/CA/AA	439/254/41	-0.2064	0.3609		440/259/40	0.1993	0.7218	
32	SNP 1	p32777-rs11057841	Intron 1	A, 0.2805	GG/GA/AA	367/307/50	-0.1217	0.5781	0.3039	374/303/52	0.0373	0.9450	0.9957
32	SNP 2	p32860-rs7967406	Intron 1	C, 0.0991	CC/AC/AA	9/131/608	0.2647	0.3996		9/135/616	0.4768	0.5369	
32	SNP 3	p33531-rs11057838	Intron 1	A, 0.2278	CC/CA/AA	439/254/41	-0.2064	0.3609		440/259/40	0.1993	0.7218	
32	SNP 4	p36094-rs11608336	Intron 1	A, 0.1543	GG/GA/AA	521/195/15	0.3354	0.2108		526/195/16	-0.0956	0.8848	
33	SNP 1	p32860-rs7967406	Intron 1	C, 0.0991	CC/AC/AA	9/131/608	0.2647	0.3996	0.1952	9/135/616	0.4768	0.5369	0.5389
33	SNP 2	p33531-rs11057838	Intron 1	A, 0.2278	CC/CA/AA	439/254/41	-0.2064	0.3609		440/259/40	0.1993	0.7218	
33	SNP 3	p36094-rs11608336	Intron 1	A, 0.1543	GG/GA/AA	521/195/15	0.3354	0.2108		526/195/16	-0.0956	0.8848	
33	SNP 4	p36361-rs4765178	Intron 1	T, 0.1671	TT/CT/CC	19/201/496	-0.1369	0.6024		18/205/497	0.5087	0.4368	
34	SNP 1	p33531-rs11057838	Intron 1	A, 0.2278	CC/CA/AA	439/254/41	-0.2064	0.3609	0.7481	440/259/40	0.1993	0.7218	0.3649
34	SNP 2	p36094-rs11608336	Intron 1	A, 0.1543	GG/GA/AA	521/195/15	0.3354	0.2108		526/195/16	-0.0956	0.8848	
34	SNP 3	p36361-rs4765178	Intron 1	T, 0.1671	TT/CT/CC	19/201/496	-0.1369	0.6024		18/205/497	0.5087	0.4368	
34	SNP 4	p36908-rs10846745	Intron 1	G, 0.3257	GG/CG/CC	76/334/329	-0.0384	0.8511		78/335/337	0.0089	0.9859	
35	SNP 1	p36094-rs11608336	Intron 1	A, 0.1543	GG/GA/AA	521/195/15	0.3354	0.2108	0.9219	526/195/16	-0.0956	0.8848	0.8447
35	SNP 2	p36361-rs4765178	Intron 1	T, 0.1671	TT/CT/CC	19/201/496	-0.1369	0.6024		18/205/497	0.5087	0.4368	
35	SNP 3	p36908-rs10846745	Intron 1	G, 0.3257	GG/CG/CC	76/334/329	-0.0384	0.8511		78/335/337	0.0089	0.9859	
35	SNP 4	p37095-rs10846744	Intron 1	G, 0.3056	CC/CG/GG	348/340/57	0.1168	0.5829		350/350/56	0.1538	0.7732	
36	SNP 1	p36361-rs4765178	Intron 1	T, 0.1671	TT/CT/CC	19/201/496	-0.1369	0.6024	0.4949	18/205/497	0.5087	0.4368	0.9787
36	SNP 2	p36908-rs10846745	Intron 1	G, 0.3257	GG/CG/CC	76/334/329	-0.0384	0.8511		78/335/337	0.0089	0.9859	
36	SNP 3	p37095-rs10846744	Intron 1	G, 0.3056	CC/CG/GG	348/340/57	0.1168	0.5829		350/350/56	0.1538	0.7732	
36	SNP 4	p41632-rs6488943	Intron 1	C, 0.2954	CC/AC/AA	50/309/337	-0.2195	0.3244		52/312/340	0.3025	0.5783	
37	SNP 1	p36908-rs10846745	Intron 1	G, 0.3257	GG/CG/CC	76/334/329	-0.0384	0.8511	0.8624	78/335/337	0.0089	0.9859	0.7511
37	SNP 2	p37095-rs10846744	Intron 1	G, 0.3056	CC/CG/GG	348/340/57	0.1168	0.5829		350/350/56	0.1538	0.7732	
37	SNP 3	p41632-rs6488943	Intron 1	C, 0.2954	CC/AC/AA	50/309/337	-0.2195	0.3244		52/312/340	0.3025	0.5783	
37	SNP 4	p42467-rs11057830	Intron 1	T, 0.1523	TT/CT/CC	13/201/523	-0.2810	0.3015		14/200/533	-0.9351	0.1612	
38	SNP 1	p37095-rs10846744	Intron 1	G, 0.3056	CC/CG/GG	348/340/57	0.1168	0.5829	0.4622	350/350/56	0.1538	0.7732	0.8715
38	SNP 2	p41632-rs6488943	Intron 1	C, 0.2954	CC/AC/AA	50/309/337	-0.2195	0.3244		52/312/340	0.3025	0.5783	
38	SNP 3	p42467-rs11057830	Intron 1	T, 0.1523	TT/CT/CC	13/201/523	-0.2810	0.3015		14/200/533	-0.9351	0.1612	
38	SNP 4	p45516-rs1902569	Intron 1	A, 0.1544	AA/GA/GG	18/190/519	0.5447	0.0386		18/191/523	0.5127	0.4331	
39	SNP 1	p41632-rs6488943	Intron 1	C, 0.2954	CC/AC/AA	50/309/337	-0.2195	0.3244	**0.0207**	52/312/340	0.3025	0.5783	0.4914
39	SNP 2	p42467-rs11057830	Intron 1	T, 0.1523	TT/CT/CC	13/201/523	-0.2810	0.3015		14/200/533	-0.9351	0.1612	
39	SNP 3	p45516-rs1902569	Intron 1	A, 0.1544	AA/GA/GG	18/190/519	0.5447	0.0386		18/191/523	0.5127	0.4331	
39	SNP 4	p45627-rs12297372	Intron 1	G, 0.0487	GG/AG/AA	1/68/659	-0.0483	0.9156		1/71/661	0.9720	0.3816	
40	SNP 1	p42467-rs11057830	Intron 1	T, 0.1523	TT/CT/CC	13/201/523	-0.2810	0.3015	0.3346	14/200/533	-0.9351	0.1612	0.5468
40	SNP 2	p45516-rs1902569	Intron 1	A, 0.1544	AA/GA/GG	18/190/519	0.5447	0.0386		18/191/523	0.5127	0.4331	
40	SNP 3	p45627-rs12297372	Intron 1	G, 0.0487	GG/AG/AA	1/68/659	-0.0483	0.9156		1/71/661	0.9720	0.3816	
40	SNP 4	p46964-rs114061302	Intron 1	A, 0.0388	AA/GA/GG	1/55/688	0.0526	0.9158		1/58/696	0.4839	0.6895	
41	SNP 1	p45516-rs1902569	Intron 1	A, 0.1544	AA/GA/GG	18/190/519	0.5447	0.0386	0.2665	18/191/523	0.5127	0.4331	0.8055
41	SNP 2	p45627-rs12297372	Intron 1	G, 0.0487	GG/AG/AA	1/68/659	-0.0483	0.9156		1/71/661	0.9720	0.3816	
41	SNP 3	p46964-rs114061302	Intron 1	A, 0.0388	AA/GA/GG	1/55/688	0.0526	0.9158		1/58/696	0.4839	0.6895	
41	SNP 4	p48969-rs2343394	Intron 2	T, 0.1898	TT/CT/CC	32/225/491	0.3165	0.1788		32/226/501	0.6411	0.2714	
42	SNP 1	p45627-rs12297372	Intron 1	G, 0.0487	GG/AG/AA	1/68/659	-0.0483	0.9156	0.6060	1/71/661	0.9720	0.3816	0.8644
42	SNP 2	p46964-rs114061302	Intron 1	A, 0.0388	AA/GA/GG	1/55/688	0.0526	0.9158		1/58/696	0.4839	0.6895	
42	SNP 3	p48969-rs2343394	Intron 2	T, 0.1898	TT/CT/CC	32/225/491	0.3165	0.1788		32/226/501	0.6411	0.2714	
42	SNP 4	p49537-rs7305310	Intron 2	T, 0.1007	CC/CT/TT	595/117/16	-0.3396	0.2566		601/118/16	-0.2265	0.7599	
43	SNP 1	p46964-rs114061302	Intron 1	A, 0.0388	AA/GA/GG	1/55/688	0.0526	0.9158	0.2986	1/58/696	0.4839	0.6895	0.6379
43	SNP 2	p48969-rs2343394	Intron 2	T, 0.1898	TT/CT/CC	32/225/491	0.3165	0.1788		32/226/501	0.6411	0.2714	
43	SNP 3	p49537-rs7305310	Intron 2	T, 0.1007	CC/CT/TT	595/117/16	-0.3396	0.2566		601/118/16	-0.2265	0.7599	
43	SNP 4	p49570delC-rs145376237	Intron 2	delC, 0.2276	DD/WD/WW	36/260/432	0.3121	0.1773		37/260/437	0.4929	0.3880	
44	SNP 1	p48969-rs2343394	Intron 2	T, 0.1898	TT/CT/CC	32/225/491	0.3165	0.1788	**0.0271**	32/226/501	0.6411	0.2714	0.3331
44	SNP 2	p49537-rs7305310	Intron 2	T, 0.1007	CC/CT/TT	595/117/16	-0.3396	0.2566		601/118/16	-0.2265	0.7599	
44	SNP 3	p49570delC-rs145376237	Intron 2	delC, 0.2276	DD/WD/WW	36/260/432	0.3121	0.1773		37/260/437	0.4929	0.3880	
44	SNP 4	p49690-rs4765615	Intron 2	A, 0.4426	AA/GA/GG	156/318/244	-0.4646	0.0130		157/323/244	-0.9139	0.0480	

45	SNP 1	p49537-rs7305310	Intron 2	T, 0.1007	CC/CT/TT	595/117/16	-0.3396	0.2566	**0.0155**	601/118/16	-0.2265	0.7599	0.2586
45	SNP 2	p49570delC-rs145376237	Intron 2	delC, 0.2276	DD/WD/WW	36/260/432	0.3121	0.1773		37/260/437	0.4929	0.3880	
45	SNP 3	p49690-rs4765615	Intron 2	A, 0.4426	AA/GA/GG	156/318/244	-0.4646	0.0130		157/323/244	-0.9139	0.0480	
45	SNP 4	p49759-rs146272788	Intron 2	T, 0.002	CC/CT	725/3	2.5988	0.2219		731/3	1.5883	0.7630	
46	SNP 1	p49570delC-rs145376237	Intron 2	delC, 0.2276	DD/WD/WW	36/260/432	0.3121	0.1773	**0.0278**	37/260/437	0.4929	0.3880	0.0675
46	SNP 2	p49690-rs4765615	Intron 2	A, 0.4426	AA/GA/GG	156/318/244	-0.4646	0.0130		157/323/244	-0.9139	0.0480	
46	SNP 3	p49759-rs146272788	Intron 2	T, 0.002	CC/CT	725/3	2.5988	0.2219		731/3	1.5883	0.7630	
46	SNP 4	p49978-rs5891	Exon 3	A, 0.0058	GA/GG	9/743	1.3374	0.2791		9/754	5.6762	0.0628	
47	SNP 1	p49690-rs4765615	Intron 2	A, 0.4426	AA/GA/GG	156/318/244	-0.4646	0.0130	**0.0079**	157/323/244	-0.9139	0.0480	**0.0343**
47	SNP 2	p49759-rs146272788	Intron 2	T, 0.002	CC/CT	725/3	2.5988	0.2219		731/3	1.5883	0.7630	
47	SNP 3	p49978-rs5891	Exon 3	A, 0.0058	GA/GG	9/743	1.3374	0.2791		9/754	5.6762	0.0628	
47	SNP 4	p50024-rs368880622	Intron 3	T, 0.0026	GG/GT	737/3	1.6506	0.4362		742/4	1.6012	0.7255	
48	SNP 1	p49759-rs146272788	Intron 2	T, 0.002	CC/CT	725/3	2.5988	0.2219	0.1075	731/3	1.5883	0.7630	**0.0293**
48	SNP 2	p49978-rs5891	Exon 3	A, 0.0058	GA/GG	9/743	1.3374	0.2791		9/754	5.6762	0.0628	
48	SNP 3	p50024-rs368880622	Intron 3	T, 0.0026	GG/GT	737/3	1.6506	0.4362		742/4	1.6012	0.7255	
48	SNP 4	p50118-rs58710319	Intron 3	T, 0.0208	CT/CC	31/711	0.7885	0.2399		32/719	3.1376	0.0571	
49	SNP 1	p49978-rs5891	Exon 3	A, 0.0058	GA/GG	9/743	1.3374	0.2791	0.0672	9/754	5.6762	0.0628	**0.0289**
49	SNP 2	p50024-rs368880622	Intron 3	T, 0.0026	GG/GT	737/3	1.6506	0.4362		742/4	1.6012	0.7255	
49	SNP 3	p50118-rs58710319	Intron 3	T, 0.0208	CT/CC	31/711	0.7885	0.2399		32/719	3.1376	0.0571	
49	SNP 4	p50151-rs2278986	Intron 3	C, 0.1933	CC/TC/TT	33/225/484	0.4333	0.0656		33/226/494	0.8568	0.1419	
50	SNP 1	p50024-rs368880622	Intron 3	T, 0.0026	GG/GT	737/3	1.6506	0.4362	0.1980	742/4	1.6012	0.7255	0.1897
50	SNP 2	p50118-rs58710319	Intron 3	T, 0.0208	CT/CC	31/711	0.7885	0.2399		32/719	3.1376	0.0571	
50	SNP 3	p50151-rs2278986	Intron 3	C, 0.1933	CC/TC/TT	33/225/484	0.4333	0.0656		33/226/494	0.8568	0.1419	
50	SNP 4	p50380-rs141748317	Intron 3	G, 0.0112	AA/AG	723/15	-0.0273	0.9772		727/17	0.3920	0.8609	
51	SNP 1	p50118-rs58710319	Intron 3	T, 0.0208	CT/CC	31/711	0.7885	0.2399	0.1254	32/719	3.1376	0.0571	0.0779
51	SNP 2	p50151-rs2278986	Intron 3	C, 0.1933	CC/TC/TT	33/225/484	0.4333	0.0656		33/226/494	0.8568	0.1419	
51	SNP 3	p50380-rs141748317	Intron 3	G, 0.0112	AA/AG	723/15	-0.0273	0.9772		727/17	0.3920	0.8609	
51	SNP 4	p50489-rs61320152	Intron 3	T, 0.0257	GG/GT	699/38	0.2390	0.6958		704/39	2.1813	0.1439	
52	SNP 1	p50151-rs2278986	Intron 3	C, 0.1933	CC/TC/TT	33/225/484	0.4333	0.0656	0.2903	33/226/494	0.8568	0.1419	0.1905
52	SNP 2	p50380-rs141748317	Intron 3	G, 0.0112	AA/AG	723/15	-0.0273	0.9772		727/17	0.3920	0.8609	
52	SNP 3	p50489-rs61320152	Intron 3	T, 0.0257	GG/GT	699/38	0.2390	0.6958		704/39	2.1813	0.1439	
52	SNP 4	p50954-chr12_125298566	Intron 4	C, 0.0007	TC/TT	1/735	4.5639	0.2131		1/742	-8.7626	0.3375	
53	SNP 1	p50380-rs141748317	Intron 3	G, 0.0112	AA/AG	723/15	-0.0273	0.9772	0.9605	727/17	0.3920	0.8609	0.5439
53	SNP 2	p50489-rs61320152	Intron 3	T, 0.0257	GG/GT	699/38	0.2390	0.6958		704/39	2.1813	0.1439	
53	SNP 3	p50954-chr12_125298566	Intron 4	C, 0.0007	TC/TT	1/735	4.5639	0.2131		1/742	-8.7626	0.3375	
53	SNP 4	p51888-rs7138304	Intron 4	T, 0.1079	TT/CT/CC	16/129/589	-0.0610	0.8334		16/130/593	-0.0031	0.9965	
54	SNP 1	p50489-rs61320152	Intron 3	T, 0.0257	GG/GT	699/38	0.2390	0.6958	0.8543	704/39	2.1813	0.1439	0.6688
54	SNP 2	p50954-chr12_125298566	Intron 4	C, 0.0007	TC/TT	1/735	4.5639	0.2131		1/742	-8.7626	0.3375	
54	SNP 3	p51888-rs7138304	Intron 4	T, 0.1079	TT/CT/CC	16/129/589	-0.0610	0.8334		16/130/593	-0.0031	0.9965	
54	SNP 4	p52096-rs10846739	Intron 4	G, 0.4693	GG/AG/AA	169/343/217	0.0195	0.9168		172/342/221	-0.1537	0.7377	
55	SNP 1	p50954-chr12_125298566	Intron 4	C, 0.0007	TC/TT	1/735	4.5639	0.2131	0.6351	1/742	-8.7626	0.3375	0.5383
55	SNP 2	p51888-rs7138304	Intron 4	T, 0.1079	TT/CT/CC	16/129/589	-0.0610	0.8334		16/130/593	-0.0031	0.9965	
55	SNP 3	p52096-rs10846739	Intron 4	G, 0.4693	GG/AG/AA	169/343/217	0.0195	0.9168		172/342/221	-0.1537	0.7377	
55	SNP 4	p52556-rs11057820	Intron 4	A, 0.1	AA/GA/GG	12/124/610	-0.1513	0.6235		12/126/619	-1.1588	0.1282	
56	SNP 1	p51888-rs7138304	Intron 4	T, 0.1079	TT/CT/CC	16/129/589	-0.0610	0.8334	0.9587	16/130/593	-0.0031	0.9965	0.3509
56	SNP 2	p52096-rs10846739	Intron 4	G, 0.4693	GG/AG/AA	169/343/217	0.0195	0.9168		172/342/221	-0.1537	0.7377	
56	SNP 3	p52556-rs11057820	Intron 4	A, 0.1	AA/GA/GG	12/124/610	-0.1513	0.6235		12/126/619	-1.1588	0.1282	
56	SNP 4	p52610-rs10846738	Intron 4	T, 0.1349	TT/CT/CC	12/172/545	-0.0102	0.9710		13/172/553	-0.6490	0.3462	
57	SNP 1	p52096-rs10846739	Intron 4	G, 0.4693	GG/AG/AA	169/343/217	0.0195	0.9168	0.5802	172/342/221	-0.1537	0.7377	0.3474
57	SNP 2	p52556-rs11057820	Intron 4	A, 0.1	AA/GA/GG	12/124/610	-0.1513	0.6235		12/126/619	-1.1588	0.1282	
57	SNP 3	p52610-rs10846738	Intron 4	T, 0.1349	TT/CT/CC	12/172/545	-0.0102	0.9710		13/172/553	-0.6490	0.3462	
57	SNP 4	p52919-chr12_125296601	Intron 4	T, 0.0013	GG/GT	734/2	-7.4063	0.0043		741/2	-13.4137	0.0385	
58	SNP 1	p52556-rs11057820	Intron 4	A, 0.1	AA/GA/GG	12/124/610	-0.1513	0.6235	0.2618	12/126/619	-1.1588	0.1282	0.1625
58	SNP 2	p52610-rs10846738	Intron 4	T, 0.1349	TT/CT/CC	12/172/545	-0.0102	0.9710		13/172/553	-0.6490	0.3462	
58	SNP 3	p52919-chr12_125296601	Intron 4	T, 0.0013	GG/GT	734/2	-7.4063	0.0043		741/2	-13.4137	0.0385	
58	SNP 4	p52956-rs77740046	Intron 4	T, 0.0546	CC/CT/TT	651/75/3	0.3300	0.4288		657/75/3	-0.4428	0.6689	
59	SNP 1	p52610-rs10846738	Intron 4	T, 0.1349	TT/CT/CC	12/172/545	-0.0102	0.9710	0.1054	13/172/553	-0.6490	0.3462	0.4723

59	SNP 2	p52919-chr12_125296601	Intron 4	T, 0.0013	GG/GT	734/2	-7.4063	0.0043		741/2	-13.4137	0.0385	
59	SNP 3	p52956-rs77740046	Intron 4	T, 0.0546	CC/CT/TT	651/75/3	0.3300	0.4288		657/75/3	-0.4428	0.6689	
59	SNP 4	p52995-rs113910315	Intron 4-splice site	G, 0.002	TG/TT	3/740	-1.4175	0.5038		3/746	0.6563	0.9010	
60	SNP 1	p52919-chr12_125296601	Intron 4	T, 0.0013	GG/GT	734/2	-7.4063	0.0043	0.1743	741/2	-13.4137	0.0385	0.5102
60	SNP 2	p52956-rs77740046	Intron 4	T, 0.0546	CC/CT/TT	651/75/3	0.3300	0.4288		657/75/3	-0.4428	0.6689	
60	SNP 3	p52995-rs113910315	Intron 4-splice site	G, 0.002	TG/TT	3/740	-1.4175	0.5038		3/746	0.6563	0.9010	
60	SNP 4	p53359-rs112371713	Intron 5	A, 0.1243	AA/GA/GG	9/160/549	0.4193	0.1651		10/161/554	0.8266	0.2605	
61	SNP 1	p52956-rs77740046	Intron 4	T, 0.0546	CC/CT/TT	651/75/3	0.3300	0.4288	0.1406	657/75/3	-0.4428	0.6689	0.2847
61	SNP 2	p52995-rs113910315	Intron 4-splice site	G, 0.002	TG/TT	3/740	-1.4175	0.5038		3/746	0.6563	0.9010	
61	SNP 3	p53359-rs112371713	Intron 5	A, 0.1243	AA/GA/GG	9/160/549	0.4193	0.1651		10/161/554	0.8266	0.2605	
61	SNP 4	p53372-rs115604379	Intron 5	T, 0.0066	CC/CT	729/10	3.0372	0.0093		735/10	4.3130	0.1386	
62	SNP 1	p52995-rs113910315	Intron 4-splice site	G, 0.002	TG/TT	3/740	-1.4175	0.5038	0.0712	3/746	0.6563	0.9010	0.3287
62	SNP 2	p53359-rs112371713	Intron 5	A, 0.1243	AA/GA/GG	9/160/549	0.4193	0.1651		10/161/554	0.8266	0.2605	
62	SNP 3	p53372-rs115604379	Intron 5	T, 0.0066	CC/CT	729/10	3.0372	0.0093		735/10	4.3130	0.1386	
62	SNP 4	p53790-rs4765614	Intron 5	A, 0.2653	GG/GA/AA	399/276/58	-0.3281	0.1218		402/277/59	-0.4621	0.3775	
63	SNP 1	p53359-rs112371713	Intron 5	A, 0.1243	AA/GA/GG	9/160/549	0.4193	0.1651	**0.0394**	10/161/554	0.8266	0.2605	0.3391
63	SNP 2	p53372-rs115604379	Intron 5	T, 0.0066	CC/CT	729/10	3.0372	0.0093		735/10	4.3130	0.1386	
63	SNP 3	p53790-rs4765614	Intron 5	A, 0.2653	GG/GA/AA	399/276/58	-0.3281	0.1218		402/277/59	-0.4621	0.3775	
63	SNP 4	p54445-rs60910935	Intron 5	G, 0.0418	AA/AG/GG	650/56/2	-0.1247	0.7963		656/57/2	-0.9880	0.4074	
64	SNP 1	p53372-rs115604379	Intron 5	T, 0.0066	CC/CT	729/10	3.0372	0.0093	0.0691	735/10	4.3130	0.1386	0.5878
64	SNP 2	p53790-rs4765614	Intron 5	A, 0.2653	GG/GA/AA	399/276/58	-0.3281	0.1218		402/277/59	-0.4621	0.3775	
64	SNP 3	p54445-rs60910935	Intron 5	G, 0.0418	AA/AG/GG	650/56/2	-0.1247	0.7963		656/57/2	-0.9880	0.4074	
64	SNP 4	p54475-rs60227139	Intron 5	T, 0.0437	CC/CT/TT	670/61/2	-0.0602	0.8966		676/61/2	-0.7550	0.5114	
65	SNP 1	p53790-rs4765614	Intron 5	A, 0.2653	GG/GA/AA	399/276/58	-0.3281	0.1218	0.4454	402/277/59	-0.4621	0.3775	0.6493
65	SNP 2	p54445-rs60910935	Intron 5	G, 0.0418	AA/AG/GG	650/56/2	-0.1247	0.7963		656/57/2	-0.9880	0.4074	
65	SNP 3	p54475-rs60227139	Intron 5	T, 0.0437	CC/CT/TT	670/61/2	-0.0602	0.8966		676/61/2	-0.7550	0.5114	
65	SNP 4	p54492-rs61762481	Intron 5	A, 0.1005	AA/GA/GG	8/137/607	-0.1522	0.6257		8/137/618	-0.1546	0.8416	
66	SNP 1	p54445-rs60910935	Intron 5	G, 0.0418	AA/AG/GG	650/56/2	-0.1247	0.7963	0.4715	656/57/2	-0.9880	0.4074	0.3202
66	SNP 2	p54475-rs60227139	Intron 5	T, 0.0437	CC/CT/TT	670/61/2	-0.0602	0.8966		676/61/2	-0.7550	0.5114	
66	SNP 3	p54492-rs61762481	Intron 5	A, 0.1005	AA/GA/GG	8/137/607	-0.1522	0.6257		8/137/618	-0.1546	0.8416	
66	SNP 4	p54611-chr12_125294909	Intron 5	C, 0.0007	TC/TT	1/742	-9.5243	0.0097		1/748	-19.2831	0.0344	
67	SNP 1	p54475-rs60227139	Intron 5	T, 0.0437	CC/CT/TT	670/61/2	-0.0602	0.8966	0.8784	676/61/2	-0.7550	0.5114	0.8769
67	SNP 2	p54492-rs61762481	Intron 5	A, 0.1005	AA/GA/GG	8/137/607	-0.1522	0.6257		8/137/618	-0.1546	0.8416	
67	SNP 3	p54611-chr12_125294909	Intron 5	C, 0.0007	TC/TT	1/742	-9.5243	0.0097		1/748	-19.2831	0.0344	
67	SNP 4	p54627-chr12_125294893	Intron 5	C, 0.002	GC/GG	3/733	0.9473	0.6571		3/744	3.6910	0.4850	
68	SNP 1	p54492-rs61762481	Intron 5	A, 0.1005	AA/GA/GG	8/137/607	-0.1522	0.6257	0.1570	8/137/618	-0.1546	0.8416	0.2715
68	SNP 2	p54611-chr12_125294909	Intron 5	C, 0.0007	TC/TT	1/742	-9.5243	0.0097		1/748	-19.2831	0.0344	
68	SNP 3	p54627-chr12_125294893	Intron 5	C, 0.002	GC/GG	3/733	0.9473	0.6571		3/744	3.6910	0.4850	
68	SNP 4	p54856-chr12_125294664	Intron 6	T, 0.0007	CC/CT	742/1	-8.4305	0.0215		748/1	-24.0757	0.0082	
69	SNP 1	p54611-chr12_125294909	Intron 5	C, 0.0007	TC/TT	1/742	-9.5243	0.0097	0.0610	1/748	-19.2831	0.0344	0.2063
69	SNP 2	p54627-chr12_125294893	Intron 5	C, 0.002	GC/GG	3/733	0.9473	0.6571		3/744	3.6910	0.4850	
69	SNP 3	p54856-chr12_125294664	Intron 6	T, 0.0007	CC/CT	742/1	-8.4305	0.0215		748/1	-24.0757	0.0082	
69	SNP 4	p55923-rs838900	Intron 6	A, 0.3921	AA/GA/GG	113/345/275	0.2787	0.1549		115/346/282	0.3606	0.4549	
70	SNP 1	p54627-chr12_125294893	Intron 5	C, 0.002	GC/GG	3/733	0.9473	0.6571	0.0599	3/744	3.6910	0.4850	**0.0140**
70	SNP 2	p54856-chr12_125294664	Intron 6	T, 0.0007	CC/CT	742/1	-8.4305	0.0215		748/1	-24.0757	0.0082	
70	SNP 3	p55923-rs838900	Intron 6	A, 0.3921	AA/GA/GG	113/345/275	0.2787	0.1549		115/346/282	0.3606	0.4549	
70	SNP 4	p55963-rs7134858	Intron 6	T, 0.156	TT/CT/CC	24/184/532	0.4418	0.0799		24/190/537	1.7537	0.0052	
71	SNP 1	p54856-chr12_125294664	Intron 6	T, 0.0007	CC/CT	742/1	-8.4305	0.0215	0.0995	748/1	-24.0757	0.0082	**0.0488**
71	SNP 2	p55923-rs838900	Intron 6	A, 0.3921	AA/GA/GG	113/345/275	0.2787	0.1549		115/346/282	0.3606	0.4549	
71	SNP 3	p55963-rs7134858	Intron 6	T, 0.156	TT/CT/CC	24/184/532	0.4418	0.0799		24/190/537	1.7537	0.0052	
71	SNP 4	p56845-rs838902	Intron 6	G, 0.4249	AA/AG/GG	249/347/141	-0.0786	0.6801		256/350/143	-0.3052	0.5129	
72	SNP 1	p55923-rs838900	Intron 6	A, 0.3921	AA/GA/GG	113/345/275	0.2787	0.1549	**0.0315**	115/346/282	0.3606	0.4549	**0.0463**
72	SNP 2	p55963-rs7134858	Intron 6	T, 0.156	TT/CT/CC	24/184/532	0.4418	0.0799		24/190/537	1.7537	0.0052	
72	SNP 3	p56845-rs838902	Intron 6	G, 0.4249	AA/AG/GG	249/347/141	-0.0786	0.6801		256/350/143	-0.3052	0.5129	
72	SNP 4	p57004-rs187562853	Intron 6	A, 0.0098	GG/GA	721/15	1.6474	0.0872		731/15	3.2853	0.1690	

73	SNP 1	p55963-rs7134858	Intron 6	T, 0.156	TT/CT/CC	24/184/532	0.4418	0.0799	0.2741	24/190/537	1.7537	0.0052	0.0553
73	SNP 2	p56845-rs838902	Intron 6	G, 0.4249	AA/AG/GG	249/347/141	-0.0786	0.6801		256/350/143	-0.3052	0.5129	
73	SNP 3	p57004-rs187562853	Intron 6	A, 0.0098	GG/GA	721/15	1.6474	0.0872		731/15	3.2853	0.1690	
73	SNP 4	p57107-rs5892	Exon 7	T, 0.0589	CC/CT/TT	656/85/1	0.0079	0.9848		663/84/1	-0.4697	0.6488	
74	SNP 1	p56845-rs838902	Intron 6	G, 0.4249	AA/AG/GG	249/347/141	-0.0786	0.6801	0.4787	256/350/143	-0.3052	0.5129	0.5963
74	SNP 2	p57004-rs187562853	Intron 6	A, 0.0098	GG/GA	721/15	1.6474	0.0872		731/15	3.2853	0.1690	
74	SNP 3	p57107-rs5892	Exon 7	T, 0.0589	CC/CT/TT	656/85/1	0.0079	0.9848		663/84/1	-0.4697	0.6488	
74	SNP 4	p57508-rs71458866	Intron 7	A, 0.113	AA/GA/GG	13/144/594	-0.1926	0.5089		12/147/602	-0.6325	0.3861	
75	SNP 1	p57004-rs187562853	Intron 6	A, 0.0098	GG/GA	721/15	1.6474	0.0872	0.5892	731/15	3.2853	0.1690	0.2923
75	SNP 2	p57107-rs5892	Exon 7	T, 0.0589	CC/CT/TT	656/85/1	0.0079	0.9848		663/84/1	-0.4697	0.6488	
75	SNP 3	p57508-rs71458866	Intron 7	A, 0.113	AA/GA/GG	13/144/594	-0.1926	0.5089		12/147/602	-0.6325	0.3861	
75	SNP 4	p57592-rs838903	Intron 7	A, 0.3763	AA/GA/GG	111/334/290	-0.1598	0.4149		113/336/297	-0.7661	0.1109	
76	SNP 1	p57107-rs5892	Exon 7	T, 0.0589	CC/CT/TT	656/85/1	0.0079	0.9848	0.9374	663/84/1	-0.4697	0.6488	0.5355
76	SNP 2	p57508-rs71458866	Intron 7	A, 0.113	AA/GA/GG	13/144/594	-0.1926	0.5089		12/147/602	-0.6325	0.3861	
76	SNP 3	p57592-rs838903	Intron 7	A, 0.3763	AA/GA/GG	111/334/290	-0.1598	0.4149		113/336/297	-0.7661	0.1109	
76	SNP 4	p58514-rs838905	Intron 7	C, 0.4329	CC/TC/TT	146/350/243	-0.0855	0.6536		149/352/250	-0.4213	0.3646	
77	SNP 1	p57508-rs71458866	Intron 7	A, 0.113	AA/GA/GG	13/144/594	-0.1926	0.5089	0.8016	12/147/602	-0.6325	0.3861	0.5014
77	SNP 2	p57592-rs838903	Intron 7	A, 0.3763	AA/GA/GG	111/334/290	-0.1598	0.4149		113/336/297	-0.7661	0.1109	
77	SNP 3	p58514-rs838905	Intron 7	C, 0.4329	CC/TC/TT	146/350/243	-0.0855	0.6536		149/352/250	-0.4213	0.3646	
77	SNP 4	p58664-rs865716	Intron 7	T, 0.2708	AA/AT/TT	395/268/62	-0.0965	0.6449		395/272/63	0.5369	0.3008	
78	SNP 1	p57592-rs838903	Intron 7	A, 0.3763	AA/GA/GG	111/334/290	-0.1598	0.4149	0.1155	113/336/297	-0.7661	0.1109	**0.0326**
78	SNP 2	p58514-rs838905	Intron 7	C, 0.4329	CC/TC/TT	146/350/243	-0.0855	0.6536		149/352/250	-0.4213	0.3646	
78	SNP 3	p58664-rs865716	Intron 7	T, 0.2708	AA/AT/TT	395/268/62	-0.0965	0.6449		395/272/63	0.5369	0.3008	
78	SNP 4	p60255-rs3782287	Intron 7	T, 0.2831	CC/CT/TT	378/312/51	0.2555	0.2453		384/314/55	0.3715	0.4856	
79	SNP 1	p58514-rs838905	Intron 7	C, 0.4329	CC/TC/TT	146/350/243	-0.0855	0.6536	0.0820	149/352/250	-0.4213	0.3646	**0.0256**
79	SNP 2	p58664-rs865716	Intron 7	T, 0.2708	AA/AT/TT	395/268/62	-0.0965	0.6449		395/272/63	0.5369	0.3008	
79	SNP 3	p60255-rs3782287	Intron 7	T, 0.2831	CC/CT/TT	378/312/51	0.2555	0.2453		384/314/55	0.3715	0.4856	
79	SNP 4	p61872-rs838909	Intron 7	T, 0.2199	CC/CT/TT	449/249/36	0.1223	0.5945		453/251/36	0.9232	0.1056	
80	SNP 1	p58664-rs865716	Intron 7	T, 0.2708	AA/AT/TT	395/268/62	-0.0965	0.6449	0.1146	395/272/63	0.5369	0.3008	**0.0030**
80	SNP 2	p60255-rs3782287	Intron 7	T, 0.2831	CC/CT/TT	378/312/51	0.2555	0.2453		384/314/55	0.3715	0.4856	
80	SNP 3	p61872-rs838909	Intron 7	T, 0.2199	CC/CT/TT	449/249/36	0.1223	0.5945		453/251/36	0.9232	0.1056	
80	SNP 4	p62140-rs838910	Intron 7	T, 0.3047	GG/GT/TT	355/304/70	-0.0759	0.7143		358/304/72	-0.0755	0.8821	
81	SNP 1	p60255-rs3782287	Intron 7	T, 0.2831	CC/CT/TT	378/312/51	0.2555	0.2453	0.2078	384/314/55	0.3715	0.4856	**0.0050**
81	SNP 2	p61872-rs838909	Intron 7	T, 0.2199	CC/CT/TT	449/249/36	0.1223	0.5945		453/251/36	0.9232	0.1056	
81	SNP 3	p62140-rs838910	Intron 7	T, 0.3047	GG/GT/TT	355/304/70	-0.0759	0.7143		358/304/72	-0.0755	0.8821	
81	SNP 4	p62409-rs838911	Intron 7	T, 0.4211	CC/CT/TT	247/347/133	-0.1556	0.4213		251/346/135	-0.6245	0.1888	
82	SNP 1	p61872-rs838909	Intron 7	T, 0.2199	CC/CT/TT	449/249/36	0.1223	0.5945	0.1998	453/251/36	0.9232	0.1056	**0.0137**
82	SNP 2	p62140-rs838910	Intron 7	T, 0.3047	GG/GT/TT	355/304/70	-0.0759	0.7143		358/304/72	-0.0755	0.8821	
82	SNP 3	p62409-rs838911	Intron 7	T, 0.4211	CC/CT/TT	247/347/133	-0.1556	0.4213		251/346/135	-0.6245	0.1888	
82	SNP 4	p62615-rs7138386	Intron 7	C, 0.1137	TT/TC/CC	568/138/12	-0.2083	0.4851		571/141/11	-0.6495	0.3851	
83	SNP 1	p62140-rs838910	Intron 7	T, 0.3047	GG/GT/TT	355/304/70	-0.0759	0.7143	0.2052	358/304/72	-0.0755	0.8821	**0.0187**
83	SNP 2	p62409-rs838911	Intron 7	T, 0.4211	CC/CT/TT	247/347/133	-0.1556	0.4213		251/346/135	-0.6245	0.1888	
83	SNP 3	p62615-rs7138386	Intron 7	C, 0.1137	TT/TC/CC	568/138/12	-0.2083	0.4851		571/141/11	-0.6495	0.3851	
83	SNP 4	p63483-rs838912	Intron 7	A, 0.0867	AA/GA/GG	6/117/615	0.1814	0.5840		6/119/624	1.8700	0.0234	
84	SNP 1	p62409-rs838911	Intron 7	T, 0.4211	CC/CT/TT	247/347/133	-0.1556	0.4213	0.7674	251/346/135	-0.6245	0.1888	0.1544
84	SNP 2	p62615-rs7138386	Intron 7	C, 0.1137	TT/TC/CC	568/138/12	-0.2083	0.4851		571/141/11	-0.6495	0.3851	
84	SNP 3	p63483-rs838912	Intron 7	A, 0.0867	AA/GA/GG	6/117/615	0.1814	0.5840		6/119/624	1.8700	0.0234	
84	SNP 4	p64772-rs5888	Exon 8	T, 0.0961	CC/CT/TT	605/129/8	0.3620	0.2561		614/130/8	2.0962	0.0080	
85	SNP 1	p62615-rs7138386	Intron 7	C, 0.1137	TT/TC/CC	568/138/12	-0.2083	0.4851	0.7618	571/141/11	-0.6495	0.3851	0.1130
85	SNP 2	p63483-rs838912	Intron 7	A, 0.0867	AA/GA/GG	6/117/615	0.1814	0.5840		6/119/624	1.8700	0.0234	
85	SNP 3	p64772-rs5888	Exon 8	T, 0.0961	CC/CT/TT	605/129/8	0.3620	0.2561		614/130/8	2.0962	0.0080	
85	SNP 4	p64923-rs838915	Intron 8	A, 0.1435	AA/CA/CC	19/177/539	-0.0858	0.7466		19/179/547	-0.3684	0.5766	
86	SNP 1	p63483-rs838912	Intron 7	A, 0.0867	AA/GA/GG	6/117/615	0.1814	0.5840	0.1859	6/119/624	1.8700	0.0234	**0.0290**
86	SNP 2	p64772-rs5888	Exon 8	T, 0.0961	CC/CT/TT	605/129/8	0.3620	0.2561		614/130/8	2.0962	0.0080	
86	SNP 3	p64923-rs838915	Intron 8	A, 0.1435	AA/CA/CC	19/177/539	-0.0858	0.7466		19/179/547	-0.3684	0.5766	
86	SNP 4	p65999-rs12819677	Intron 8	A, 0.2813	GG/GA/AA	371/310/50	0.4021	0.0670		375/309/52	0.6769	0.2052	
87	SNP 1	p64772-rs5888	Exon 8	T, 0.0961	CC/CT/TT	605/129/8	0.3620	0.2561	0.1207	614/130/8	2.0962	0.0080	0.0623
87	SNP 2	p64923-rs838915	Intron 8	A, 0.1435	AA/CA/CC	19/177/539	-0.0858	0.7466		19/179/547	-0.3684	0.5766	
87	SNP 3	p65999-rs12819677	Intron 8	A, 0.2813	GG/GA/AA	371/310/50	0.4021	0.0670		375/309/52	0.6769	0.2052	
87	SNP 4	p67439-rs961170	Intron 8	A, 0.0893	AA/GA/GG	11/108/610	-0.0481	0.8812		11/108/615	-0.9808	0.2211	
88	SNP 1	p64923-rs838915	Intron 8	A, 0.1435	AA/CA/CC	19/177/539	-0.0858	0.7466	0.2674	19/179/547	-0.3684	0.5766	0.1964
88	SNP 2	p65999-rs12819677	Intron 8	A, 0.2813	GG/GA/AA	371/310/50	0.4021	0.0670		375/309/52	0.6769	0.2052	

88	SNP 3	p67439-rs961170	Intron 8	A, 0.0893	AA/GA/GG	11/108/610	-0.0481	0.8812		11/108/615	-0.9808	0.2211	
88	SNP 4	p67700-rs1726374	Intron 8	A, 0.1933	GG/GA/AA	491/221/35	0.2289	0.3262		500/226/32	0.3249	0.5798	
89	SNP 1	p65999-rs12819677	Intron 8	A, 0.2813	GG/GA/AA	371/310/50	0.4021	0.0670	0.2789	375/309/52	0.6769	0.2052	0.1543
89	SNP 2	p67439-rs961170	Intron 8	A, 0.0893	AA/GA/GG	11/108/610	-0.0481	0.8812		11/108/615	-0.9808	0.2211	
89	SNP 3	p67700-rs1726374	Intron 8	A, 0.1933	GG/GA/AA	491/221/35	0.2289	0.3262		500/226/32	0.3249	0.5798	
89	SNP 4	p69013-rs7135117	Intron 8	G, 0.2901	GG/AG/AA	75/275/377	0.1133	0.5758		74/275/383	0.5010	0.3196	
90	SNP 1	p67439-rs961170	Intron 8	A, 0.0893	AA/GA/GG	11/108/610	-0.0481	0.8812	0.4209	11/108/615	-0.9808	0.2211	0.1317
90	SNP 2	p67700-rs1726374	Intron 8	A, 0.1933	GG/GA/AA	491/221/35	0.2289	0.3262		500/226/32	0.3249	0.5798	
90	SNP 3	p69013-rs7135117	Intron 8	G, 0.2901	GG/AG/AA	75/275/377	0.1133	0.5758		74/275/383	0.5010	0.3196	
90	SNP 4	p69699-rs10396210	Intron 8-splice site	A, 0.1511	AA/GA/GG	17/186/523	-0.4778	0.0745		17/188/527	-1.2921	0.0520	
91	SNP 1	p67700-rs1726374	Intron 8	A, 0.1933	GG/GA/AA	491/221/35	0.2289	0.3262	0.4715	500/226/32	0.3249	0.5798	0.4607
91	SNP 2	p69013-rs7135117	Intron 8	G, 0.2901	GG/AG/AA	75/275/377	0.1133	0.5758		74/275/383	0.5010	0.3196	
91	SNP 3	p69699-rs10396210	Intron 8-splice site	A, 0.1511	AA/GA/GG	17/186/523	-0.4778	0.0745		17/188/527	-1.2921	0.0520	
91	SNP 4	p69995delC-rs5801571	Intron 9	delC, 0.2761	DD/WD/WW	63/281/386	0.0614	0.7724		63/282/394	0.2920	0.5768	
92	SNP 1	p69013-rs7135117	Intron 8	G, 0.2901	GG/AG/AA	75/275/377	0.1133	0.5758	0.2848	74/275/383	0.5010	0.3196	0.4449
92	SNP 2	p69699-rs10396210	Intron 8-splice site	A, 0.1511	AA/GA/GG	17/186/523	-0.4778	0.0745		17/188/527	-1.2921	0.0520	
92	SNP 3	p69995delC-rs5801571	Intron 9	delC, 0.2761	DD/WD/WW	63/281/386	0.0614	0.7724		63/282/394	0.2920	0.5768	
92	SNP 4	p71867-rs7954022	Intron 9	T, 0.1323	TT/CT/CC	11/172/552	0.3876	0.1764		12/172/556	0.8502	0.2241	
93	SNP 1	p69699-rs10396210	Intron 8-splice site	A, 0.1511	AA/GA/GG	17/186/523	-0.4778	0.0745	0.6336	17/188/527	-1.2921	0.0520	0.2477
93	SNP 2	p69995delC-rs5801571	Intron 9	delC, 0.2761	DD/WD/WW	63/281/386	0.0614	0.7724		63/282/394	0.2920	0.5768	
93	SNP 3	p71867-rs7954022	Intron 9	T, 0.1323	TT/CT/CC	11/172/552	0.3876	0.1764		12/172/556	0.8502	0.2241	
93	SNP 4	p72197-rs838861	Intron 9	G, 0.3777	AA/AG/GG	300/308/123	-0.1415	0.4527		304/310/122	-0.1507	0.7464	
94	SNP 1	p69995delC-rs5801571	Intron 9	delC, 0.2761	DD/WD/WW	63/281/386	0.0614	0.7724	0.7410	63/282/394	0.2920	0.5768	0.3380
94	SNP 2	p71867-rs7954022	Intron 9	T, 0.1323	TT/CT/CC	11/172/552	0.3876	0.1764		12/172/556	0.8502	0.2241	
94	SNP 3	p72197-rs838861	Intron 9	G, 0.3777	AA/AG/GG	300/308/123	-0.1415	0.4527		304/310/122	-0.1507	0.7464	
94	SNP 4	p72777-rs838862	Intron 9	T, 0.0887	CC/CT/TT	607/115/7	0.1013	0.7613		611/116/7	0.7012	0.3938	
95	SNP 1	p71867-rs7954022	Intron 9	T, 0.1323	TT/CT/CC	11/172/552	0.3876	0.1764	0.1054	12/172/556	0.8502	0.2241	**0.0131**
95	SNP 2	p72197-rs838861	Intron 9	G, 0.3777	AA/AG/GG	300/308/123	-0.1415	0.4527		304/310/122	-0.1507	0.7464	
95	SNP 3	p72777-rs838862	Intron 9	T, 0.0887	CC/CT/TT	607/115/7	0.1013	0.7613		611/116/7	0.7012	0.3938	
95	SNP 4	p75766-rs838866	Intron 9	C, 0.2116	TT/TC/CC	457/239/38	-0.1058	0.6423		466/240/37	-0.0497	0.9306	
96	SNP 1	p72197-rs838861	Intron 9	G, 0.3777	AA/AG/GG	300/308/123	-0.1415	0.4527	0.4137	304/310/122	-0.1507	0.7464	**0.0484**
96	SNP 2	p72777-rs838862	Intron 9	T, 0.0887	CC/CT/TT	607/115/7	0.1013	0.7613		611/116/7	0.7012	0.3938	
96	SNP 3	p75766-rs838866	Intron 9	C, 0.2116	TT/TC/CC	457/239/38	-0.1058	0.6423		466/240/37	-0.0497	0.9306	
96	SNP 4	p75778-rs7301120	Intron 9	T, 0.1135	TT/CT/CC	9/147/563	-0.2367	0.4366		9/146/569	0.3767	0.6174	
97	SNP 1	p72777-rs838862	Intron 9	T, 0.0887	CC/CT/TT	607/115/7	0.1013	0.7613	0.0867	611/116/7	0.7012	0.3938	**0.0098**
97	SNP 2	p75766-rs838866	Intron 9	C, 0.2116	TT/TC/CC	457/239/38	-0.1058	0.6423		466/240/37	-0.0497	0.9306	
97	SNP 3	p75778-rs7301120	Intron 9	T, 0.1135	TT/CT/CC	9/147/563	-0.2367	0.4366		9/146/569	0.3767	0.6174	
97	SNP 4	p76757-rs9919713	Intron 9	T, 0.439	AA/AT/TT	235/347/151	-0.1264	0.5044		243/353/148	-0.1860	0.6921	
98	SNP 1	p75766-rs838866	Intron 9	C, 0.2116	TT/TC/CC	457/239/38	-0.1058	0.6423	0.5187	466/240/37	-0.0497	0.9306	0.7976
98	SNP 2	p75778-rs7301120	Intron 9	T, 0.1135	TT/CT/CC	9/147/563	-0.2367	0.4366		9/146/569	0.3767	0.6174	
98	SNP 3	p76757-rs9919713	Intron 9	T, 0.439	AA/AT/TT	235/347/151	-0.1264	0.5044		243/353/148	-0.1860	0.6921	
98	SNP 4	p77181-rs146246031	Intron 9	C, 0.0053	TC/TT	6/731	-0.6173	0.6809		8/734	0.4564	0.8874	
99	SNP 1	p75778-rs7301120	Intron 9	T, 0.1135	TT/CT/CC	9/147/563	-0.2367	0.4366	0.7604	9/146/569	0.3767	0.6174	0.5753
99	SNP 2	p76757-rs9919713	Intron 9	T, 0.439	AA/AT/TT	235/347/151	-0.1264	0.5044		243/353/148	-0.1860	0.6921	
99	SNP 3	p77181-rs146246031	Intron 9	C, 0.0053	TC/TT	6/731	-0.6173	0.6809		8/734	0.4564	0.8874	
99	SNP 4	p77251-rs34339961	Intron 9	T, 0.1177	AA/AT/TT	561/151/11	-0.1372	0.6426		573/150/11	0.7120	0.3383	
100	SNP 1	p76757-rs9919713	Intron 9	T, 0.439	AA/AT/TT	235/347/151	-0.1264	0.5044	0.8818	243/353/148	-0.1860	0.6921	0.6540
100	SNP 2	p77181-rs146246031	Intron 9	C, 0.0053	TC/TT	6/731	-0.6173	0.6809		8/734	0.4564	0.8874	
100	SNP 3	p77251-rs34339961	Intron 9	T, 0.1177	AA/AT/TT	561/151/11	-0.1372	0.6426		573/150/11	0.7120	0.3383	
100	SNP 4	p77381-rs138499966	Intron 9	C, 0.0046	TC/TT	7/735	1.5523	0.2652		7/741	-0.2236	0.9485	
101	SNP 1	p77181-rs146246031	Intron 9	C, 0.0053	TC/TT	6/731	-0.6173	0.6809	0.7886	8/734	0.4564	0.8874	0.6293
101	SNP 2	p77251-rs34339961	Intron 9	T, 0.1177	AA/AT/TT	561/151/11	-0.1372	0.6426		573/150/11	0.7120	0.3383	
101	SNP 3	p77381-rs138499966	Intron 9	C, 0.0046	TC/TT	7/735	1.5523	0.2652		7/741	-0.2236	0.9485	
101	SNP 4	p77620-rs377124254	Intron 10	A, 0.0007	GA/GG	1/735	11.5518	0.0016		1/741	14.4685	0.1141	
102	SNP 1	p77251-rs34339961	Intron 9	T, 0.1177	AA/AT/TT	561/151/11	-0.1372	0.6426	0.5420	573/150/11	0.7120	0.3383	0.4369
102	SNP 2	p77381-rs138499966	Intron 9	C, 0.0046	TC/TT	7/735	1.5523	0.2652		7/741	-0.2236	0.9485	
102	SNP 3	p77620-rs377124254	Intron 10	A, 0.0007	GA/GG	1/735	11.5518	0.0016		1/741	14.4685	0.1141	

102	SNP 4	p77682-rs150082885	Intron 10	G, 0.0106	AA/AG/GG	716/13/1	-0.5377	0.5509		721/14/1	-2.9877	0.1698	
103	SNP 1	p77381-rs138499966	Intron 9	C, 0.0046	TC/TT	7/735	1.5523	0.2652	0.8039	7/741	-0.2236	0.9485	0.3187
103	SNP 2	p77620-rs377124254	Intron 10	A, 0.0007	GA/GG	1/735	11.5518	0.0016		1/741	14.4685	0.1141	
103	SNP 3	p77682-rs150082885	Intron 10	G, 0.0106	AA/AG/GG	716/13/1	-0.5377	0.5509		721/14/1	-2.9877	0.1698	
103	SNP 4	p77704-chr12_125271816	Intron 10	A, 0.004	CA/CC	5/726	-1.4602	0.3791		6/731	-3.0744	0.4152	
104	SNP 1	p77620-rs377124254	Intron 10	A, 0.0007	GA/GG	1/735	11.5518	0.0016	0.7533	1/741	14.4685	0.1141	0.3571
104	SNP 2	p77682-rs150082885	Intron 10	G, 0.0106	AA/AG/GG	716/13/1	-0.5377	0.5509		721/14/1	-2.9877	0.1698	
104	SNP 3	p77704-chr12_125271816	Intron 10	A, 0.004	CA/CC	5/726	-1.4602	0.3791		6/731	-3.0744	0.4152	
104	SNP 4	p77842-rs2272310	Intron 10	A, 0.0807	AA/GA/GG	5/110/630	0.2011	0.5651		5/110/642	0.8339	0.3359	
105	SNP 1	p77682-rs150082885	Intron 10	G, 0.0106	AA/AG/GG	716/13/1	-0.5377	0.5509	0.8340	721/14/1	-2.9877	0.1698	0.4218
105	SNP 2	p77704-chr12_125271816	Intron 10	A, 0.004	CA/CC	5/726	-1.4602	0.3791		6/731	-3.0744	0.4152	
105	SNP 3	p77842-rs2272310	Intron 10	A, 0.0807	AA/GA/GG	5/110/630	0.2011	0.5651		5/110/642	0.8339	0.3359	
105	SNP 4	p78255-rs184052375	Intron 10	G, 0.0072	AA/AG	732/11	1.1808	0.2893		740/9	3.3097	0.2790	
106	SNP 1	p77704-chr12_125271816	Intron 10	A, 0.004	CA/CC	5/726	-1.4602	0.3791	0.9158	6/731	-3.0744	0.4152	0.3479
106	SNP 2	p77842-rs2272310	Intron 10	A, 0.0807	AA/GA/GG	5/110/630	0.2011	0.5651		5/110/642	0.8339	0.3359	
106	SNP 3	p78255-rs184052375	Intron 10	G, 0.0072	AA/AG	732/11	1.1808	0.2893		740/9	3.3097	0.2790	
106	SNP 4	p78402-rs838898	Intron 10	A, 0.0714	AA/GA/GG	7/86/594	-0.0602	0.8720		7/86/601	-0.9806	0.2889	
107	SNP 1	p77842-rs2272310	Intron 10	A, 0.0807	AA/GA/GG	5/110/630	0.2011	0.5651	0.8730	5/110/642	0.8339	0.3359	0.5898
107	SNP 2	p78255-rs184052375	Intron 10	G, 0.0072	AA/AG	732/11	1.1808	0.2893		740/9	3.3097	0.2790	
107	SNP 3	p78402-rs838898	Intron 10	A, 0.0714	AA/GA/GG	7/86/594	-0.0602	0.8720		7/86/601	-0.9806	0.2889	
107	SNP 4	p78430-rs838897	Intron 10	G, 0.383	GG/CG/CC	125/308/291	0.0070	0.9704		121/315/299	-0.1887	0.6887	
108	SNP 1	p78255-rs184052375	Intron 10	G, 0.0072	AA/AG	732/11	1.1808	0.2893	0.4248	740/9	3.3097	0.2790	0.0975
108	SNP 2	p78402-rs838898	Intron 10	A, 0.0714	AA/GA/GG	7/86/594	-0.0602	0.8720		7/86/601	-0.9806	0.2889	
108	SNP 3	p78430-rs838897	Intron 10	G, 0.383	GG/CG/CC	125/308/291	0.0070	0.9704		121/315/299	-0.1887	0.6887	
108	SNP 4	p78747-rs2293440	Intron 11	C, 0.4112	CC/TC/TT	128/342/252	-0.1684	0.3806		126/348/258	-0.2984	0.5352	
109	SNP 1	p78402-rs838898	Intron 10	A, 0.0714	AA/GA/GG	7/86/594	-0.0602	0.8720	0.4162	7/86/601	-0.9806	0.2889	**0.0195**
109	SNP 2	p78430-rs838897	Intron 10	G, 0.383	GG/CG/CC	125/308/291	0.0070	0.9704		121/315/299	-0.1887	0.6887	
109	SNP 3	p78747-rs2293440	Intron 11	C, 0.4112	CC/TC/TT	128/342/252	-0.1684	0.3806		126/348/258	-0.2984	0.5352	
109	SNP 4	p78791-rs75289200	Intron 11	C, 0.0321	TC/TT	46/679	0.7037	0.2078		46/685	3.6568	0.0086	
110	SNP 1	p78430-rs838897	Intron 10	G, 0.383	GG/CG/CC	125/308/291	0.0070	0.9704	0.1013	121/315/299	-0.1887	0.6887	**0.0012**
110	SNP 2	p78747-rs2293440	Intron 11	C, 0.4112	CC/TC/TT	128/342/252	-0.1684	0.3806		126/348/258	-0.2984	0.5352	
110	SNP 3	p78791-rs75289200	Intron 11	C, 0.0321	TC/TT	46/679	0.7037	0.2078		46/685	3.6568	0.0086	
110	SNP 4	p79721-rs838896	Intron 11	C, 0.3104	GG/GC/CC	349/319/73	0.3565	0.0817		357/324/71	1.1147	0.0278	
111	SNP 1	p78747-rs2293440	Intron 11	C, 0.4112	CC/TC/TT	128/342/252	-0.1684	0.3806	**0.0040**	126/348/258	-0.2984	0.5352	**0.0038**
111	SNP 2	p78791-rs75289200	Intron 11	C, 0.0321	TC/TT	46/679	0.7037	0.2078		46/685	3.6568	0.0086	
111	SNP 3	p79721-rs838896	Intron 11	C, 0.3104	GG/GC/CC	349/319/73	0.3565	0.0817		357/324/71	1.1147	0.0278	
111	SNP 4	p79828-rs838895	Intron 11	G, 0.3171	GG/CG/CC	74/322/337	0.4961	0.0162		73/325/345	1.2206	0.0164	
112	SNP 1	p78791-rs75289200	Intron 11	C, 0.0321	TC/TT	46/679	0.7037	0.2078	**0.0055**	46/685	3.6568	0.0086	**0.0412**
112	SNP 2	p79721-rs838896	Intron 11	C, 0.3104	GG/GC/CC	349/319/73	0.3565	0.0817		357/324/71	1.1147	0.0278	
112	SNP 3	p79828-rs838895	Intron 11	G, 0.3171	GG/CG/CC	74/322/337	0.4961	0.0162		73/325/345	1.2206	0.0164	
112	SNP 4	p80045-rs838893	Intron 11	A, 0.3244	GG/GA/AA	335/325/81	0.3127	0.1224		344/328/80	0.8859	0.0774	
113	SNP 1	p79721-rs838896	Intron 11	C, 0.3104	GG/GC/CC	349/319/73	0.3565	0.0817	**0.0048**	357/324/71	1.1147	0.0278	0.1581
113	SNP 2	p79828-rs838895	Intron 11	G, 0.3171	GG/CG/CC	74/322/337	0.4961	0.0162		73/325/345	1.2206	0.0164	
113	SNP 3	p80045-rs838893	Intron 11	A, 0.3244	GG/GA/AA	335/325/81	0.3127	0.1224		344/328/80	0.8859	0.0774	
113	SNP 4	p81863-rs185445624	Intron 11	A, 0.002	GA/GG	3/739	-0.9612	0.6510		3/745	-2.5166	0.6333	
114	SNP 1	p79828-rs838895	Intron 11	G, 0.3171	GG/CG/CC	74/322/337	0.4961	0.0162	**0.0447**	73/325/345	1.2206	0.0164	0.1200
114	SNP 2	p80045-rs838893	Intron 11	A, 0.3244	GG/GA/AA	335/325/81	0.3127	0.1224		344/328/80	0.8859	0.0774	
114	SNP 3	p81863-rs185445624	Intron 11	A, 0.002	GA/GG	3/739	-0.9612	0.6510		3/745	-2.5166	0.6333	
114	SNP 4	p82019-rs838890	Intron 11	T, 0.032	CC/CT/TT	683/42/2	-1.0051	0.0618		690/41/2	-1.1864	0.3782	
115	SNP 1	p80045-rs838893	Intron 11	A, 0.3244	GG/GA/AA	335/325/81	0.3127	0.1224	0.0934	344/328/80	0.8859	0.0774	0.2923
115	SNP 2	p81863-rs185445624	Intron 11	A, 0.002	GA/GG	3/739	-0.9612	0.6510		3/745	-2.5166	0.6333	
115	SNP 3	p82019-rs838890	Intron 11	T, 0.032	CC/CT/TT	683/42/2	-1.0051	0.0618		690/41/2	-1.1864	0.3782	
115	SNP 4	p82264-rs141545424	Exon 12	A, 0.0007	CA/CC	1/739	11.5850	0.0016		1/745	14.4986	0.1119	
116	SNP 1	p81863-rs185445624	Intron 11	A, 0.002	GA/GG	3/739	-0.9612	0.6510	0.0537	3/745	-2.5166	0.6333	0.5297
116	SNP 2	p82019-rs838890	Intron 11	T, 0.032	CC/CT/TT	683/42/2	-1.0051	0.0618		690/41/2	-1.1864	0.3782	
116	SNP 3	p82264-rs141545424	Exon 12	A, 0.0007	CA/CC	1/739	11.5850	0.0016		1/745	14.4986	0.1119	

116	SNP 4	p82340-rs77483223	Intron 12	A, 0.0231	GA/GG	35/699	-1.0458	0.1012		35/705	-1.5601	0.3268	
117	SNP 1	p82019-rs838890	Intron 11	T, 0.032	CC/CT/TT	683/42/2	-1.0051	0.0618	**0.0433**	690/41/2	-1.1864	0.3782	0.3386
117	SNP 2	p82264-rs141545424	Exon 12	A, 0.0007	CA/CC	1/739	11.5850	0.0016		1/745	14.4986	0.1119	
117	SNP 3	p82340-rs77483223	Intron 12	A, 0.0231	GA/GG	35/699	-1.0458	0.1012		35/705	-1.5601	0.3268	
117	SNP 4	p82369-rs75446635	Intron 12	A, 0.0059	GA/GG	9/733	0.5896	0.6322		9/739	2.2239	0.4660	
118	SNP 1	p82264-rs141545424	Exon 12	A, 0.0007	CA/CC	1/739	11.5850	0.0016	**0.0375**	1/745	14.4986	0.1119	0.3863
118	SNP 2	p82340-rs77483223	Intron 12	A, 0.0231	GA/GG	35/699	-1.0458	0.1012		35/705	-1.5601	0.3268	
118	SNP 3	p82369-rs75446635	Intron 12	A, 0.0059	GA/GG	9/733	0.5896	0.6322		9/739	2.2239	0.4660	
118	SNP 4	p82434-rs838889	Intron 12	C, 0.0315	CC/TC/TT	2/42/695	-1.0389	0.0526		2/41/702	-0.9096	0.4967	
119	SNP 1	p82340-rs77483223	Intron 12	A, 0.0231	GA/GG	35/699	-1.0458	0.1012	0.2138	35/705	-1.5601	0.3268	0.7897
119	SNP 2	p82369-rs75446635	Intron 12	A, 0.0059	GA/GG	9/733	0.5896	0.6322		9/739	2.2239	0.4660	
119	SNP 3	p82434-rs838889	Intron 12	C, 0.0315	CC/TC/TT	2/42/695	-1.0389	0.0526		2/41/702	-0.9096	0.4967	
119	SNP 4	p83547-rs838887	Intron 12	G, 0.4564	CC/CG/GG	225/357/154	0.1202	0.5267		227/358/157	-0.1113	0.8119	
120	SNP 1	p82369-rs75446635	Intron 12	A, 0.0059	GA/GG	9/733	0.5896	0.6322	0.3753	9/739	2.2239	0.4660	0.2078
120	SNP 2	p82434-rs838889	Intron 12	C, 0.0315	CC/TC/TT	2/42/695	-1.0389	0.0526		2/41/702	-0.9096	0.4967	
120	SNP 3	p83547-rs838887	Intron 12	G, 0.4564	CC/CG/GG	225/357/154	0.1202	0.5267		227/358/157	-0.1113	0.8119	
120	SNP 4	p83884-rs701106	Intron 12	T, 0.2597	TT/CT/CC	49/289/405	0.2471	0.2601		51/291/412	1.2967	0.0156	
121	SNP 1	p82434-rs838889	Intron 12	C, 0.0315	CC/TC/TT	2/42/695	-1.0389	0.0526	0.2565	2/41/702	-0.9096	0.4967	0.1513
121	SNP 2	p83547-rs838887	Intron 12	G, 0.4564	CC/CG/GG	225/357/154	0.1202	0.5267		227/358/157	-0.1113	0.8119	
121	SNP 3	p83884-rs701106	Intron 12	T, 0.2597	TT/CT/CC	49/289/405	0.2471	0.2601		51/291/412	1.2967	0.0156	
121	SNP 4	p86245-rs188375019	Intron 12	T, 0.0341	CC/CT	690/50	0.7447	0.1639		696/50	1.8399	0.1674	
122	SNP 1	p83547-rs838887	Intron 12	G, 0.4564	CC/CG/GG	225/357/154	0.1202	0.5267	0.3275	227/358/157	-0.1113	0.8119	0.2640
122	SNP 2	p83884-rs701106	Intron 12	T, 0.2597	TT/CT/CC	49/289/405	0.2471	0.2601		51/291/412	1.2967	0.0156	
122	SNP 3	p86245-rs188375019	Intron 12	T, 0.0341	CC/CT	690/50	0.7447	0.1639		696/50	1.8399	0.1674	
122	SNP 4	p86276-rs747155	Intron 12	T, 0.1495	TT/CT/CC	17/187/533	0.2793	0.2980		17/191/541	-0.2164	0.7433	
123	SNP 1	p83884-rs701106	Intron 12	T, 0.2597	TT/CT/CC	49/289/405	0.2471	0.2601	**0.0386**	51/291/412	1.2967	0.0156	**0.0468**
123	SNP 2	p86245-rs188375019	Intron 12	T, 0.0341	CC/CT	690/50	0.7447	0.1639		696/50	1.8399	0.1674	
123	SNP 3	p86276-rs747155	Intron 12	T, 0.1495	TT/CT/CC	17/187/533	0.2793	0.2980		17/191/541	-0.2164	0.7433	
123	SNP 4	p86316-rs701104	Intron 12	T, 0.0487	TT/GT/GG	2/66/643	-0.9838	0.0286		2/64/658	-0.6627	0.5579	
124	SNP 1	p86245-rs188375019	Intron 12	T, 0.0341	CC/CT	690/50	0.7447	0.1639	**0.0368**	696/50	1.8399	0.1674	0.2216
124	SNP 2	p86276-rs747155	Intron 12	T, 0.1495	TT/CT/CC	17/187/533	0.2793	0.2980		17/191/541	-0.2164	0.7433	
124	SNP 3	p86316-rs701104	Intron 12	T, 0.0487	TT/GT/GG	2/66/643	-0.9838	0.0286		2/64/658	-0.6627	0.5579	
124	SNP 4	p86481-rs701103	Exon 13- 3' UTR	A, 0.2451	AA/GA/GG	50/259/424	0.1642	0.4492		50/265/428	-0.0074	0.9891	
125	SNP 1	p86276-rs747155	Intron 12	T, 0.1495	TT/CT/CC	17/187/533	0.2793	0.2980	**0.0307**	17/191/541	-0.2164	0.7433	0.2769
125	SNP 2	p86316-rs701104	Intron 12	T, 0.0487	TT/GT/GG	2/66/643	-0.9838	0.0286		2/64/658	-0.6627	0.5579	
125	SNP 3	p86481-rs701103	Exon 13- 3' UTR	A, 0.2451	AA/GA/GG	50/259/424	0.1642	0.4492		50/265/428	-0.0074	0.9891	
125	SNP 4	p86967-rs187492239	Exon 13- 3' UTR	G, 0.0355	AA/AG	686/52	0.7743	0.1412		692/52	1.3818	0.2924	
126	SNP 1	p86316-rs701104	Intron 12	T, 0.0487	TT/GT/GG	2/66/643	-0.9838	0.0286	0.1487	2/64/658	-0.6627	0.5579	0.1998
126	SNP 2	p86481-rs701103	Exon 13- 3' UTR	A, 0.2451	AA/GA/GG	50/259/424	0.1642	0.4492		50/265/428	-0.0074	0.9891	
126	SNP 3	p86967-rs187492239	Exon 13- 3' UTR	G, 0.0355	AA/AG	686/52	0.7743	0.1412		692/52	1.3818	0.2924	
126	SNP 4	p87011-rs58032386	Exon 13- 3' UTR	T, 0.1417	CC/CT/TT	544/183/14	0.0575	0.8333		551/186/14	-0.2769	0.6829	
127	SNP 1	p86481-rs701103	Exon 13- 3' UTR	A, 0.2451	AA/GA/GG	50/259/424	0.1642	0.4492	0.4750	50/265/428	-0.0074	0.9891	0.8693
127	SNP 2	p86967-rs187492239	Exon 13- 3' UTR	G, 0.0355	AA/AG	686/52	0.7743	0.1412		692/52	1.3818	0.2924	
127	SNP 3	p87011-rs58032386	Exon 13- 3' UTR	T, 0.1417	CC/CT/TT	544/183/14	0.0575	0.8333		551/186/14	-0.2769	0.6829	
127	SNP 4	p87266-rs150512235	Exon 13- 3' UTR[d]	C, 0.0057	TC/TT	9/746	-0.0325	0.9789		9/756	0.0318	0.9917	
128	SNP 1	p86967-rs187492239	Exon 13- 3' UTR	G, 0.0355	AA/AG	686/52	0.7743	0.1412	0.4515	692/52	1.3818	0.2924	0.5899
128	SNP 2	p87011-rs58032386	Exon 13- 3' UTR	T, 0.1417	CC/CT/TT	544/183/14	0.0575	0.8333		551/186/14	-0.2769	0.6829	
128	SNP 3	p87266-rs150512235	Exon 13- 3' UTR[d]	C, 0.0057	TC/TT	9/746	-0.0325	0.9789		9/756	0.0318	0.9917	
128	SNP 4	p87611-rs190688220	3' flanking	T, 0.0316	CC/CT	691/46	0.8329	0.1355		697/46	1.7851	0.1993	
129	SNP 1	p87011-rs58032386	Exon 13- 3' UTR	T, 0.1417	CC/CT/TT	544/183/14	0.0575	0.8333	0.1918	551/186/14	-0.2769	0.6829	0.7888
129	SNP 2	p87266-rs150512235	Exon 13- 3' UTR[d]	C, 0.0057	TC/TT	9/746	-0.0325	0.9789		9/756	0.0318	0.9917	
129	SNP 3	p87611-rs190688220	3' flanking	T, 0.0316	CC/CT	691/46	0.8329	0.1355		697/46	1.7851	0.1993	
129	SNP 4	p87681-rs838883	3' flanking	A, 0.0459	AA/GA/GG	1/65/646	-0.9433	0.0427		1/62/659	-0.0535	0.9639	
130	SNP 1	p87266-rs150512235	Exon 13- 3' UTR[d]	C, 0.0057	TC/TT	9/746	-0.0325	0.9789	0.0787	9/756	0.0318	0.9917	0.5394
130	SNP 2	p87611-rs190688220	3' flanking	T, 0.0316	CC/CT	691/46	0.8329	0.1355		697/46	1.7851	0.1993	

130	SNP 3	p87681-rs838883	3' flanking	A, 0.0459	AA/GA/GG	1/65/646	-0.9433	0.0427		1/62/659	-0.0535	0.9639	
130	SNP 4	p87694-chr12_125261826	3' flanking	T, 0.002	CC/CT	722/3	3.4021	0.1098		731/3	3.0751	0.5620	
131	SNP 1	p87611-rs190688220	3' flanking	T, 0.0316	CC/CT	691/46	0.8329	0.1355	0.0653	697/46	1.7851	0.1993	0.6272
131	SNP 2	p87681-rs838883	3' flanking	A, 0.0459	AA/GA/GG	1/65/646	-0.9433	0.0427		1/62/659	-0.0535	0.9639	
131	SNP 3	p87694-chr12_125261826	3' flanking	T, 0.002	CC/CT	722/3	3.4021	0.1098		731/3	3.0751	0.5620	
131	SNP 4	p87723-rs838881	3' flanking	T, 0.3183	TT/CT/CC	63/341/332	0.1390	0.5146		65/344/338	-0.1897	0.7187	
132	SNP 1	p87681-rs838883	3' flanking	A, 0.0459	AA/GA/GG	1/65/646	-0.9433	0.0427	0.1085	1/62/659	-0.0535	0.9639	0.9672
132	SNP 2	p87694-chr12_125261826	3' flanking	T, 0.002	CC/CT	722/3	3.4021	0.1098		731/3	3.0751	0.5620	
132	SNP 3	p87723-rs838881	3' flanking	T, 0.3183	TT/CT/CC	63/341/332	0.1390	0.5146		65/344/338	-0.1897	0.7187	
132	SNP 4	p87749-rs76465225	3' flanking	A, 0.0844	AA/GA/GG	7/109/620	-0.1992	0.5583		7/111/629	-0.1205	0.8865	
133	SNP 1	p87694-chr12_125261826	3' flanking	T, 0.002	CC/CT	722/3	3.4021	0.1098	0.5109	731/3	3.0751	0.5620	0.9769
133	SNP 2	p87723-rs838881	3' flanking	T, 0.3183	TT/CT/CC	63/341/332	0.1390	0.5146		65/344/338	-0.1897	0.7187	
133	SNP 3	p87749-rs76465225	3' flanking	A, 0.0844	AA/GA/GG	7/109/620	-0.1992	0.5583		7/111/629	-0.1205	0.8865	
133	SNP 4	p87927-rs838880	3' flanking	A, 0.2414	AA/GA/GG	39/275/418	0.0198	0.9314		40/280/423	-0.2130	0.7056	

ApoA-I, apolipoprotein A-I; del/D, deletion; HDL-C, high-density lipoprotein cholesterol; ins/I, insertion; MA, minor allele; MAF, minor allele frequency; NA, not analyzed; SNP, single nucleotide polymorphism; UTR, untranslated region; W, wild type allele for insertion or deletion on RefSeq.
All alleles on the reverse strand. Splice site is defined as ± 20 bp from the start or end of an exon.
HDL-C and ApoA-I variables were Box-Cox transformed.
Results were adjusted for covariates: sex, age, waist, current smoking (yes/no), and, minutes of daily walking or biking to work (jobmin) for HDL-C; sex and age for ApoA-I.
SNP 1-SNP 4 are in the 5' to 3' direction and shown as "SNP name-SNP ID/Chromosome 12 Position (for novel variants)". All 10 novel variants identified in this study have been submitted to dbSNP database (ID: SCARB1_AB): http://www.ncbi.nlm.nih.gov/SNP/snp_viewTable.cgi?handle=KAMBOH.
Significant global *P*-values (*P* <0.05) are shown in **bold**, see haplotype association plots in Figure 3.
[a, c] RefSeq of *SCARB1*: hg19, NM_005505 (CHIP Bioinformatics).
[b] dbSNP build 139: GRCh37.p10.
[d] Close to a miRNA-145 seed site based on TargetScanHuman (version 6.2, http://www.targetscan.org/).
