# Supplementary material for: Genetic contribution of SCARB1 variants to lipid traits in African Blacks: a candidate gene association study
Source: BMC Med Genet. 2015 Nov 12;16:106. doi: 10.1186/s12881-015-0250-6 (PMC4643515; doi:10.1186/s12881-015-0250-6)
Supplement: Additional file 17: Table S12. — Summary of RegulomeDB scores of 153 SCARB1 variants. (PDF 95 kb) [file 12881_2015_250_MOESM17_ESM.pdf]

Table S12. Summary of RegulomeDB scores of 153 *SCARB1* variants.

RegulomeDB Score[a]	No of Variants (n)						Total N (%)
	MAF ≥5% (n = 101)		MAF between 1-5% (n = 27)		MAF ≤1% (n = 25)		
	Coding	Non-coding	Coding	Non-coding	Coding	Non-coding	
1f		1					1 (0.65)
2a		1		1		1	3 (1.96)
2b	1	4	1	1			7 (4.58)
3a	1	1					2 (1.31)
3b		1					1 (0.65)
4	1	18	1	10		6	36 (23.53)
5	1	40		11	2	13	67 (43.79)
6		12		1		2	15 (9.80)
7		19		1		1	21 (13.73)
**Total N**	**4**	**97**	**2**	**25**	**2**	**23**	**153 (100.00)**

MAF, minor allele frequency; SNP, single nucleotide polymorphism.
A list of 83 variants identified by sequencing is shown in Additional file 3 Table S3.
A list of 68 common HapMap-YRI tagSNPs successfully genotyped in our entire sample is shown in Additional file 7 Table S5.
[a] The RegulomeDB (version 1.0) scoring scheme: **score 1a**, expression quantitative trait loci (eQTL) + transcription factor (TF) binding + matched TF motif + matched DNase Footprint + DNase peak; **score 1b**, eQTL + TF binding + any motif + DNase Footprint + DNase peak; **score 1c**, eQTL + TF binding + matched TF motif + DNase peak; score 1d, eQTL + TF binding + any motif + DNase peak; **score 1e**, eQTL + TF binding + matched TF motif; **score 1f**, eQTL + TF binding / DNase peak, **score 2a**, TF binding + matched TF motif + matched DNase Footprint + DNase peak; **score 2b**, TF binding + any motif + DNase Footprint + DNase peak; **score 2c**, TF binding + matched TF motif + DNase peak; **score 3a**, TF binding + any motif + DNase peak; **score 3b**, TF binding + matched TF motif; **score 4**,TF binding + DNase peak; **score 5**, TF binding or DNase peak; **score 6**, others; **score 7**, no data (see http://regulome.stanford.edu/help).
