# Supplementary material for: Genetic contribution of SCARB1 variants to lipid traits in African Blacks: a candidate gene association study
Source: BMC Med Genet. 2015 Nov 12;16:106. doi: 10.1186/s12881-015-0250-6 (PMC4643515; doi:10.1186/s12881-015-0250-6)
Supplement: Additional file 18: Table S13. — RegulomeDB scores and functional assignments of 153 SCARB1 variants. (PDF 149 kb) [file 12881_2015_250_MOESM18_ESM.pdf]

Table S13. RegulomeDB scores and functional assignments of 153 *SCARB1* variants.

SNP Name[a]	SNP ID[b]	Chr12 Position[c]	Location	Amino Acid Change	Indels	Major/ Minor Alleles	MAF	Assoc Trait(s)[d]	RegDB Score[e]	RegDB Functional Assignments
p972	rs181338950	125348548	5' flanking-promoter			C/T	0.048		2a	Motifs|Footprinting|SP1, Motifs|PWM|Zfp410, Motifs|PWM|SP1, Motifs|PWM|KROX, Motifs|Footprinting|Sp1, Motifs|Footprinting|SP4, Motifs|PWM|UF1H3BETA, Motifs|Footprinting|KROX, Motifs|Footprinting|Klf4, Motifs|PWM|Bcl6b, Motifs|Footprinting|UF1H3BETA, Motifs|PWM|Klf4, Motifs|PWM|Sp1, Motifs|PWM|SP4, Chromatin_Structure|FAIRE, Chromatin_Structure|DNase-seq, Protein_Binding|ChIP-seq|GATA1, Protein_Binding|ChIP-seq|GABPA, Protein_Binding|ChIP-seq|EGR1, Protein_Binding|ChIP-seq|TFAP2C, Protein_Binding|ChIP-seq|TFAP2A, Protein_Binding|ChIP-seq|MYC, Protein_Binding|ChIP-seq|MAX, Protein_Binding|ChIP-seq|IRF1, Protein_Binding|ChIP-seq|ELF1, Protein_Binding|ChIP-seq|SP1, Protein_Binding|ChIP-seq|CCNT2, Protein_Binding|ChIP-seq|TBP, Protein_Binding|ChIP-seq|YY1, Protein_Binding|ChIP-seq|POLR2A, Protein_Binding|ChIP-seq|TAF1, Protein_Binding|ChIP-seq|ZBTB7A, Protein_Binding|ChIP-seq|CTCF
p1048 insC (1048_ 1049)		125348472	Exon 1- 5' UTR		insC	[-/C]	0.0079		2a	Motifs|PWM|HIC1, Motifs|Footprinting|AP-2gamma, Motifs|Footprinting|TFAP2A, Motifs|Footprinting|AP-2alpha, Motifs|Footprinting|HIC1, Motifs|PWM|AP-2alpha, Motifs|PWM|AP-2gamma, Motifs|PWM|AP-2, Motifs|PWM|TFAP2A, Motifs|Footprinting|AP-2, Chromatin_Structure|FAIRE, Chromatin_Structure|DNase-seq, Protein_Binding|ChIP-seq|GATA1, Protein_Binding|ChIP-seq|SIN3A, Protein_Binding|ChIP-seq|HEY1, Protein_Binding|ChIP-seq|EGR1, Protein_Binding|ChIP-seq|TFAP2C, Protein_Binding|ChIP-seq|TFAP2A, Protein_Binding|ChIP-seq|IRF1, Protein_Binding|ChIP-seq|RAD21, Protein_Binding|ChIP-seq|SMC3, Protein_Binding|ChIP-seq|SP1, Protein_Binding|ChIP-seq|CCNT2, Protein_Binding|ChIP-seq|TBP, Protein_Binding|ChIP-seq|POLR2A, Protein_Binding|ChIP-seq|TAF1, Protein_Binding|ChIP-seq|CTCFL, Protein_Binding|ChIP-seq|CTCF, Protein_Binding|ChIP-seq|EBF1, Protein_Binding|ChIP-seq|CDX2
p1257	rs4238001	125348263	Exon 1	Gly2Ser		G/A	0.032		4	Chromatin_Structure|FAIRE, Chromatin_Structure|DNase-seq, Protein_Binding|ChIP-seq|GATA1, Protein_Binding|ChIP-seq|MXI1, Protein_Binding|ChIP-seq|TFAP2C, Protein_Binding|ChIP-seq|TFAP2A, Protein_Binding|ChIP-seq|POU2F2, Protein_Binding|ChIP-seq|RAD21, Protein_Binding|ChIP-seq|CCNT2, Protein_Binding|ChIP-seq|E2F6, Protein_Binding|ChIP-seq|POLR2A, Protein_Binding|ChIP-seq|TAF1, Protein_Binding|ChIP-seq|CDX2
p1265	rs2070242	125348255	Exon 1	Ser4Ser		C/T	0.1284		2b	Motifs|PWM|E2F-1, Motifs|Footprinting|E2F-1, Chromatin_Structure|FAIRE, Chromatin_Structure|DNase-seq,

									Protein_Binding|ChIP-seq|GATA1, Protein_Binding|ChIP-seq|MXI1, Protein_Binding|ChIP-seq|TFAP2A, Protein_Binding|ChIP-seq|POU2F2, Protein_Binding|ChIP-seq|RAD21, Protein_Binding|ChIP-seq|E2F6, Protein_Binding|ChIP-seq|POLR2A, Protein_Binding|ChIP-seq|TAF1, Protein_Binding|ChIP-seq|CDX2
p1316	rs10396208	125348204	Exon 1	Cys21Cys	C/T	0.0476		2b	Motifs|Footprinting|NRSF, Motifs|PWM|REST, Motifs|PWM|NRSF, Motifs|PWM|Osr2, Motifs|Footprinting|REST, Chromatin_Structure|FAIRE, Chromatin_Structure|DNase-seq, Protein_Binding|ChIP-seq|GATA1, Protein_Binding|ChIP-seq|MXI1, Protein_Binding|ChIP-seq|E2F6, Protein_Binding|ChIP-seq|CDX2
p1419	rs201717369	125348101	Intron 1		G/A	0.0121		4	Chromatin_Structure|DNase-seq, Protein_Binding|ChIP-seq|GATA1, Protein_Binding|ChIP-seq|E2F6, Protein_Binding|ChIP-seq|ZBTB7A, Protein_Binding|ChIP-seq|E2F1
p4072	rs7139401	125345448	Intron 1		T/C	0.4386		3b	Motifs|PWM|Mafk, Motifs|PWM|Dlx3, Motifs|PWM|Dlx4, Protein_Binding|ChIP-seq|MAFK
p5055	rs11057869	125344465	Intron 1		G/A	0.0740		7	No data
p6600	rs12831105	125342920	Intron 1		C/T	0.1188		7	No data
p7650	rs11615630	125341870	Intron 1		G/A	0.0436		5	Chromatin_Structure|DNase-seq
p10292	rs4765181	125339228	Intron 1		G/T	0.2490		5	Chromatin_Structure|FAIRE, Chromatin_Structure|DNase-seq
p10991	rs10773112	125338529	Intron 1		G/A	0.3534		4	Chromatin_Structure|FAIRE, Chromatin_Structure|DNase-seq, Protein_Binding|ChIP-seq|FOXA1, Protein_Binding|ChIP-seq|RXRA, Protein_Binding|ChIP-seq|HDAC2
p13570	rs11057864	125335950	Intron 1		G/T	0.1180		4	Chromatin_Structure|FAIRE, Chromatin_Structure|DNase-seq, Protein_Binding|ChIP-seq|HNF4A
p16565	rs10773111	125332955	Intron 1		G/A	0.1928		6	Single_Nucleotides|SCARB1|eQTL
p20207	rs11057853	125329313	Intron 1		G/A	0.4484	HDL-C	5	Motifs|PWM|HNF4, Motifs|PWM|HNF4A, Motifs|PWM|HNF4directrepeat1, Chromatin_Structure|FAIRE, Chromatin_Structure|DNase-seq
p20694	rs11057852	125328826	Intron 1		G/A	0.1050		7	No data
p20741	rs11057851	125328779	Intron 1		C/T	0.3237	HDL-C (best SNP), ApoA-I	5	Chromatin_Structure|DNase-seq
p21145	rs3924313	125328375	Intron 1		C/T	0.1772		6	Motifs|PWM|TFIIA
p22116	rs12370382	125327404	Intron 1		G/A	0.0645		1f	Single_Nucleotides|SCARB1|eQTL, Chromatin_Structure|DNase-seq
p22168	rs7137797	125327352	Intron 1		T/C	0.3977		2b	Motifs|PWM|Staf, Motifs|Footprinting|Staf, Motifs|Footprinting|znf143, Motifs|PWM|znf143, Chromatin_Structure|DNase-seq, Protein_Binding|ChIP-seq|GATA1
p22331	rs6488944	125327189	Intron 1		T/G	0.1474		4	Chromatin_Structure|DNase-seq, Protein_Binding|ChIP-seq|GATA1, Protein_Binding|ChIP-seq|EP300, Protein_Binding|ChIP-seq|GATA2
p22675	rs12425134	125326845	Intron 1		G/T	0.0526		4	Chromatin_Structure|DNase-seq, Protein_Binding|ChIP-seq|GATA1
p28137	rs12229555	125321383	Intron 1		A/G	0.3896		7	No data
p28692	rs4765622	125320828	Intron 1		C/T	0.2565		5	Chromatin_Structure|DNase-seq
p28957	rs11057844	125320563	Intron 1		G/A	0.2362		5	Motifs|PWM|HTF, Motifs|Footprinting|HTF, Protein_Binding|ChIP-seq|GATA1
p29749	rs10846751	125319771	Intron 1		C/T	0.4492		7	No data
p31072	rs10846749	125318448	Intron 1		C/G	0.4461		4	Chromatin_Structure|DNase-seq, Protein_Binding|ChIP-seq|EP300, Protein_Binding|ChIP-seq|MAX, Protein_Binding|ChIP-seq|NANOG, Protein_Binding|ChIP-seq|USF1, Protein_Binding|ChIP-seq|TCF4,

										Protein_Binding|ChIP-seq|SP1, Protein_Binding|ChIP-seq|JUND, Protein_Binding|ChIP-seq|TCF12, Protein_Binding|ChIP-seq|RXRA
p31938	rs10744182	125317582	Intron 1			G/A	0.1837		5	Chromatin_Structure|DNase-seq
p32129	rs10773107	125317391	Intron 1			G/T	0.1009		7	No data
p32273	rs12580803	125317247	Intron 1			T/C	0.1006		5	Chromatin_Structure|DNase-seq
p32290	rs10744181	125317230	Intron 1			T/C	0.1238		5	Motifs|Footprinting|AR, Motifs|PWM|AR, Chromatin_Structure|DNase-seq
p32395	rs12581963	125317125	Intron 1			C/T	0.1314		5	Motifs|PWM|E2F-1, Chromatin_Structure|DNase-seq
p32750	rs7967521	125316770	Intron 1			A/G	0.3425		7	No data
p32777	rs11057841	125316743	Intron 1			G/A	0.2805		7	No data
p32860	rs7967406	125316660	Intron 1			A/C	0.0991		6	Motifs|PWM|Hbp1, Motifs|PWM|Rfx3
p33531	rs11057838	125315989	Intron 1			C/A	0.2278		7	No data
p36094	rs11608336	125313426	Intron 1			G/A	0.1543		4	Chromatin_Structure|FAIRE, Chromatin_Structure|DNase-seq, Protein_Binding|ChIP-seq|HNF4A, Protein_Binding|ChIP-seq|TFAP2C, Protein_Binding|ChIP-seq|TFAP2A, Protein_Binding|ChIP-seq|STAT1, Protein_Binding|ChIP-seq|GTF2F1, Protein_Binding|ChIP-seq|TBP, Protein_Binding|ChIP-seq|NR3C1, Protein_Binding|ChIP-seq|STAT3, Protein_Binding|ChIP-seq|ESR1, Protein_Binding|ChIP-seq|POLR2A, Protein_Binding|ChIP-seq|CDX2
p36461	rs4765178	125313059	Intron 1			C/T	0.1671		4	Chromatin_Structure|FAIRE, Chromatin_Structure|DNase-seq, Protein_Binding|ChIP-seq|HNF4A, Protein_Binding|ChIP-seq|TFAP2A, Protein_Binding|ChIP-seq|NR3C1, Protein_Binding|ChIP-seq|ESR1
p36908	rs10846745	125312612	Intron 1			C/G	0.3257		4	Chromatin_Structure|FAIRE, Chromatin_Structure|DNase-seq, Protein_Binding|ChIP-seq|HNF4A, Protein_Binding|ChIP-seq|EP300, Protein_Binding|ChIP-seq|TFAP2C, Protein_Binding|ChIP-seq|AR, Protein_Binding|ChIP-seq|FOXA1, Protein_Binding|ChIP-seq|CDX2
p37095	rs10846744	125312425	Intron 1			C/G	0.3056		4	Chromatin_Structure|FAIRE, Chromatin_Structure|DNase-seq, Protein_Binding|ChIP-seq|HNF4A, Protein_Binding|ChIP-seq|EP300, Protein_Binding|ChIP-seq|TFAP2C, Protein_Binding|ChIP-seq|MAX, Protein_Binding|ChIP-seq|AR, Protein_Binding|ChIP-seq|ESR1, Protein_Binding|ChIP-seq|FOXA1, Protein_Binding|ChIP-seq|CDX2, Protein_Binding|ChIP-seq|GATA3
p41632	rs6488943	125307888	Intron 1			A/C	0.2954		5	Chromatin_Structure|DNase-seq
p42467	rs11057830	125307053	Intron 1			C/T	0.1523		7	No data
p45516	rs1902569	125304004	Intron 1			G/A	0.1544	HDL-C	5	Chromatin_Structure|DNase-seq
p45627	rs12297372	125303893	Intron 1			A/G	0.0487		5	Chromatin_Structure|DNase-seq
p46964	rs114061302	125302556	Intron 1			G/A	0.0388		4	Chromatin_Structure|DNase-seq, Protein_Binding|ChIP-seq|IKZF1
p48969	rs2343394	125300551	Intron 2			C/T	0.1898		5	Chromatin_Structure|DNase-seq
p49537	rs7305310	125299983	Intron 2			C/T	0.1007		5	Chromatin_Structure|DNase-seq
p49570 delC	rs145376237	125299950	Intron 2		delC	[C/-]	0.2276		5	Motifs|PWM|Ascl2, Motifs|PWM|E47, Motifs|Footprinting|E47, Chromatin_Structure|FAIRE, Chromatin_Structure|DNase-seq
p49690	rs4765615	125299830	Intron 2			G/A	0.4426	HDL-C, ApoA-I	5	Chromatin_Structure|FAIRE, Chromatin_Structure|DNase-seq
p49759	rs146272788	125299761	Intron 2			C/T	0.0020		5	Motifs|PWM|Gm397, Motifs|PWM|Zscan4, Chromatin_Structure|FAIRE, Chromatin_Structure|DNase-seq
p49978	rs5891	125299542	Exon 3	Val135Ile		G/A	0.0058		5	Motifs|Footprinting|Pax-3, Motifs|PWM|Pax-3, Chromatin_Structure|DNase-seq

p50024	rs368880622	125299496	Intron 3		G/T	0.0026		5	Chromatin_Structure|DNase-seq
p50118	rs58710319	125299402	Intron 3		C/T	0.0208		5	Chromatin_Structure|DNase-seq
p50151	rs2278986	125299369	Intron 3		T/C	0.1933		5	Chromatin_Structure|DNase-seq
p50380	rs141748317	125299140	Intron 3		A/G	0.0112		2b	Motifs|Footprinting|HEN1, Motifs|PWM|HEN1, Chromatin_Structure|DNase-seq, Protein_Binding|ChIP-seq|IKZF1
p50489	rs61320152	125299031	Intron 3		G/T	0.0257		4	Chromatin_Structure|DNase-seq, Protein_Binding|ChIP-seq|IKZF1
p50954		125298566	Intron 4		T/C	0.0007		5	Chromatin_Structure|DNase-seq
p51888	rs7138304	125297632	Intron 4		C/T	0.1079		2b	Motifs|Footprinting|TRF1, Motifs|PWM|TRF1, Motifs|Footprinting|IRF-1, Motifs|PWM|IRF-1, Chromatin_Structure|DNase-seq, Protein_Binding|ChIP-seq|NFYB, Protein_Binding|ChIP-seq|USF2, Protein_Binding|ChIP-seq|USF1, Protein_Binding|ChIP-seq|IKZF1
p52096	rs10846739	125297424	Intron 4		A/G	0.4693		3a	Motifs|PWM|Tbp, Chromatin_Structure|DNase-seq, Protein_Binding|ChIP-seq|IKZF1
p52556	rs11057820	125296964	Intron 4		G/A	0.1000		5	Chromatin_Structure|DNase-seq
p52610	rs10846738	125296910	Intron 4		C/T	0.1349		4	Chromatin_Structure|DNase-seq, Protein_Binding|ChIP-seq|SPI1
p52919		125296601	Intron 4		G/T	0.0013		5	Motifs|PWM|Zfp281, Motifs|PWM|MZF1_5-13, Motifs|Footprinting|MZF1_5-13, Motifs|PWM|Gabpa, Chromatin_Structure|FAIRE, Chromatin_Structure|DNase-seq
p52956	rs77740046	125296564	Intron 4		C/T	0.0546		5	Chromatin_Structure|FAIRE, Chromatin_Structure|DNase-seq
p52995	rs113910315	125296525	Intron 4-splice site		T/G	0.0020		5	Motifs|PWM|Zic3, Motifs|PWM|Zic2, Motifs|PWM|Zic1, Chromatin_Structure|FAIRE, Chromatin_Structure|DNase-seq
p53128	rs372212527	125296392	Intron 5		C/T	0.005		5	Chromatin_Structure|FAIRE, Chromatin_Structure|DNase-seq
p53159	rs59809936	125296361	Intron 5		G/A	0.049		5	Chromatin_Structure|FAIRE, Chromatin_Structure|DNase-seq
p53359	rs112371713	125296161	Intron 5		G/A	0.1243		5	Chromatin_Structure|DNase-seq
p53372	rs115604379	125296148	Intron 5		C/T	0.0066		5	Chromatin_Structure|DNase-seq
p53481	rs143611171	125296039	Intron 5		C/T	0.099		6	Motifs|Footprinting|ARP-1(COUP-TF2), Motifs|PWM|ARP-1(COUP-TF2)
p53790	rs4765614	125295730	Intron 5		G/A	0.2653		5	Chromatin_Structure|DNase-seq
p54445	rs60910935	125295075	Intron 5		A/G	0.0418		4	Chromatin_Structure|DNase-seq, Protein_Binding|ChIP-seq|RFX3
p54475	rs60227139	125295045	Intron 5		C/T	0.0437		4	Chromatin_Structure|DNase-seq, Protein_Binding|ChIP-seq|RFX3
p54492	rs61762481	125295028	Intron 5		G/A	0.1005		4	Chromatin_Structure|DNase-seq, Protein_Binding|ChIP-seq|RFX3
p54611		125294909	Intron 5		T/C	0.0007		4	Chromatin_Structure|DNase-seq, Protein_Binding|ChIP-seq|RFX3
p54627		125294893	Intron 5		G/C	0.0020		4	Chromatin_Structure|DNase-seq, Protein_Binding|ChIP-seq|RFX3
p54856		125294664	Intron 6		C/T	0.0007		4	Chromatin_Structure|DNase-seq, Protein_Binding|ChIP-seq|RFX3, Protein_Binding|ChIP-seq|STAT3
p55923	rs838900	125293597	Intron 6		G/A	0.3921		7	No data
p55963	rs7134858	125293557	Intron 6		C/T	0.1560	ApoA-I (best SNP)	6	Motifs|PWM|STAT1
p56845	rs838902	125292675	Intron 6		A/G	0.4249		5	Motifs|PWM|Bbx, Chromatin_Structure|DNase-seq
p57004	rs187562853	125292516	Intron 6		G/A	0.0098		4	Chromatin_Structure|DNase-seq, Protein_Binding|ChIP-seq|GATA1
p57107	rs5892	125292413	Exon 7	Phe301Phe	C/T	0.0589		4	Chromatin_Structure|FAIRE, Chromatin_Structure|DNase-seq, Protein_Binding|ChIP-seq|GATA1, Protein_Binding|ChIP-seq|NR3C1
p57508	rs71458866	125292012	Intron 7		G/A	0.1130		4	Chromatin_Structure|FAIRE, Chromatin_Structure|DNase-seq, Protein_Binding|ChIP-seq|GATA1
p57592	rs838903	125291928	Intron 7		G/A	0.3763		4	Chromatin_Structure|DNase-seq, Protein_Binding|ChIP-seq|GATA1

p58514	rs838905	125291006	Intron 7			T/C	0.4329		4	Chromatin_Structure|DNase-seq, Protein_Binding|ChIP-seq|CEBPB
p58664	rs865716	125290856	Intron 7			A/T	0.2708		5	Chromatin_Structure|DNase-seq
p60255	rs3782287	125289265	Intron 7			C/T	0.2831		5	Chromatin_Structure|DNase-seq
p61872	rs838909	125287648	Intron 7			C/T	0.2199		4	Chromatin_Structure|DNase-seq, Protein_Binding|ChIP-seq|RFX3
p62140	rs838910	125287380	Intron 7			G/T	0.3047		5	Chromatin_Structure|FAIRE, Protein_Binding|ChIP-seq|RFX3
p62409	rs838911	125287111	Intron 7			C/T	0.4211		5	Chromatin_Structure|FAIRE, Chromatin_Structure|DNase-seq
p62615	rs7138386	125286905	Intron 7			T/C	0.1137		5	Chromatin_Structure|FAIRE, Chromatin_Structure|DNase-seq
p63483	rs838912	125286037	Intron 7			G/A	0.0867	ApoA-I	7	No data
p64772	rs5888	125284748	Exon 8	Ala350Ala		C/T	0.0961	ApoA-I	3a	Motifs|PWM|Nr2f2, Motifs|PWM|Rara, Motifs|PWM|PPARalpha:RXRalpha, Chromatin_Structure|DNase-seq, Protein_Binding|ChIP-seq|RFX3, Protein_Binding|ChIP-seq|GATA2
p64923	rs838915	125284597	Intron 8			C/A	0.1435		5	Motifs|PWM|AP-2, Chromatin_Structure|DNase-seq
p65999	rs12819677	125283521	Intron 8			G/A	0.2813		6	Motifs|PWM|PPARG
p67439	rs961170	125282081	Intron 8			G/A	0.0893		4	Chromatin_Structure|DNase-seq, Protein_Binding|ChIP-seq|SPI1
p67700	rs1726374	125281820	Intron 8			G/A	0.1933		7	No data
p69013	rs7135117	125280507	Intron 8			A/G	0.2901		7	No data
p69699	rs10396210	125279821	Intron 8- splice site			G/A	0.1511		4	Chromatin_Structure|FAIRE, Chromatin_Structure|DNase-seq, Protein_Binding|ChIP-seq|NFKB1, Protein_Binding|ChIP-seq|TCF12, Protein_Binding|ChIP-seq|POLR2A, Protein_Binding|ChIP-seq|EBF1
p69994	rs188094006	125279526	Intron 9			C/T	0.005		5	Motifs|PWM|RREB-1, Motifs|PWM|GR, Chromatin_Structure|DNase-seq
p69995 delC	rs5801571	125279525	Intron 9		delC	[C/-]	0.2761		5	Motifs|PWM|RREB-1, Motifs|PWM|GR, Chromatin_Structure|DNase-seq
p70088	rs117585141	125279432	Intron 9			G/A	0.158		7	No data
p70129	rs1814575	125279391	Intron 9			C/T	0.047		7	No data
p70148	rs183061101	125279372	Intron 9			T/C	0.011		6	Motifs|PWM|Nrf-2, Motifs|PWM|LUN-1
p70201		125279319	Intron 9			T/C	0.0010		6	Motifs|PWM|MEF2A
p70292	rs150388176	125279228	Intron 9			C/T	0.016		5	Chromatin_Structure|DNase-seq
p71867	rs7954022	125277653	Intron 9			C/T	0.1323		5	Motifs|PWM|EWSR1-FLI1, Motifs|Footprinting|EWSR1-FLI1, Chromatin_Structure|DNase-seq
p72197	rs838861	125277323	Intron 9			A/G	0.3777		7	No data
p72777	rs838862	125276743	Intron 9			C/T	0.0887		5	Chromatin_Structure|DNase-seq
p75766	rs838866	125273754	Intron 9			T/C	0.2116		6	Single_Nucleotides|SCARB1|eQTL
p75778	rs7301120	125273742	Intron 9			C/T	0.1135		6	Single_Nucleotides|SCARB1|eQTL
p76204	rs866793	125273316	Intron 9			A/G	0.0520		7	No data
p76757	rs9919713	125272763	Intron 9			A/T	0.4390		6	Motifs|PWM|FOXJ2, Motifs|PWM|Alx-4, Motifs|Footprinting|FOXJ2, Motifs|Footprinting|Alx-4
p77181	rs146246031	125272339	Intron 9			T/C	0.0053		7	No data
p77250	rs201901986	125272270	Intron 9			G/T	0.095		6	Motifs|PWM|Srf, Motifs|PWM|Mtf1, Motifs|PWM|Tcfap2e
p77251	rs34339961	125272269	Intron 9			A/T	0.1177		6	Motifs|PWM|Srf, Motifs|PWM|Mtf1, Motifs|PWM|Tcfap2e
p77381	rs138499966	125272139	Intron 9			T/C	0.0046		6	Motifs|PWM|Foxl1, Motifs|PWM|Freac-2, Motifs|PWM|Foxj3, Motifs|PWM|FOXO3, Motifs|PWM|HFH8(FOXF1A), Motifs|PWM|FOXJ2, Motifs|PWM|Freac-4, Motifs|Footprinting|Foxq1, Motifs|PWM|Foxa2, Motifs|PWM|HFH1(FOXQ1), Motifs|PWM|Foxk1,

									Motifs|Footprinting|HFH1(FOXQ1), Motifs|PWM|Foxq1, Motifs|PWM|HNF3
p77620	rs377124254	125271900	Intron 10		G/A	0.0007		5	Chromatin_Structure|DNase-seq
p77682	rs150082885	125271838	Intron 10		A/G	0.0106		5	Chromatin_Structure|DNase-seq
p77704		125271816	Intron 10		C/A	0.0040		5	Chromatin_Structure|DNase-seq
p77842	rs2272310	125271678	Intron 10		G/A	0.0807		5	Chromatin_Structure|DNase-seq
p78255	rs184052375	125271265	Intron 10		A/G	0.0072		4	Chromatin_Structure|FAIRE, Chromatin_Structure|DNase-seq, Protein_Binding|ChIP-seq|SIX5, Protein_Binding|ChIP-seq|MAX, Protein_Binding|ChIP-seq|NANOG, Protein_Binding|ChIP-seq|USF2, Protein_Binding|ChIP-seq|USF1, Protein_Binding|ChIP-seq|ATF3, Protein_Binding|ChIP-seq|YY1, Protein_Binding|ChIP-seq|TBP, Protein_Binding|ChIP-seq|POLR2A, Protein_Binding|ChIP-seq|TAF1
p78402	rs838898	125271118	Intron 10		G/A	0.0714		5	Motifs|PWM|Hic1, Motifs|PWM|Eomes, Chromatin_Structure|FAIRE, Chromatin_Structure|DNase-seq
p78430	rs838897	125271090	Intron 10		C/G	0.3830		5	Motifs|PWM|RNF96, Motifs|PWM|AP-2, Motifs|Footprinting|AP-2, Chromatin_Structure|FAIRE, Chromatin_Structure|DNase-seq
p78747	rs2293440	125270773	Intron 11		T/C	0.4112		5	Chromatin_Structure|DNase-seq
p78791	rs75289200	125270729	Intron 11		T/C	0.0321		5	Chromatin_Structure|DNase-seq
p79721	rs838896	125269799	Intron 11		G/C	0.3104	ApoA-I	5	Chromatin_Structure|DNase-seq
p79828	rs838895	125269692	Intron 11		C/G	0.3171	ApoA-I, HDL-C	5	Motifs|PWM|Pax-5, Chromatin_Structure|DNase-seq
p80045	rs838893	125269475	Intron 11		G/A	0.3244		5	Chromatin_Structure|DNase-seq
p81863	rs185445624	125267657	Intron 11		G/A	0.0020		5	Motifs|PWM|ESR1, Motifs|PWM|Rfx3, Chromatin_Structure|FAIRE, Chromatin_Structure|DNase-seq
p82019	rs838890	125267501	Intron 11		C/T	0.0320		5	Chromatin_Structure|FAIRE, Chromatin_Structure|DNase-seq
p82264	rs141545424	125267256	Exon 12	Gly501Gly	C/A	0.0007		5	Chromatin_Structure|DNase-seq
p82340	rs77483223	125267180	Intron 12		G/A	0.0231		5	Motifs|PWM|Arid3a, Chromatin_Structure|DNase-seq
p82369	rs75446635	125267151	Intron 12		G/A	0.0059		5	Chromatin_Structure|DNase-seq
p82434	rs838889	125267086	Intron 12		T/C	0.0315		5	Motifs|PWM|FOXP1, Motifs|Footprinting|HFH3(FOXI1), Motifs|PWM|MRF-2, Motifs|Footprinting|MRF-2, Motifs|PWM|HFH3(FOXI1), Chromatin_Structure|DNase-seq
p83547	rs838887	125265973	Intron 12		C/G	0.4564		5	Chromatin_Structure|DNase-seq
p83884	rs701106	125265636	Intron 12		C/T	0.2597	ApoA-I	5	Chromatin_Structure|DNase-seq
p86245	rs188375019	125263275	Intron 12		C/T	0.0341		4	Chromatin_Structure|FAIRE, Chromatin_Structure|DNase-seq, Protein_Binding|ChIP-seq|BATF
p86276	rs747155	125263244	Intron 12		C/T	0.1495		2b	Motifs|PWM|NGFI-C, Motifs|Footprinting|NGFI-C, Motifs|Footprinting|Sp3, Motifs|PWM|Sp3, Motifs|PWM|KROX, Motifs|PWM|UF1H3BETA, Motifs|Footprinting|KROX, Motifs|Footprinting|UF1H3BETA, Chromatin_Structure|FAIRE, Chromatin_Structure|DNase-seq, Protein_Binding|ChIP-seq|BATF
p86316	rs701104	125263204	Intron 12		G/T	0.0487		4	Chromatin_Structure|FAIRE, Chromatin_Structure|DNase-seq, Protein_Binding|ChIP-seq|EGR1, Protein_Binding|ChIP-seq|BATF
p86481	rs701103	125263039	Exon 13-3' UTR	Gly499Arg (isoform 2)	G/A	0.2451		5	Chromatin_Structure|DNase-seq
p86967	rs187492239	125262553	Exon 13-3' UTR		A/G	0.0355		4	Chromatin_Structure|FAIRE, Chromatin_Structure|DNase-seq, Protein_Binding|ChIP-seq|HNF4A, Protein_Binding|ChIP-seq|HEY1, Protein_Binding|ChIP-seq|TCF4,

							Protein_Binding|ChIP-seq|JUND, Protein_Binding|ChIP-seq|POLR2A, Protein_Binding|ChIP-seq|HNF4G, Protein_Binding|ChIP-seq|FOSL2, Protein_Binding|ChIP-seq|HDAC2
p87011	rs58032386	125262509	Exon 13-3' UTR	C/T	0.1417	2a	Motifs|PWM|HNF4alpha1, Motifs|Footprinting|HNF4alpha1, Chromatin_Structure|FAIRE, Chromatin_Structure|DNase-seq, Protein_Binding|ChIP-seq|HNF4A, Protein_Binding|ChIP-seq|HEY1, Protein_Binding|ChIP-seq|TCF4, Protein_Binding|ChIP-seq|JUND, Protein_Binding|ChIP-seq|POLR2A, Protein_Binding|ChIP-seq|HNF4G, Protein_Binding|ChIP-seq|FOSL2, Protein_Binding|ChIP-seq|HDAC2
p87195	rs10396211	125262325	Exon 13-3' UTR	G/C	0.122	4	Chromatin_Structure|DNase-seq, Protein_Binding|ChIP-seq|TCF4
p87266	rs150512235	125262254	Exon 13-3' UTR[f]	T/C	0.0057	4	Chromatin_Structure|DNase-seq, Protein_Binding|ChIP-seq|ELF1
p87416	rs838884	125262104	3' flanking	C/T	0.301	2b	Motifs|PWM|DEAF1, Motifs|Footprinting|DEAF1, Chromatin_Structure|DNase-seq, Protein_Binding|ChIP-seq|CEBPB, Protein_Binding|ChIP-seq|ELF1, Protein_Binding|ChIP-seq|FOXA2, Protein_Binding|ChIP-seq|JUND, Protein_Binding|ChIP-seq|FOSL2, Protein_Binding|ChIP-seq|FOXA1
p87459		125262061	3' flanking	C/T	0.005	4	Chromatin_Structure|FAIRE, Chromatin_Structure|DNase-seq, Protein_Binding|ChIP-seq|CEBPB, Protein_Binding|ChIP-seq|ELF1, Protein_Binding|ChIP-seq|FOXA2, Protein_Binding|ChIP-seq|JUND, Protein_Binding|ChIP-seq|FOSL2, Protein_Binding|ChIP-seq|FOXA1
p87611	rs190688220	125261909	3' flanking	C/T	0.0316	4	Chromatin_Structure|DNase-seq, Protein_Binding|ChIP-seq|CEBPB, Protein_Binding|ChIP-seq|JUND, Protein_Binding|ChIP-seq|FOSL2
p87681	rs838883	125261839	3' flanking	G/A	0.0459	5	Chromatin_Structure|FAIRE, Chromatin_Structure|DNase-seq
p87694		125261826	3' flanking	C/T	0.0020	5	Motifs|PWM|TP53, Motifs|PWM|p53, Chromatin_Structure|FAIRE, Chromatin_Structure|DNase-seq
p87707	rs838882	125261813	3' flanking	G/A	0.303	7	No data
p87723	rs838881	125261797	3' flanking	C/T	0.3183	6	Motifs|PWM|NFIL3, Motifs|Footprinting|NFIL3, Motifs|PWM|TFIIA, Chromatin_Structure|FAIRE
p87749	rs76465225	125261771	3' flanking	G/A	0.0844	7	No data
p87927	rs838880	125261593	3' flanking	G/A	0.2414	5	Chromatin_Structure|DNase-seq

ApoA-I, apolipoprotein A-I; HDL-C, high-density lipoprotein cholesterol; del, deletion; eQTL, expression quantitative trait loci; Indels, insertion and deletion variations; ins, insertion; MAF, minor allele frequency; RegDB, RegulomeDB score; SNP, single nucleotide polymorphism; UTR, untranslated region.
All alleles on the reverse strand. Splice site is defined as ± 20 bp from the start or end of an exon.
A list of 83 variants identified by sequencing is shown in Additional file 3 Table S3.
A list of 68 common HapMap-YRI tagSNPs successfully genotyped in our entire sample is shown in Additional file 7 Table S5.
A list of 138 genotyped variants in our entire sample is shown in Additional file 9 Table S6.
Underlined MAFs are based on our sequencing data/sample due to genotyping failure (Additional file 3 Table S3).
Double underlined MAFs are identified in our sequencing stage but not selected for genotyping based on the linkage disequilibrium plot (Additional file 3 Table S3; Additional file 5 Figure S1 and Additional file 6 Figure S2).
All 10 novel variants identified in this study have been submitted to dbSNP database (batch ID: SCARB1_AB): http://www.ncbi.nlm.nih.gov/SNP/snp_viewTable.cgi?handle=KAMBOH.
[a, c] RefSeq of *SCARB1*: hg19, NM_005505 (CHIP Bioinformatics).
[b] dbSNP build 139: GRCh37.p10.
[d] Evidence is based on SNPs with MAF ≥5% exhibiting nominally significant association with either HDL-C or ApoA-I (*P* <0.05; Additional file 14 Table S9 and Additional file 15 Table S10) in single-site analysis observed in the current study.
[e] The RegulomeDB (version 1.0) scoring scheme is described at the footnote of Additional file 17 Table S12 or can be seen at http://regulome.stanford.edu/help.
[f] Close to a miRNA-145 seed site based on TargetScanHuman (version 6.2, http://www.targetscan.org/).
